# Extreme genome diversity in the hyper-prevalent parasitic eukaryote *Blastocystis*

**DOI:** 10.1371/journal.pbio.2003769

**Published:** 2017-09-11

**Authors:** Eleni Gentekaki, Bruce A. Curtis, Courtney W. Stairs, Vladimír Klimeš, Marek Eliáš, Dayana E. Salas-Leiva, Emily K. Herman, Laura Eme, Maria C. Arias, Bernard Henrissat, Frédérique Hilliou, Mary J. Klute, Hiroshi Suga, Shehre-Banoo Malik, Arthur W. Pightling, Martin Kolisko, Richard A. Rachubinski, Alexander Schlacht, Darren M. Soanes, Anastasios D. Tsaousis, John M. Archibald, Steven G. Ball, Joel B. Dacks, C. Graham Clark, Mark van der Giezen, Andrew J. Roger

**Affiliations:** 1 Department of Biochemistry & Molecular Biology, Dalhousie University, Halifax, Nova Scotia, Canada; 2 Centre for Comparative Genomics and Evolutionary Bioinformatics, Dalhousie University, Halifax, Nova Scotia, Canada; 3 Department of Biology and Ecology, Faculty of Science, University of Ostrava, Ostrava, Czech Republic; 4 Department of Cell Biology, University of Alberta, Edmonton, Alberta, Canada; 5 Université des Sciences et Technologies de Lille, Unité de Glycobiologie Structurale et Fonctionnelle, UMR8576 CNRS-USTL, Cité Scientifique, Villeneuve d’Ascq Cedex, France; 6 CNRS UMR 7257, Aix-Marseille University, Marseille, France; 7 INRA, USC 1408 AFMB, Marseille, France; 8 Department of Biological Sciences, King Abdulaziz University, Jeddah, Saudi Arabia; 9 Université Côte d'azur, INRA, ISA, Sophia Antipolis, France; 10 Faculty of Life and Environmental Sciences, Prefectural University of Hiroshima, Nanatsuka 562, Shobara, Hiroshima, Japan; 11 College of Life and Environmental Sciences, University of Exeter, Exeter, United Kingdom; 12 Canadian Institute for Advanced Research, CIFAR Program in Integrated Microbial Biodiversity, Toronto, Canada; 13 Faculty of Infectious and Tropical Diseases, London School of Hygiene and Tropical Medicine, London, United Kingdom; 14 Biosciences, University of Exeter, Exeter, United Kingdom; Duke University Medical Center, United States of America

## Abstract

*Blastocystis* is the most prevalent eukaryotic microbe colonizing the human gut, infecting approximately 1 billion individuals worldwide. Although *Blastocystis* has been linked to intestinal disorders, its pathogenicity remains controversial because most carriers are asymptomatic. Here, the genome sequence of *Blastocystis* subtype (ST) 1 is presented and compared to previously published sequences for ST4 and ST7. Despite a conserved core of genes, there is unexpected diversity between these STs in terms of their genome sizes, guanine-cytosine (GC) content, intron numbers, and gene content. ST1 has 6,544 protein-coding genes, which is several hundred more than reported for ST4 and ST7. The percentage of proteins unique to each ST ranges from 6.2% to 20.5%, greatly exceeding the differences observed within parasite genera. Orthologous proteins also display extreme divergence in amino acid sequence identity between STs (i.e., 59%–61% median identity), on par with observations of the most distantly related species pairs of parasite genera. The STs also display substantial variation in gene family distributions and sizes, especially for protein kinase and protease gene families, which could reflect differences in virulence. It remains to be seen to what extent these inter-ST differences persist at the intra-ST level. A full 26% of genes in ST1 have stop codons that are created on the mRNA level by a novel polyadenylation mechanism found only in *Blastocystis*. Reconstructions of pathways and organellar systems revealed that ST1 has a relatively complete membrane-trafficking system and a near-complete meiotic toolkit, possibly indicating a sexual cycle. Unlike some intestinal protistan parasites, *Blastocystis* ST1 has near-complete de novo pyrimidine, purine, and thiamine biosynthesis pathways and is unique amongst studied stramenopiles in being able to metabolize α-glucans rather than β-glucans. It lacks all genes encoding heme-containing cytochrome P450 proteins. Predictions of the mitochondrion-related organelle (MRO) proteome reveal an expanded repertoire of functions, including lipid, cofactor, and vitamin biosynthesis, as well as proteins that may be involved in regulating mitochondrial morphology and MRO/endoplasmic reticulum (ER) interactions. In sharp contrast, genes for peroxisome-associated functions are absent, suggesting *Blastocystis* STs lack this organelle. Overall, this study provides an important window into the biology of *Blastocystis*, showcasing significant differences between STs that can guide future experimental investigations into differences in their virulence and clarifying the roles of these organisms in gut health and disease.

## Author summary

*Blastocystis* are unicellular eukaryotic organisms related to algae and some plant pathogens. They are common constituents of the human gut microbial community, colonizing approximately 1 billion humans worldwide. Whether their presence is harmful or not continues to be hotly debated. Part of the uncertainty stems from the fact that at least 17 subtypes have been identified from various mammalian hosts, including 9 from humans. To better characterize and understand *Blastocysti*s, we have sequenced and annotated the genome for subtype 1 and compared it with previous genomic results for subtypes 7 and 4. The comparisons revealed considerable differences between the 3 sequenced subtypes for a number of genomic features like DNA base composition, size of genome, number of genes, and number of introns. We also examined various biochemical pathways and cellular systems in the context of the full gene complement to better understand the biology of *Blastocystis*, including some of its more unusual features like a mitochondrion related organelle. We also identified subtype-specific gene family expansions that may be related to virulence. Finally, we showed that *Blastocystis* appears to have most of the genes necessary for sexual reproduction. This study provides resources and hypotheses for future investigations into the biology and potential pathogenicity of these common gut microbes.

## Introduction

*Blastocystis* is a genus of atypical, nonflagellated, anaerobic stramenopiles commonly inhabiting the intestinal tract of humans and other animals. The majority of stramenopiles, which, along with the Alveolata and Rhizaria, are members of the eukaryotic supergroup known as SAR, are marine biflagellated cells with tubular hairs on their surface—*Blastocystis* has none of these characteristics. The number of humans infected with *Blastocystis* globally has been estimated at over 1 billion [1], with prevalence being higher in developing than in developed countries [2]. The infective stage of *Blastocystis* is an environmentally resistant cyst, with the most common mode of transmission being the fecal-oral route. The pathogenicity of *Blastocystis* is controversial, but colonization with this organism has been linked to gastrointestinal symptoms, including diarrhea, abdominal pain, nausea, and irritable bowel syndrome [3]. *Blastocystis* is capable of becoming established in the gut and is difficult to eradicate via pharmacological interventions [3]. However, a causal link between the presence of the organism and disease symptoms has not been established [1,3], and some authors argue that *Blastocystis* is a part of a healthy gut microbiota [4].

At the genetic level, *Blastocystis* is remarkably heterogeneous. Many morphologically similar but genetically distinct lineages of *Blastocystis* have been identified, based primarily on sequences of their small subunit (SSU) ribosomal RNA genes [5]. Seventeen lineages have been isolated from mammals and birds to date and are referred to as “subtypes” (STs); the inter-ST divergence of the SSU rRNA gene is at least 3%. *Blastocystis* STs can have a remarkably broad host range and are almost never found exclusively in 1 host [5,6]. Only STs 1–9 have been found in humans to date, with STs 1–4 being responsible for around 90% of all human cases examined [6].

The high degree of genetic diversity is a confounding factor in establishing whether *Blastocystis* is a pathogen. Recent in vitro and in vivo molecular investigations have identified hydrolases and proteases as candidate virulence factors. *Blastocystis* proteases cause cleavage and degradation of immunoglobulin A (IgA) secreted by the host, may disrupt the intestinal epithelial barrier, and increase production of pro-inflammatory cytokines [2,7,8]. However, there is substantial inter-ST variation in adhesion to enterocytes, disruption of intestinal epithelial tight junctions, activation of pro-inflammatory cytokines, and the ability to scavenge nitric oxide. Virulence factor variability presumably extends to the intra-ST level [9] because the same ST is commonly found in both symptomatic and asymptomatic hosts. The highly variable clinical presentations attributed to *Blastocystis* could potentially be due to colonization with different STs or strains of the organism and untangling the relationships remains an area of active research and concern [10–12].

Recently, researchers have begun to assess the relationship between *Blastocystis* colonization and the composition of the prokaryotic gut microbiota. While several studies indicate *Blastocystis* may be associated with a more diverse and “healthy” microbiota [13,14], others have reported an association between *Blastocystis* colonization and a decrease in protective bacteria in the gut [15] or no differences in microbiota between *Blastocystis*-positive and -negative patients [16].

Despite the unanswered questions regarding its potential clinical relevance, studies of *Blastocystis* genomes are still in their infancy. Currently, several mitochondrial genomes (STs 1–4 and 6–9 [17–20]) and 2 high-quality draft nuclear genomes (STs 4 and 7 [21,22]) are available, as are genome survey data for additional STs (STs 2, 3, 6, 8, and 9 [13]).

The ST7 *Blastocystis* nuclear genome, obtained using a culture established from fecal matter from a symptomatic human, was the first to be sequenced [21]. The study reported a genome size of 18.8 Mb with 6,020 protein-coding genes. The authors described, among other things, a complex "mitochondria-like" organelle, effector proteins possibly involved in adaptation to a parasitic lifestyle, and a suite of secretory proteins that could have the potential to alter host physiology. They also detailed the genome architecture of ST7, finding it to be highly compact and having numerous duplicated blocks of genes.

More recently, an ST4 genome obtained from a laboratory rodent in Singapore was published [22]; its size was 12.91 Mb and a set of 5,713 protein-coding genes was predicted. There were no detailed analyses of the genome structure or of the genes themselves. In 2015, draft assemblies for the nuclear genomes of STs 2, 3, 4, 6, 8, and 9 were released to public databases. None of these assemblies have been annotated with predicted genes and no analyses have been reported to date beyond their use in a microbiome analysis [13].

Here, we report a draft genome sequence and transcriptome analysis of *Blastocystis* ST1, NandII strain from a symptomatic human, and compare it to the published genomes of *Blastocystis* ST7 [21] and ST4 [22]. We first provide a general overview and statistics for the NandII genome and perform high-level comparisons between the 3 genomes. We then present findings derived from extensive manual annotation, with a focus on genes that are potential host effectors, and highlight significant genomic differences between the STs. Our study provides a strong framework for subsequent molecular and cellular investigations of the role of *Blastocystis* in gastrointestinal health and disease and of its impact on the microbial communities of the gut.

## Results and discussion

### Genome assembly

The assembled size of the *Blastocystis* ST1 genome is 16.5 Mb spread across 580 scaffolds (Table 1). In comparison, the assembly of ST7 is composed of 54 scaffolds and 18.8 Mb, while the ST4 assembly is 12.9 Mb and has 1,301 scaffolds. The substantially lower number of scaffolds in the ST7 assembly reflects the longer reads of the Sanger sequencing technology used versus the shorter reads generated in next-generation sequencing (454 and Illumina) used in the cases of ST1 and ST4. Although the assembly for ST7 is substantially larger with longer scaffolds, the fold coverage, gene complement, and transcriptome coverage suggest that the difference in size between the strains is real.

**Table 1:** Genome statistics for *Blastocystis* ST1, ST4, and ST7.

Genomic features	ST1	ST4	ST7
Genome assembly size (Mb)	16.5	12.9	18.8
Scaffolds	580	1,301	54
GC content (%)	54.6	39.6	45.2
Number of protein-coding genes	6,544	5,713	6,020
Missing genes (%)—BUSCO	14.6	16.7	20
Average gene size (bp)	1,760	1,386	1,296
Genes/kb	0.39	0.44	0.32
Genes with introns (%)	94.6	92.7	84.6
Average exons per gene	6.45	5.06	4.58
Mean length of introns (bp)	50	33	50
Mode length of introns (bp)	30 (54%)	30 (36%)	30 (21%)
Number of introns	35,412	24,093	18,200
Average length of proteins (amino acids)	499	416	359
Number of forward genes	3,261	2,815	3,005
Number of reverse genes	3,283	2,898	3,015
Intergenic spacer size (bp) (average/median)	615/224	664/307	1,687/546
Exonic/intronic portions (%)	59/10	55/6	34/5
Number of overlapping genes	0	95	5

**Abbreviations:** BUSCO, Benchmarking Universal Single-Copy Orthologs; ST, subtype

The substantial genomic differences between ST1, ST4, and ST7 are not limited to size. The GC content varies by 15% between the 3 STs (39.6% in ST4, 45.2% in ST7, and 54.6% in ST1), which is significantly different when compared to GC-content variation in some parasites: the GC content difference among 3 strains of *Giardia* is only 2% [23], as is the difference among 4 species of *Leishmania* [24]. More biologically relevant is the variation among the *Blastocystis* STs in terms of overall gene numbers and gene structure. ST1 has 524 more protein-coding genes than were identified in ST7 and 831 more than in ST4 (Table 1). ST1 has the largest average gene size, the highest percentage of genes with introns, the highest average number of exons per gene, and the largest percentage of its genome devoted to encoding proteins, whereas ST7 has the lowest figures for these same attributes (Table 1). Although ST7 was characterized as being a compact genome [21], ST1 is demonstrably more compact, with an average intergenic spacer size of 615 bps versus 1,687 bps in ST7. ST4 tends to fall in the middle for these various measures, although it has more genes/kb, 0.44, than ST1 (0.39) or ST7 (0.32) because it has the smallest genome size.

While these differences in the number and organization of genes in different STs likely reflect real differences between their genomes, they may to some extent be impacted by the procedures used to predict gene models in the 3 genome assemblies. Particularly noteworthy is that ST7 and ST4 were annotated before the recognition of the phenomenon of polyadenylation-mediated creation of stop codons that occurs in *Blastocystis* (see below). Hence, the statistics concerning the different genomic elements in ST7 and ST4 (e.g., genes, exons, introns, intergenic spaces) reported in this and subsequent sections must be considered current estimates that may change after revised annotation.

### Functional termination codons

A striking structural feature of *Blastocystis* STs that is unique amongst eukaryotic nuclear genomes is the polyadenylation-mediated generation of termination codons [25]. This process involves the creation of a functional stop codon by 3′-polyadenylation of mRNAs in a subset of genes, whereby the first 1 or 2 adenines of the poly-A tail appended to terminal uracil-adenine/uracil-guanine or uracil respectively, complete a termination codon missing in the actual gene sequence. Importantly, the addition of the poly-A tail occurs upstream of any possible canonical stop site in the underlying genomic sequence in these genes. Because most gene-finding algorithms are predicated on the presence of standard stop codons, this unique feature in *Blastocystis* can lead to erroneous gene models with problems such as overly long protein sequences, overlapping genes, chimeric genes, and the introduction of false introns to reach the next downstream stop codon.

The phenomenon was first investigated in ST7 using expressed sequence tag (EST) data [25]. The authors concluded that potentially 15% of ST7 genes have stop codons generated by polyadenylation. They also found some examples of the same process in a preliminary draft genome of ST1. With the full genome of ST1 available, as well as RNA-sequencing (RNA-Seq) data, we investigated the extent of the phenomenon. Appropriate stop codons generated by polyadenylation were found in 1,693 protein-coding genes, representing 26% of the protein-coding genes in ST1. The 15% suggested for ST7 may be an underestimate because mapped RNA-Seq data are far superior to ESTs for confirming whether the stop codon is generated via polyadenylation.

Because this phenomenon was not identified prior to the gene-finding process for the *Blastocystis* ST7 genome, a number of the initial gene models were incorrect [25]. At present, it is unclear whether the gene models for ST4 suffer from similar problems or even whether the same polyadenylation-mediated generation of termination codons occurs. The sites for polyadenylation appear to be linked to a highly conserved motif (TGTTTGTT) usually found 5 bases downstream of the nucleotide preceding the poly-A tail. A search for this motif indicates that it is abundant (Table 2) in *Blastocystis*. All 3 genomes have roughly the same number of motifs when genome size is taken into account. ST1 has 1 site per 3,097 bps, ST4 has 1 per 2,751 bps, and ST7 has 1 per 2,782 bps. A random selection of other stramenopile genomes showed that *Thalassiosira pseudonana* has 1 motif per 17,754 bps, while *Phytophthora infestans* has 1 site per 37,441 bps, suggesting that in these 2 genomes, the presence of the motif is random.

**Table 2:** Conserved polyadenylation motif sites (TGTTTGTT) in the genomes of *Blastocystis* ST1, ST4, and ST7.

Genomic feature	ST1	ST4	ST7	Tp	Pi
Forward strand sites	2,620	2,320	3,329	954	3,069
Reverse strand sites	2,707	2,368	3,403	873	3,035
1 Site/length (bp)	1/3,097	1/2,751	1/2,792	1/17,754	1/37,441

**Abbreviations:** Pi, *Phytophthora infestans*; Tp, *Thalassiosira pseudonana*

Because ST4 has a very similar motif complement to ST1 and ST7, it is likely that it too uses the polyadenylation process to generate some of its termination codons. Also suggestive are the 95 gene model pairs that overlap in ST4 (Table 1). An examination of these overlapping coding regions found motifs at locations appropriate to allow separation of the genes in most of the 95 cases. For example, the 3′-ends of KNB46045 and KNB46046 overlap by 37 bps (S1 Fig). Forty-four bps upstream of the annotated stop codon for KNB46045 is the conserved motif TGTTTGTT, which, if used to direct the generation of a new termination codon, would eliminate the overlap between the 2 genes.

Future studies of additional *Blastocystis* STs need to take polyadenylation-mediated stop codons into account. The mechanism has been demonstrated to be active in ST1 and ST7, suggesting that the mechanism evolved prior to the divergence of these 2 STs.

### Introns

**Fig 1:**
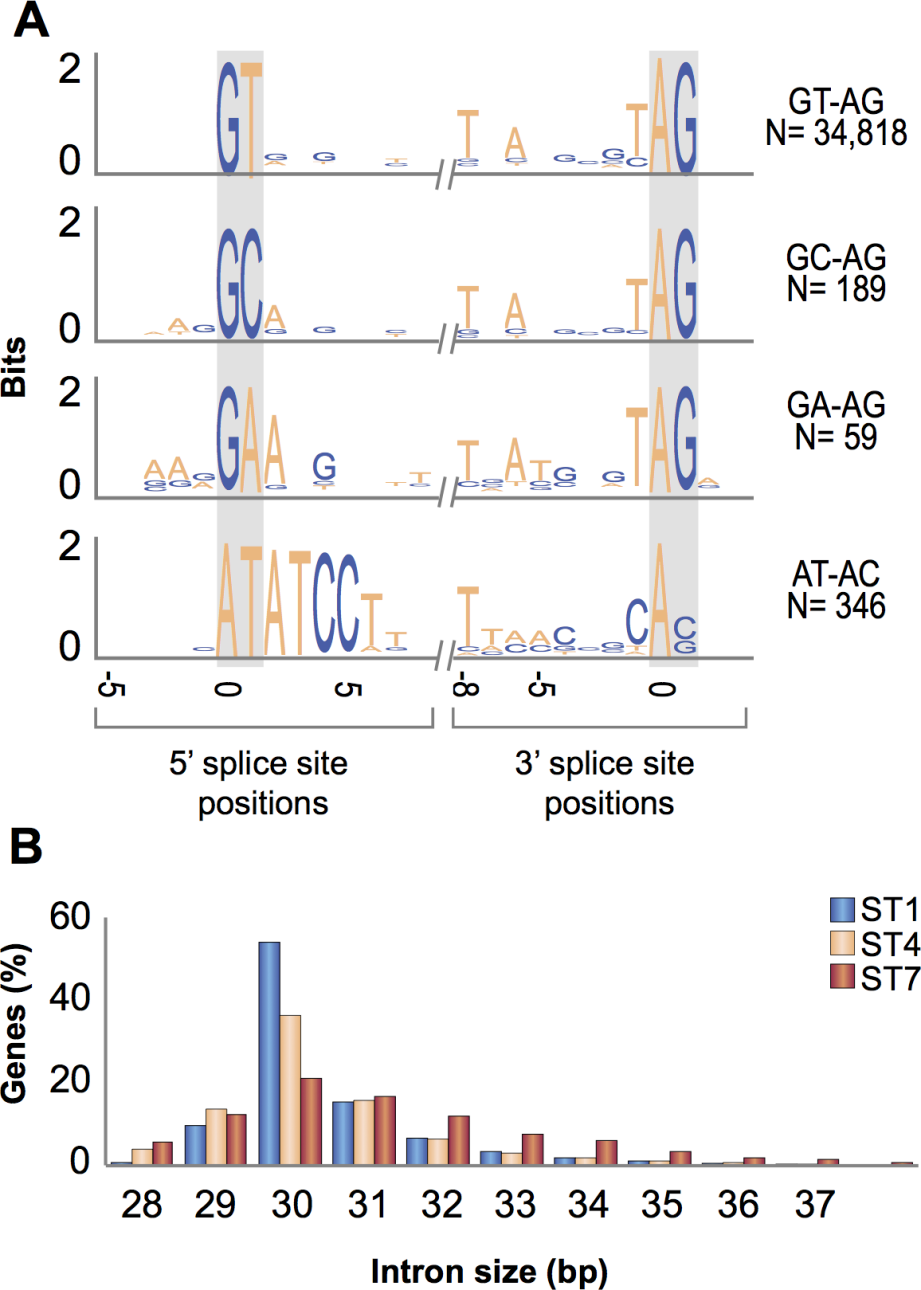
Spliceosomal introns in *Blastocystis*. (A) Sequences flanking the predicted exon–intron junctions in subtype (ST) 1 were aligned separately for each intron category and visualized with WebLogo3 (http://weblogo.threeplusone.com/). The category and number (*N*) of each spliceosomal intron type are shown on the right. (B) Distribution of intron size in 3 sequenced *Blastocystis* ST genomes. Data for this figure can be found in S11 Data.

The ST1 genome is substantially more intron rich than that of ST7 or ST4. It has 35,412 predicted introns compared with 18,200 in ST7 and 24,093 in ST4 (Table 1). Consequently, ST1 has the highest percentage of genes with introns and the highest number of introns per gene. The size of introns in ST1 is also less variable (Fig 1), with over half being 30 bps in length. In ST4 and ST7, the percentage of introns with a length of 30 bps is considerably lower. ST7 shows greater variation in intron size, with a much lower peak at 30 bps and higher percentages of introns in the size range 31–38 bps. ST1's relative intron richness may in part reflect methodological differences in gene calling. Exon/intron boundaries in ST1 were corrected using RNA-Seq transcriptome data, which were not available for ST4 and ST7. Depending on the parameters used to identify a "typical" gene and its exon/intron structure during the automated gene-calling process, having RNA-Seq data would tend to result in finding more real introns and annotating more introns with the correct boundaries.

Of the approximately 35,000 annotated introns in the *Blastocystis* ST1 nuclear genome, the vast majority (98.3%) represent the standard (or U2 type) spliceosomal introns characterized by GT-AG boundaries and spliced by the so-called major (U2) spliceosome containing U1, U2, U4, U5, and U6 small nuclear RNAs (snRNAs) containing small nuclear ribonucleo proteins. Two additional, much less abundant intron categories are also apparently spliced by the major spliceosome. One has GC instead of GT as the 5′ intron boundary and constitutes about 0.5% of all introns in both ST1 and ST7 (Fig 1), which is on par with the proportion of this intron found in metazoan or plant genomes [26]. The second category, characterized by the GA dinucleotide at the 5′ border, is even more sparse, with 59 such introns (i.e., only around 0.16% of all introns) identified in ST1 and only 9 supported by EST data in ST7. The existence of such introns is not without precedent, as such GA-AG introns were previously identified in the genome of the dinoflagellate *Symbiodinium minutum* [27].

The final intron category in *Blastocystis* corresponds to the minor, or U12-type, introns, characterized by boundaries typically exhibiting the dinucleotides AT-AC (Fig 1) and spliced by the minor (U12) spliceosome containing U11, U12, U4atac, and U6atac snRNA molecules. A systematic analysis of the *Blastocystis* ST1 genome revealed 346 U12-type introns in 319 genes. In addition, genes for all 4 snRNAs and for all 7 protein subunits (20K, 25K, 31K, 35K, 48K, 59K, and 65K) specific to the U12 spliceosome were identified [28] (S1 Table). U12-type introns and U12 spliceosome-specific components were not noted in the report on the ST7 genome [21], but both can be identified in the genome sequence of that ST (Fig 1, S1 Table). Hence, *Blastocystis* represents a previously missed eukaryotic lineage that has retained U12-type introns and the associated splicing machinery. In stramenopiles, only oomycetes have so far been known to exhibit both types of spliceosomal introns, whereas only the major “standard” type has been retained in the other lineages [29].

### Significant differences in gene complement between STs

**Fig 2:**
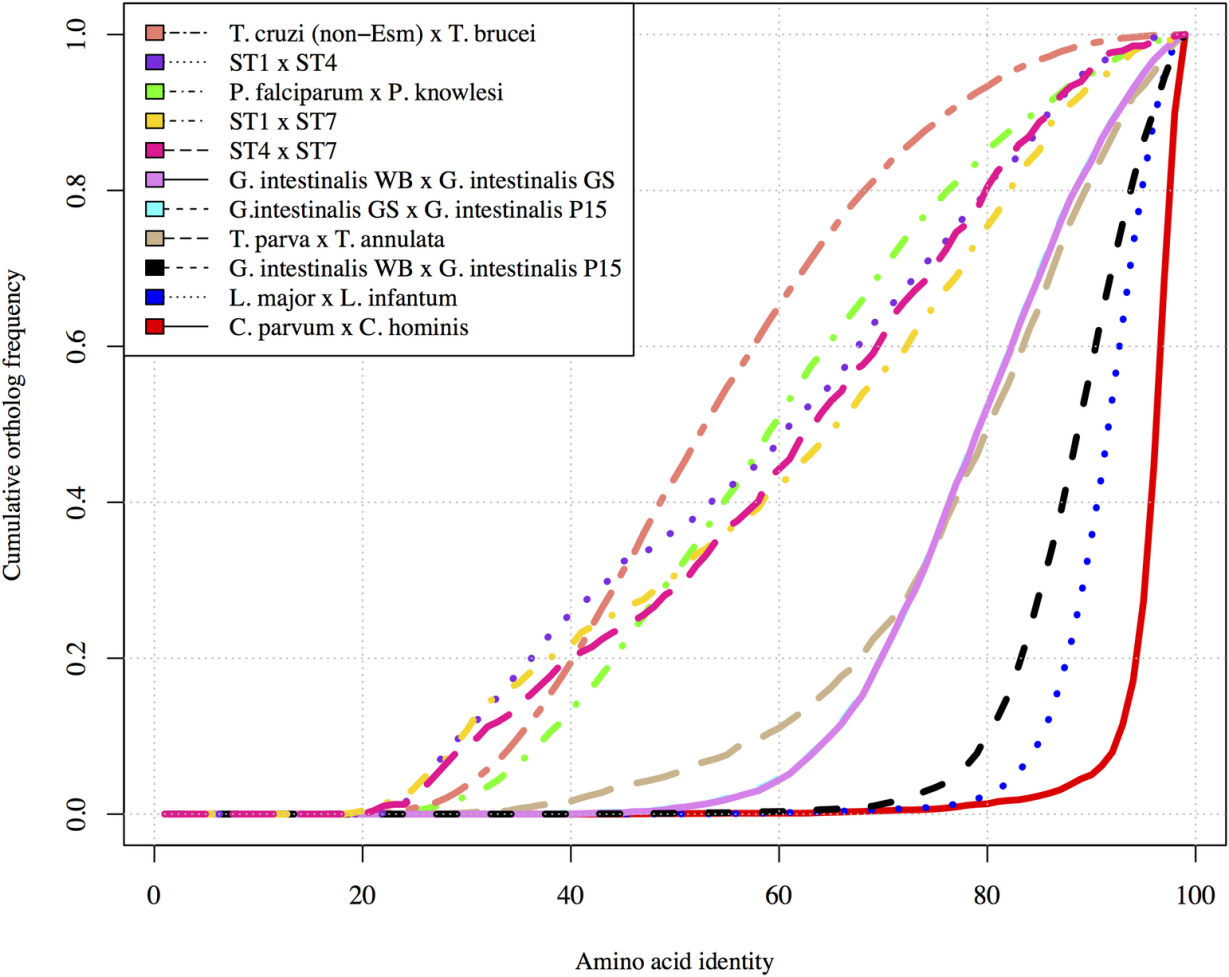
A comparison of amino acid identities between *Blastocystis* subtypes (STs) to protistan pathogens. Cumulative ortholog frequency is plotted on the y-axis while the x-axis represents the amino acid identity between orthologs. The more similar species pairs are to each other, the steeper the curve and further to the right. Note that the order of the pairs in the legend matches the order in the figure. Comparisons for *Blastocystis* STs were based on aligned regions of reciprocal best Basic Local Alignment Search Tool (BLAST) hits, while non-*Blastocystis* data were obtained from the authors of a comparative study of *Giardia intestinalis* [23]. Data for this figure can be found in S12 Data.

*Blastocystis* ST1 was found to have 6,544 protein-coding genes, in contrast to 6,020 in ST7 and 5,713 in ST4 (Table 1). An examination of the amino acid identities between putative orthologs reveals an extraordinary degree of dissimilarity among the *Blastocystis* STs (Fig 2). The median sequence identity of aligned regions of orthologs among STs 1, 4, and 7 ranges from 59% to 61% (S2 Table). The differences among the 3 STs exceed those observed for orthologs from pairs of species from parasitic protistan genera such as *Cryptosporidium* (*C*. *parvum*–*C*. *hominis*, Alveolata), *Leishmania* (*L*. *major*–*L*. *infantum*, Excavata), and *Theileria* (*T*. *parva*–*T*. *annulata*, Alveolata) (Fig 2) and indeed among *Giardia* strains (WB, GS, P15; Excavata) (Fig 2, S2 Table). The dissimilarity is comparable to that between species of *Plasmodium* (*P*. *falciparum*–*P*. *knowlesi*, Alveolata) and *Trypanosoma* (*T*. *cruzi*–*T*. *brucei*, Excavata). The extent of the dissimilarity among the *Blastocystis* STs supports the contention that they should be considered at least equivalent to separate species, particularly when placed in the context of other protistan pathogens. The lack of morphologically distinguishing traits and low correlation between ST and host [30] will continue, however, to make the taxonomy of *Blastocystis* challenging.

**Fig 3:**
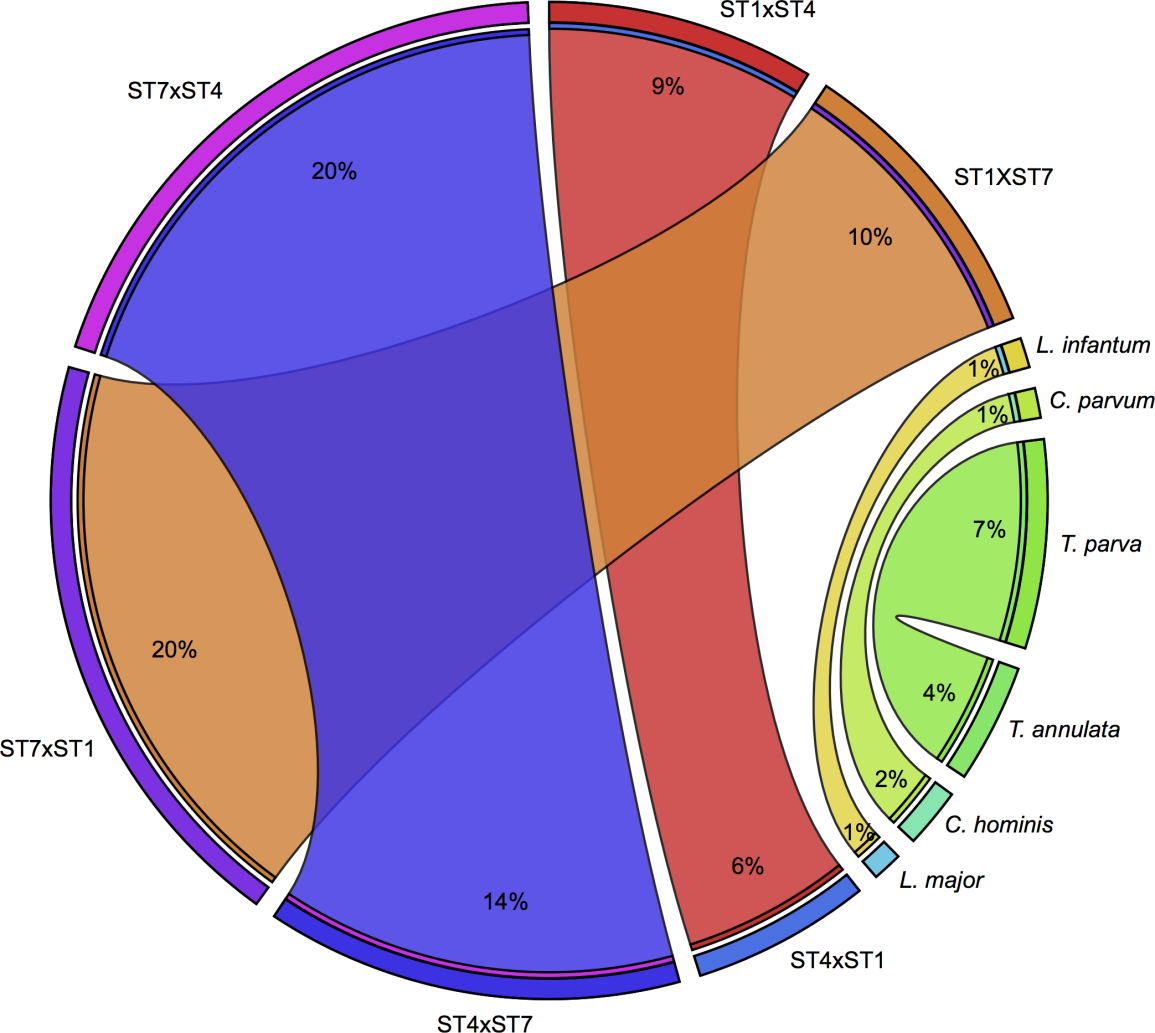
A comparison of unique genes between *Blastocystis* subtype (ST) pairs to pairs of protistan pathogens. The percentage of an organism’s protein-coding gene set, which is unique when compared to another organism’s protein-coding gene set and vice versa, is denoted by the width of the ribbon between the 2 as well as being indicated on the ribbon. For example, in a comparison between ST7 and ST1, 20% of the genes in ST7 are not represented in the ST1 set, while 10% of ST1's genes are not found in ST7. Comparisons are based on BLASTp results with an expect value (e-value) threshold of 1e-30 and >50% coverage of the query. **Abbreviations:** *C*., *Cryptosporidium*; *L*., *Leishmania*; *T*., *Theileria*. Plots were generated using Circos.

ST1 and ST4 genes are the least alike at the protein level while ST7 protein-coding sequences are usually more similar to ST1 than to ST4 genes (Fig 2). This is in line with recent phylogenetic analyses [5,20,31] that place ST4 in a clade sister to one that includes ST1 and ST7. There are also marked differences in gene content between STs. The percentage of genes present in ST1 but not in ST4 is significantly lower than the percentage of genes in ST1 not present in ST7 (Fig 3). Similarly, ST4 has far fewer unique genes when compared with ST1 than with ST7. The percentage of unique genes in ST7 is virtually identical in comparisons with ST1 and ST4. The differences in gene complement between the *Blastocystis* STs greatly exceed the differences between selected parasitic protistan species pairs (Fig 3). Beyond a common core of genes presumably devoted to common housekeeping tasks, the *Blastocystis* STs possess a substantial number of genes unique to each ST. The consequences of these major genetic differences between the STs are discussed below.

### Kinome

**Fig 4:**
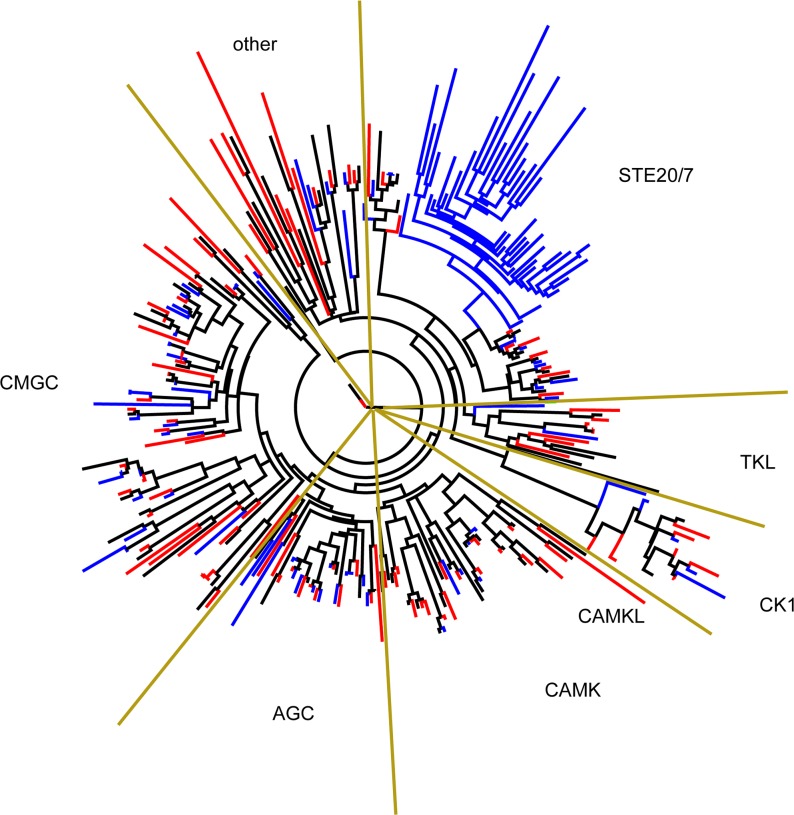
Randomized Axelerated Maximum Likelihood (RAxML) tree of kinases identified from *Blastocystis* subtypes (STs) 1, 4, and 7. Blue lines correspond to proteins from ST1, black from ST4, and red from ST7. Kinase families are indicated on the periphery.

Kinase enzymes modify target proteins via phosphorylation and participate in the regulation of cellular pathways, particularly those involved in signal transduction [32]. Our analysis of *Blastocystis* ST1 identified 221 kinases, which were classified according to kinase.com [33]. Representatives of most kinase groups are similarly distributed among STs 1, 4, and 7 (Fig 4) except for 2 clades showing lineage-specific gene expansion. The first is a small clade of calcium/calmodulin-dependent-like (CAMKL) kinases almost exclusive to STs 4 and 7 (Fig 4). The second and more striking example of ST differences is a clade of STE20/7 kinases. While ST4 and ST7 both encode representatives of the STE family, they appear to completely lack members of a cluster of 58 closely related STE20/7 kinases found only in the ST1 genome. This seems to be a spectacular case of gene family expansion specific to ST1.

The exact roles of the various kinases in *Blastocystis* STs and, in particular, the large group of STE20/7 kinases exclusive to ST1 are currently unclear. The STE20/7 family includes members of the mitogen-activated protein kinase (MAPK) signaling pathway, which regulates responses to extracellular stimuli [34]. The ST1 cluster does not exhibit a particularly close relationship with any specific gene of the STE20/7 family. In *Giardia*, 2 members of the MAPK family have been implicated in initiation of encystation [35], while in *T*. *brucei*, a MAPK is involved in mediating its interferon-γ-induced proliferation in the host [36]. MAPK pathways have been characterized, at least in pathogenic fungi, as a "functional nervous system" that controls virulence and modulates the outcome of the disease [37]. General “housekeeping” responses to stimuli would presumably be similar across the STs, so the highly developed exclusive MAPK pathway in ST1 must have some unique consequences and could be worthy of future functional studies.

### Secretome and degradome

**Fig 5:**
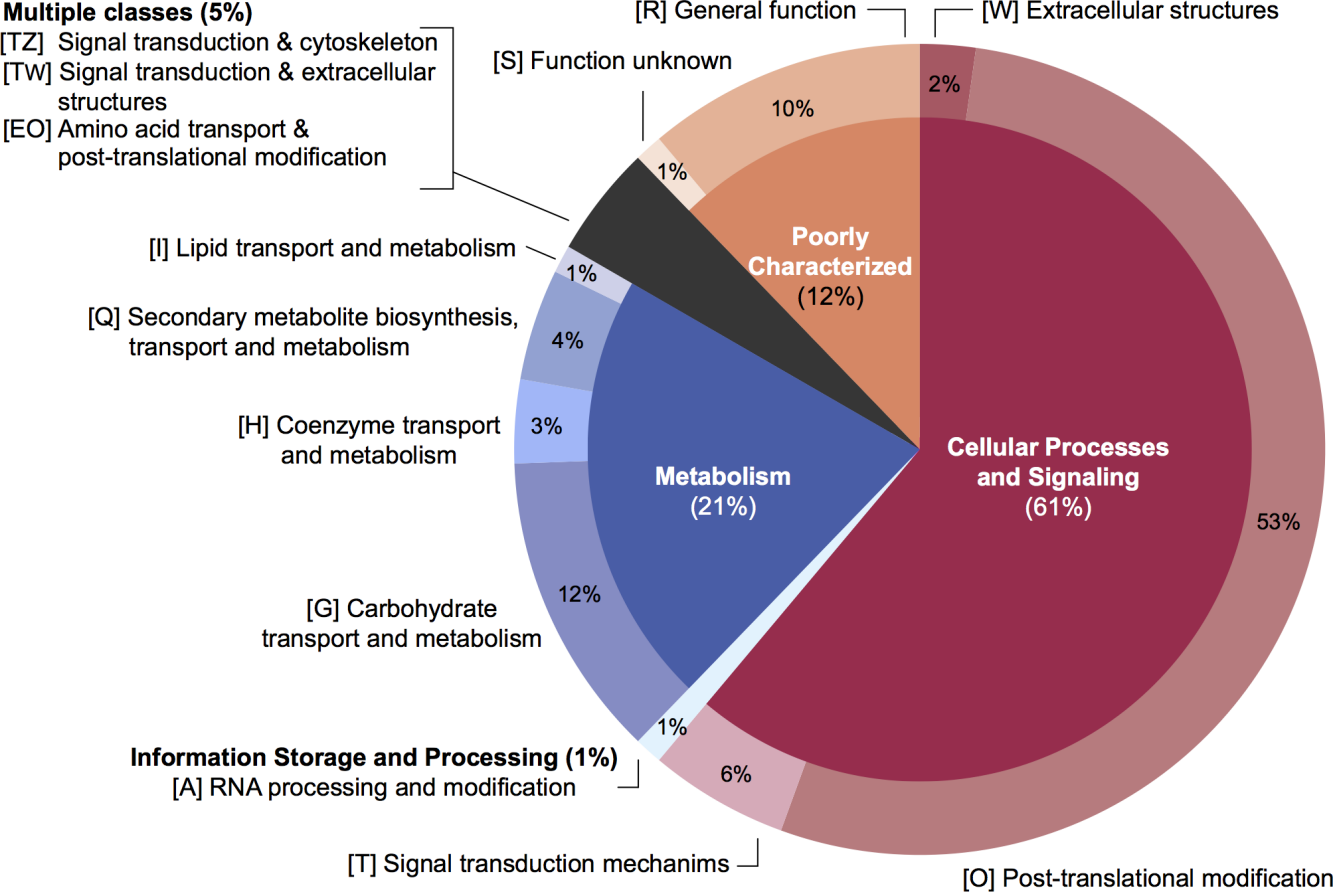
Eukaryotic orthologous groups (KOG) classification of putative *Blastocystis* subtype (ST) 1 secreted proteins. Eighty-nine proteins were predicted as secreted based on predicted signal peptide, extracellular localization prediction, and the absence of transmembrane regions. KOG categories were assigned using the Conserved Domain Database online tool. Percentage of secretome proteins of higher-order (inner circle) and lower-order (outer circle) KOG categories are shown. Single letter code(s) for each category are shown in square brackets. Data for this figure can be found in S1 Data.

A stringent, 4-step approach identified a total of 89 genes confidently predicted to encode secreted proteins in *Blastocystis* ST1 (S1 Data). The corresponding predicted number for ST7 was 307 [21], although those results were based only on SignalP predictions, a less stringent approach than the one used here. Most of the 89 secreted proteins predicted in ST1 are also present in ST7, with the exception of a metallophosphoesterase and GH2 and GH33 glycosyl hydrolases. Several of the 89 genes were not predicted as secreted in ST7 because their signal peptides were not part of the predicted gene models. More than half of these putative secreted proteins have a predicted role in posttranslational modification and protein turnover (Fig 5). We compared the secretory signal peptides of the different STs and were unable to identify a unifying characteristic apart from a core of 10 hydrophobic amino acids (S2 Fig). The majority of the secreted protein-coding genes in the 3 STs were present in more than 1 copy, with a notable expansion of cysteine proteases (see below).

Intriguingly, ST1 has 4 genes that are predicted to code for secreted collagen-like proteins. These 4 genes are very different at the amino acid level, with pairwise percentage identities only in the mid to upper 30s, but all have signal peptides that suggest they are secreted and all have the distinctive collagen-like motif GXY (glycine, second and third residues can be anything but frequently proline and hydroxyproline) repeated 63 times. ST4 also has 4 copies of genes encoding these collagen-like proteins, with only 1 predicted to be secreted, whereas ST7 has a single gene encoding a related protein that lacks a signal peptide. Based on sequence similarity and repeat characteristics, the *Blastocystis* collagen-like proteins are of the bacterial type [38]. A number of bacterial pathogens have collagen-like proteins that are involved in pathogenicity, immune response elicitation, and host–parasite interactions. For example, collagen-like proteins are able to bind to the human extracellular matrix, thereby aiding adhesion and colonization [39–41]. It is possible that collagen is one of the factors mediating adhesion of *Blastocystis* to enterocytes. Another possibility is that collagen may be part of a mechanism used by *Blastocystis* to trap bacteria and other microbial eukaryotes for nutritional purposes. This process has been observed by electron microscopy, although an exact mechanism has not been described [42–44]. At present, the roles played by collagen-like proteins in *Blastocystis* are purely speculative, based on their similarities to bacterial proteins that are clearly involved in pathogenicity. However, it suggests fertile ground for further investigation, specifically into whether differences in both number of genes and potential for secretion are implicated in variable virulence.

Another class of proteins demonstrating clear ST differences is proteases or peptidases. Proteases are crucial for many biological processes and constitute potential virulence factors in parasitic protists [45]. Cathepsin B, a cysteine protease, has been linked to increased intestinal cell permeability [8], while other cysteine proteases have been reported to cleave human secretory IgA [46] and induce up-regulation of interleukin 8 cytokine transcription and secretion [47] in intestinal epithelial cells.

**Fig 6:**
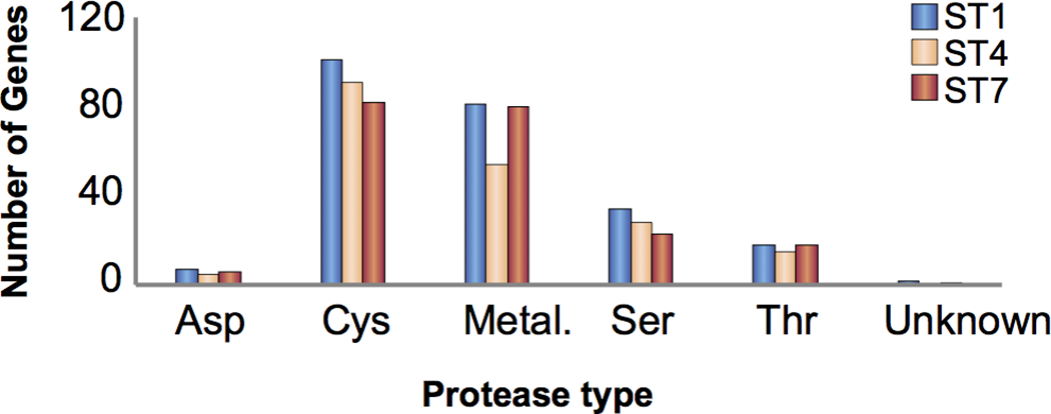
Proteases by type in *Blastocystis* subtype (ST) 1, ST4, and ST7. **Abbreviations:** Asp, aspartic; Cys, cysteine; Metal., metallo; Ser, serine; Thr, threonine. Data for this figure can be found in S2 Data.

*Blastocystis* has a large number of proteases, with the 3 STs having mostly similar profiles, but with some notable differences. The total number of proteases encoded in ST1 is 243, with 198 in ST4 and 210 in ST7 (S2 Data) (Fig 6). All 3 STs encode aspartic, cysteine, metallo, serine, and threonine proteases. The most prevalent protease genes are those of the cysteine type, constituting between 39% (ST7) and 47% (ST4) of the degradome. Undoubtedly, many cysteine proteases play roles that are conserved in eukaryotes, such as lysosomal function, autophagy, and ubiquitination, but others have been implicated in host–parasite interactions [48–50]. All 3 STs show extensive gene expansions of the cysteine protease families C1, C13, and C19. A large number of C1 genes is fairly common. Less common is the number of C13 genes found in ST1 (16), ST4 (11), and ST7 (11). Most protist genomes contain fewer than 5 C13 genes and many just have a single version (see MEROPS database, merops.sanger.ac.uk, [51]). The only protist known to approach the number of C13 genes seen in *Blastocystis* is the sexually transmitted human excavate *Trichomonas vaginalis* (10), in which at least 1 type of C13 protein has been implicated in trichomonal cytoadherence [52]. While *Blastocystis* appears to have an elevated number of C19 genes compared with other protists (MEROPS database), currently there is no indication that these genes are involved in anything other than standard intracellular removal of ubiquitin molecules.

Other protease families that exhibit large differences between STs are C56, C95, C97, and M16 (S2 Data). The biological significance, if any, of these differences is currently unknown. The most striking disparity between the STs is seen in metallo-type proteases (Fig 6, S2 Data). The divergence in the number of genes belonging to this type is entirely attributable to the complete absence of subfamily M23B genes in the ST4 genome, while both ST1 and ST7 have 29 members. The M23 family is composed of metallopeptidases involved in the lysing of peptidoglycans in bacterial cell walls for either defense or feeding (MEROPS database). What functions they possess in *Blastocystis* and why they are absent from ST4 are open questions. Differences in the number of the S54 rhomboid serine proteases were also identified, with ST1 having 4 genes, ST4 having 3, and ST7 having only 1. These transmembrane peptidases play a role in the invasion of host cells in the alveolates *Toxoplasma*, *Cryptosporidium*, and *Plasmodium*, while in the amoebozoan *Entamoeba histolytica*, the single rhomboid protease is vital to immune evasion via the cleavage of lectins during receptor capping [53–55].

### Unusual aspects of *Blastocystis* ST1

#### Mitochondria

The mitochondria of anaerobic protists are often metabolically distinct from their aerobic relatives [56], as they typically couple ATP generation to the production of hydrogen and not oxidative phosphorylation. Due to the lack of canonical features, they are frequently referred to as MROs. The first EST survey of *Blastocystis* ST1 revealed the MRO to have elements of canonical mitochondria, as exemplified by a highly reduced cytochrome-mediated electron transport chain with only complexes I and II being present. These organelles also showed features characteristic of hydrogenosomes, including MRO-targeted homologs of [FeFe] hydrogenase and pyruvate:ferredoxin oxidoreductase (PFO) [18]. This mosaic composition of the MRO proteome has intrigued evolutionary biologists, as it represents an intermediate form between classical mitochondria and hydrogenosomes. Comparative analyses of MRO genomes of 8 STs have shown that they are identical in gene content and gene order [17,19,20].

**Fig 7:**
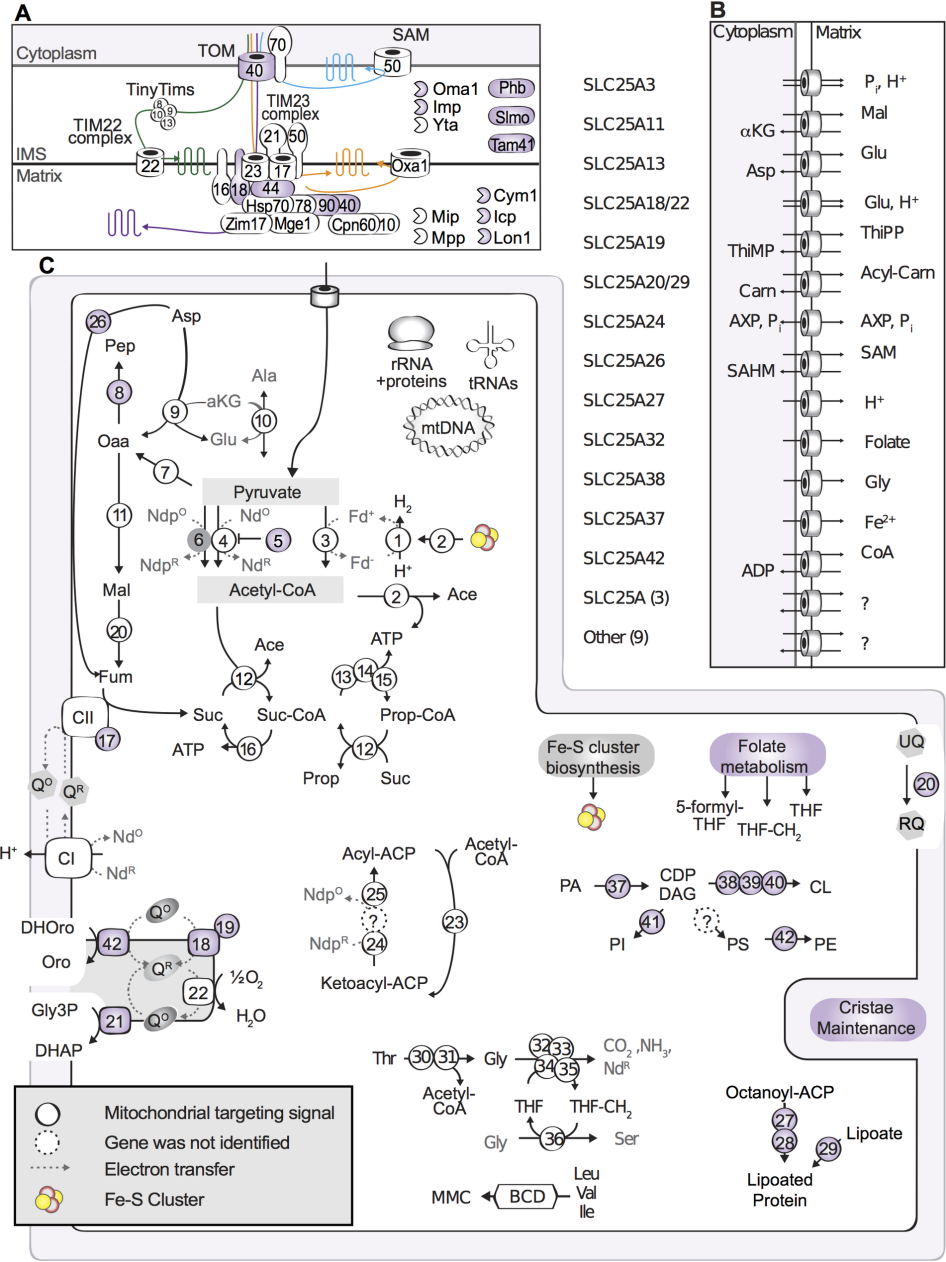
Select metabolism of *Blastocystis* mitochondrion-related organelles (MROs). Proteins that were not reported in previous studies are shaded in purple. (A) Mitochondrial protein import and folding apparatus. Colored lines represent proteins destined for the outer mitochondrial membrane (blue), inner mitochondrial membrane (green and orange), and mitochondrial matrix (purple). Numbers correspond to the protein number in the complex (e.g., TOM40). Proteases are depicted with partial circles. (B) Mitochondrial carriers from solute carrier protein (SLC) family 25A and their predicted cargo. (C) Metabolic features highlighting the MRO’s role in energy generation and amino acid and lipid metabolism. Protein descriptions are outlined in S3 Data. Standard amino acid abbreviations are used: Ace, acetate; ACP, acyl carrier protein; aKG, alpha-ketoglutarate; BCD, branched chain amino acid degradation; Carn, Carnitine; CDP-DAG, cytidine diphosphate diacylglycerol; CL, cardiolipin; DHAP, dihydroxyacetone phosphate; DHOro, dihydroorotate; Fd, Ferredoxin; Fum, fumarate; Gly3P, glycerol-3-phosphate; Mal, malate; MMC, methyl-malonyl-CoA; Nd(p), NAD(P); Oaa, oxaloacetate; Oro, orotate; PA, phosphatidic acid; PE phosphatidylethanolamine; Pep, phosphoenol pyruvate; PI, phosphatidylinositol; Prop, propionate; PS, phosphatidylserine; Q^O/R^, quinone/quinol, oxidized or reduced; RQ, rhodoquinone; SAHC, S-adenosylhomocysteine; SAM, S-adenosylmethionine; Suc, succinate; THF, tetrahydrafolate; ThiMP, thiamine monophosphate; ThiPP, thiamine pyrophosphate; UQ, ubiquinone. Data for this figure can be found in S3 Data.

From the *Blastocystis* ST1 nuclear genome sequence, a variety of MRO proteins were identified that were previously unreported (Fig 7, S3 Data). In particular, additional components of the protein import apparatus, including transporters, chaperones, and proteases, were identified (Fig 7). Genes were also found that are involved in “typical” mitochondrial processes such as cardiolipin and phospholipid metabolism, electron transfer (Complex II assembly [SDH5], electron transferring flavoprotein), Fe-S cluster biogenesis (NFU1), cofactor/vitamin metabolism (folate, B5, B12, steroid, and lipoate), fatty acid biosynthesis, cristae maintenance (Mic60), and regulation (PDH kinase). Genes encoding MRO proteins that are only found in anaerobes were identified, such as the rhodoquinone biosynthesis enzyme RQUA and a putative ATP-generating acetyl-CoA synthetase (ACS).

Previous attempts to identify PFO and hydrogenase activity in the MRO of *Blastocystis* ST1 have been unsuccessful [57]. While this observation might be due to the labile nature of these enzymes, it is possible that additional factors are at play. PFO catalyzes the oxidation of pyruvate and generates reduced ferredoxin to be used by hydrogenase to generate hydrogen. PNO is homologous to PFO, but contains a C-terminal NADPH-hemoprotein reductase domain [58]. PNO (and not PFO) activity has been reported previously in *Blastocystis* [57]; however, while several candidate PNO proteins could be identified, none had a predicted targeting peptide. It is possible that at least 1 of these possesses a cryptic mitochondrial targeting signal; such signals are not so uncommon in model system mitochondria [59]. However, genes encoding 3 homologs of PFO and a standalone NADPH-hemoprotein reductase were identified, all of which had canonical targeting peptides for localization to MROs. One possible scenario is that these mitochondrial PFO proteins are functioning *in trans* together with the soluble NADPH-hemoprotein reductase as PNOs, thus explaining the lack of organellar PFO activity. A similar situation occurs for the Fe hydrogenase homologs of *Blastocystis*. Two of the hydrogenase genes identified in ST1 have clear mitochondrial targeting peptides but possess an additional electron-transferring domain (flavodoxin), while the lone canonical hydrogenase (i.e., without the flavodoxin domain) is cytosolic. Recent reports have identified these atypical hydrogenases in other anaerobic protists [59–61]. Moreover, ST1 does not encode the maturases HydF and HydG, which are typically necessary for the correct assembly of the mature hydrogenase enzyme. A possible scenario is that the *Blastocystis* ST1 hydrogenase does not participate in hydrogen production but instead uses an alternative electron acceptor to protons. While these scenarios are speculative, the noncanonical domain architecture of the *Blastocystis* hydrogenases and PF(N)Os is intriguing and additional biochemical investigations will be needed to test these hypotheses.

**Fig 8:**
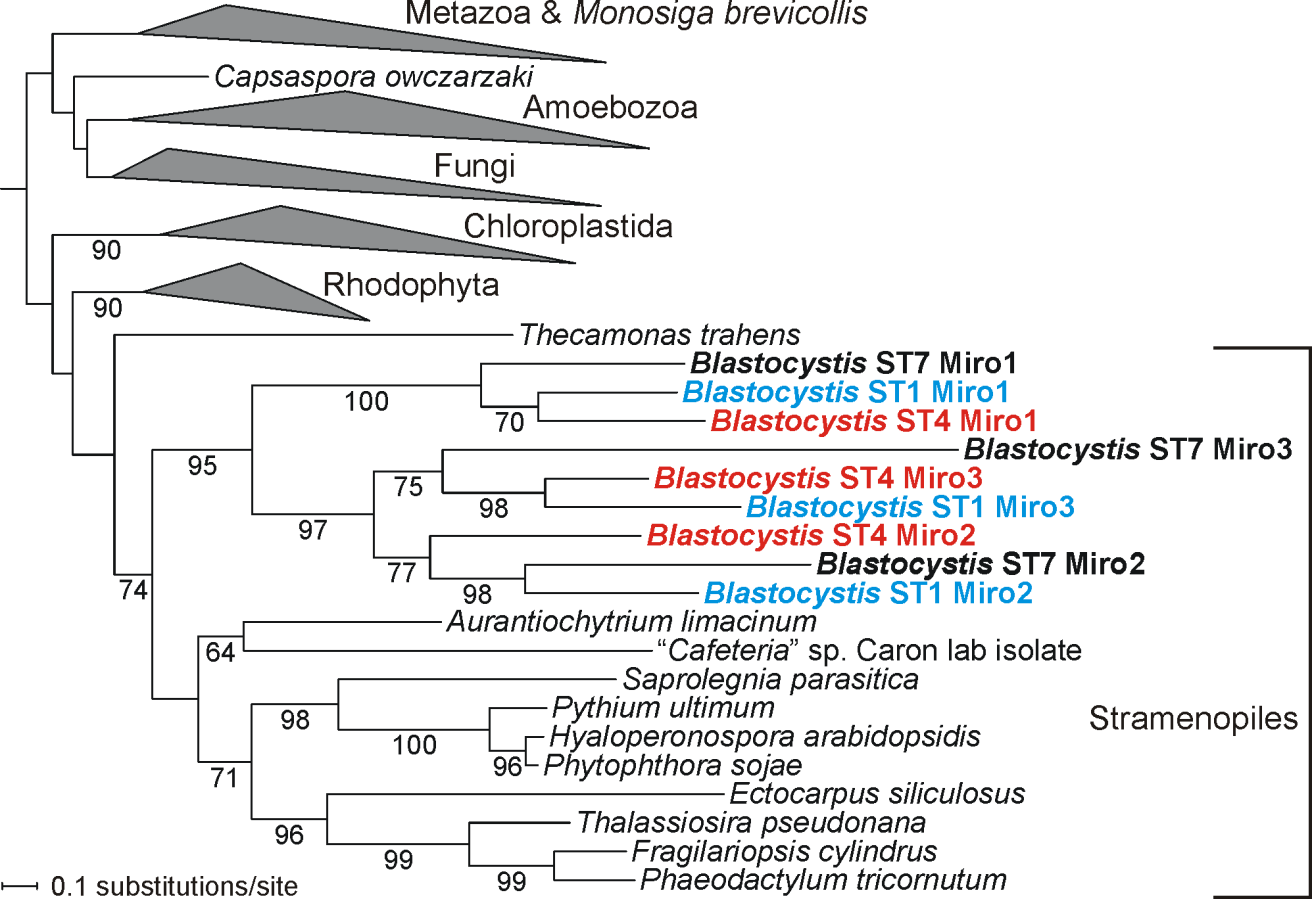
Maximum likelihood phylogenetic tree of Miro protein sequences. The tree was calculated using Randomized Axelerated Maximum Likelihood (RAxML). Bootstrap support values above 50% are shown at branches. For simplicity, clades comprising sequences from major monophyletic groups of eukaryotes were collapsed, displaying in detail only the clade of sequences from stramenopiles. Note the 3 Miro paralogs that have apparently emerged from *Blastocystis*-specific gene duplications before the divergence of the ST1, ST4, and ST7 lineages (gene identifiers of the *Blastocystis* Miro sequences are provided in S4 Data).

An analysis of *Blastocystis* GTPases encoded in STs 1, 4, and 7 (S4 Data) identified Miro, a protein that has been implicated in regulating mitochondrial morphology, intracellular transport, and interactions with the endoplasmic reticulum (ER). Miro is a multidomain protein composed of 2 divergent GTPase domains separated by a region that includes 2 Ca^2+^-binding EF-hand motifs. The distal portion of the C-terminus includes a transmembrane domain anchoring the Miro protein in the outer mitochondrial membrane [62]. It is widely conserved across eukaryotes including stramenopiles but was previously found to be missing from all eukaryotic lineages with anaerobic MROs (hydrogenosomes or mitosomes), including Microsporidia, *Entamoeba*, and Metamonada [63]. The presence of Miro in *Blastocystis* is thus somewhat unexpected, but in fact further underscores the intermediate position of the MRO organelle in this organism between canonical aerobic mitochondria and highly modified mitochondrial derivatives such as hydrogenosomes and mitosomes. More unexpected is the presence of 3 quite different Miro paralogs conserved across all 3 *Blastocystis* STs, apparently resulting from duplications specific to the *Blastocystis* lineage (Fig 8). Duplicated Miro genes were previously detected only in eukaryotic groups known to have experienced cycles of whole-genome duplications, such as vertebrates, some plants, and some ciliates [63], so the expanded family of Miro genes in *Blastocysti*s is noteworthy.

#### Peroxisomes

**Fig 9:**
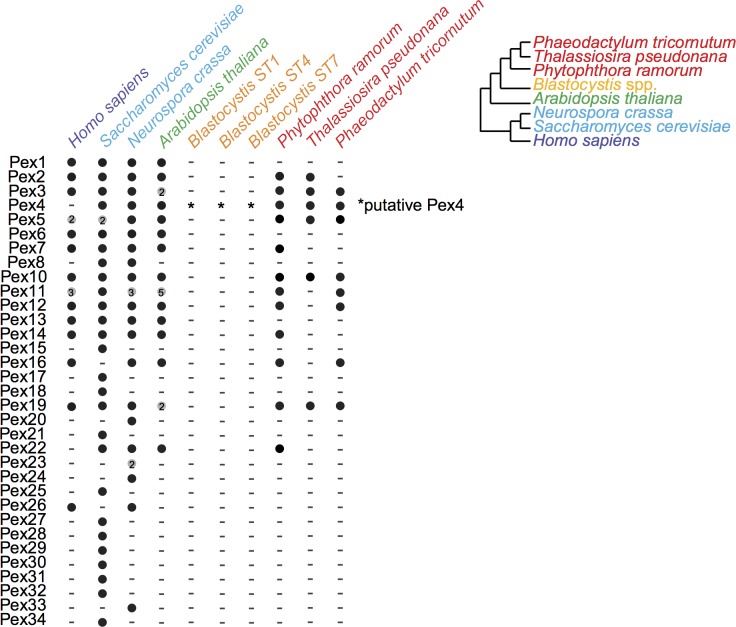
Peroxisomal machinery genes in selected organisms. Black circles indicate the gene is present. Grey circles indicate multiple isoforms.

In contrast to stramenopiles with canonical peroxisome [64], *Blastocystis* STs 1,4, and 7 appear to have retained only a single subunit of the Dsl1 multisubunit tethering complex (S3 Fig), which is known to be involved in transport between the ER and the peroxisome [65]. Reduction of this complex has been observed in other eukaryotic organisms thought to lack peroxisomes [64]. Characterization of peroxisomes in genomes from the SAR clade is limited (reviewed in [66]), but there is ultrastructural and/or biochemical evidence for peroxisomes in *Phytophthora palmivora* [67] and *Phaeodactylum tricornutum* [68], and peroxisomes have been predicted to be present in *T*. *pseudonana* [69]. Like nearly all other parasitic organisms [70], peroxisomes have not been described ultrastructurally for *Blastocystis*. Consistent with this, with the Dsl1 result, and with a report that failed to find the AAA-ATPases Pex1 and Pex6 [71], no *PEX* genes (responsible for peroxisome biogenesis) were identified in any of the *Blastocystis* STs, with the exception of a possible Pex4p homolog (Fig 9). This makes *Blastocystis* only the second example, after alveolates, of a lineage with MROs retaining aspects of classical mitochondria but lacking peroxisomes [70].

### Membrane-trafficking machinery

The membrane-trafficking system enables transport of proteins and lipids between intracellular locations and is an interface with the extracellular environment. Crucial to the healthy working of eukaryotic cells, it underpins the pathogenic mechanisms of diverse eukaryotic parasites through the release of virulence factors as well as the uptake of metabolites from the host. Trafficking is enabled by a suite of proteins involved in vesicle formation and fusion [72]. Comparative genomic and molecular phylogenetic analyses have established that a relatively complex complement of membrane-trafficking machinery was present in the last eukaryotic common ancestor (LECA) [73].

Our analyses showed that homologs of nearly all components of the vesicle-formation and -fusion machinery present in the LECA are also encoded in *Blastocystis* STs 1, 4, and 7 (S3 Fig). Indeed, there is evidence for somewhat expanded paralog numbers in several of the complexes, most notably in the Sec24 subunit of the COPII coat; the Arf GAPs; the HOPS and GARP tethering complexes; and the adaptor protein complexes 1, 2, and 4 (S3 Fig). This is also where much of the variability between the STs is observed (along with the numbers of some endosomal sorting complexes required for transport [ESCRT] paralogs).

A number of Ras superfamily proteins (or small GTPases) known to be involved in membrane and protein trafficking have orthologs in *Blastocystis* (S4 Data). Notable among them are 2 (ST7) or 3 (ST4, ST1) paralogs of a GTPase that serves as the membrane-anchored β subunit (SRβ) of the signal-recognition particle receptor on the ER that is involved in the cotranslational import of proteins into the ER [74]. Most eukaryotes, including other stramenopiles, employ only 1 SRβ protein, so the functional significance of the varying number of SRβ paralogs in *Blastocystis* is unclear.

*Blastocystis* STs also harbor some Ras superfamily members that appear to have emerged in this lineage through extensive divergence of existing or duplicated genes. These can be considered “novel” genes and may potentially underpin significant evolutionary innovations. For instance, ST1 and ST4 both encode a divergent Rab7-like paralog (Rab7L in S4 Data) that is apparently missing from the genome of ST7. Although the cellular function is difficult to predict for such divergent paralogs, the similarity of Rab7L to standard Rab7 proteins suggests that it may be involved in some trafficking processes associated with lysosomes or vacuoles. The *Blastocystis* genomes encode 5 additional divergent Rab-related proteins whose evolutionary origin cannot be deduced with confidence and hence are labeled RabX1 to RabX5 (S4 Data). These proteins are presumably involved in specialized membrane-trafficking pathways in *Blastocystis*, but their functions cannot be predicted from sequence analyses alone. The 2 Rab1 paralogs in *Blastocystis*, annotated as Rab1A and Rab1B, differ from the previous examples in that their origin is more ancient, as they apparently stem from a Rab1 duplication previously suggested to be an evolutionary novelty of the SAR supergroup [75]. The functional significance of the 2 differentiated Rab1 paralogs in organisms of the SAR clade remains unknown despite an attempt to characterize both paralogs in *Toxoplasma gondii* cells [76]. Most recently, Rab1A was found in association with rhoptries in schizonts of *P*. *falciparum* and suggested to be involved in regulating vesicular trafficking from the ER to the former secretory organelles of the parasite [77].

### Anaphase promoting complex/cyclosome

Control of the cell cycle and cell proliferation is mediated extensively by the anaphase promoting complex/cyclosome (APC/C) [78]. It functions as an E3 ubiquitin ligase that coordinates the degradation of specific substrates via the 26S proteasome at specific points in the cell cycle [79]. Typically, the complex is composed of about a dozen subunits with a combined mass of about 1.5 MDa. It can be divided into 3 functional parts or subcomplexes [80]: (i) a structural complex serving as a scaffold, (ii) a catalytic arm, and (iii) a tetratricopeptide repeat (TPR) arm designed to position the substrate for successful transfer of ubiquitin. Surprisingly, *Blastocystis* seems to lack genes for all the subunits that make up the scaffold (i.e., APC1, APC4, and APC5), and to encode a reduced TPR arm composed of only 2 subunits versus typically 4 in other stramenopiles and up to 7 in other eukaryotes [78]. Particularly notable is the loss of the APC3 subunit, which is found in virtually all eukaryotic organisms. However, *Blastocystis* appears to be unusual in that it has several paralogs encoding the TPR subunit APC8, which potentially may compensate for the absence of the other subunits in the TPR arm (S5 Data).

The APC/C interacts with a number of adaptors and coactivators that modulate its activity and specificity. The most important of these adaptors are cell-division cycle protein 20 (Cdc20) and cadherin-1 (Cdh1) because, at various stages of the cell cycle, they are essential for activation of the complex and selecting which substrates to interact with [81]. In particular, APC/C-Cdh1 plays a role in DNA synthesis during the G1/S phase because it allows the 26S proteasome to degrade several DNA replication inhibitors [80]. Surprisingly, while the vast majority of eukaryotes possess both Cdc20 and Cdh1, *Blastocystis* only has a homolog of Cdc20. How the end of anaphase is regulated in *Blastocystis* remains an open question.

In addition, genes encoding 2 of the main APC/C targets that are involved in the integrity and regulation of the cohesion complex, Scc2/Scc4 and Eco1, were absent. This protein complex keeps sister chromatids together and regulates their separation during cell division. Scc2/Scc4 aids in loading the complex onto the chromosome, while Eco1 is responsible for the establishment of cohesion between cohesin and chromatin [80]. The absence of these components in *Blastocystis* and other stramenopiles suggests that an alternative route may exist to achieve a properly functioning cohesion complex and the separation of sister chromatids.

### DNA repair and meiosis

Potential homologs of proteins involved in DNA damage response and repair, chromatin structure relevant to repair, and meiosis, a process not previously attributed to the life cycle of *Blastocystis*, were identified (S4 Fig, S6 Data). Homologs of genes encoding 9 out of 11 meiosis-specific proteins required for the progression of meiosis in other organisms [82,83] are found in *Blastocystis* ST1 and ST4; these include Hop1, Spo11-2, Top6BL, Dmc1, Hop2, Mnd1, Msh4, Msh5, and Mer3. Msh5 is absent from ST7, and Rec8 and Spo11-1 were not identified in any of the STs. Similar to *T*. *vaginalis* [84], the *Blastocystis* genomes apparently do not encode components of the nonhomologous end-joining machinery, suggesting that homologous recombination is the principal mechanism for the repair of double-stranded DNA breaks.

### Carbohydrate active enzymes

A total of 203 carbohydrate active enzymes (CAZymes) were identified in the *Blastocystis* ST1 genome (S7 Data). The most interesting cases are discussed below and additional details are provided in S1 Text.

#### Metabolism of nonreducing disaccharides

Sucrose and trehalose are the nonreducing disaccharides present in eukaryotes. Sucrose metabolism is completely absent in stramenopiles including *Blastocystis*, as deduced from the absence of genes encoding sucrose synthase (family 4 of the glycosyltransferases [GT4]), sucrose-phosphate synthase (GT4), and invertases (glycoside hydrolase family 32 [GH32 and GH100]) in ST1 and ST7. OtsA-OtsB is the most widespread trehalose biosynthetic pathway, present in both eukaryotes and prokaryotes [85]. This pathway involves the synthesis of trehalose-6-phosphate by the GT20 trehalose phosphate synthetase (TPS). Two GT20 enzymes, similar to the typical stramenopile TPSs, are encoded in ST1 and ST7 (S7 Data), but no enzyme capable of degrading trehalose was identified. However, ST1 does encode several GT4 family members that are highly divergent, so it is possible that these proteins have trehalose-phosphorylase catabolic activity.

#### Storage polysaccharide metabolism

*Blastocystis* has a small but apparently complete set of glycogen metabolism genes: a glycogen synthase (GT5), 2 glycogen phosphorylases (GT35), branching enzyme (GH13), and indirect branching enzyme (GH133) (S7 Data). This is the first reported case of a stramenopile with α-glucan metabolism. In general, stramenopiles utilize β-glucans as storage polysaccharides and lack starch or glycogen metabolism-related genes, as evidenced by the stramenopile genomes examined so far (diatoms, brown algae, and oomycetes) [86,87]. Our results are consistent with previous microscopic observations of glycogen-like particles in the cytoplasm of several species of *Blastocystis* [88–90].

Interestingly, genes encoding enzymes involved in β-glucan metabolism are completely absent from all 3 genomes. This finding indicates that storage metabolism differs significantly between *Blastocystis* and other stramenopiles for which there are published analyses [86]. Other stramenopiles are β-glucan accumulators and lack α-glucan-related gene homologs. However, a recent search of data from the Marine Microbial Eukaryote Transcriptome Sequencing Project [91] for the basal branching stramenopile *Cafeteria* revealed strong matches for a glycogen synthase gene (S3 Table) as well as the absence of β-glucan-related genes. The presence of α-glucan metabolism in *Blastocystis* and *Cafeteria* can be accounted for by lateral gene transfer (LGT) or by maintenance of an ancestral eukaryotic glycogen metabolism lost in stramenopile sister lineages. Quite significantly, the glycogen synthase enzyme belongs to the GT5 database of carbohydrate-active enzymes (CAZy) family, meaning the animal hosts of *Blastocystis* can be rejected as possible LGT sources, as they all contain the very different GT3-type enzymes. Given that alveolates also employ α-glucan metabolism [86], it is likely that α-glucan metabolism was present in the ancestor of stramenopiles. The presence of the indirect debranching enzyme (iDBE), absent from red and green algae, suggests that this network is not a secondary endosymbiosis relic from Archaeplastida.

#### Coat carbohydrates

Previous spectrophotometric analyses have shown that the surface coat of *Blastocystis* contains α-D-mannose, α-D-glucose, N-acetyl-α-D-glucosamine, α-L-fucose, chitin, and sialic acid [92]. Our analyses have confirmed *Blastocystis* STs 1, 4, and 7 encode a eukaryotic-type chitin synthase (GT2) and 2 eukaryotic-type chitinases (GH18) orthologous to proteins found in other taxa, indicating that this organism is capable of synthesizing and degrading this polysaccharide. No orthologs for fucose and sialic acid synthesis were identified. Many pathogens have developed the ability to harvest sialic acid from their hosts and incorporate it into their own glycoconjugates. Sialylated glycoconjugates in many pathogens seem to be crucial for their survival in the mammalian host, possibly serving as molecular mimics of host cell surfaces to evade immune attack [93]. A bacterial-type α-fucosidase (GH29) ortholog was found in all 3 genomes. Two prokaryotic-type CAZymes were present in ST1 and ST4 but not in ST7: an exo-α-sialidase (GH33) and an α-fucosidase (GH95). All 3 bacterial-type enzymes are part of a suite of genes that have been acquired through LGT and are discussed in a separate analysis [94].

### Intermediary metabolism

Unlike the intestinal parasites *Giardia* and *Entamoeba*, which rely on the host as a source of purines and pyrimidines [95,96], *Blastocystis* ST1 possesses complete pathways for the de novo synthesis of these compounds (S8 Data). Its capacity for de novo amino acid biosynthesis is limited to alanine, aspartate, and glutamate, while serine and glutamine can be produced via conversion from other amino acids. *Blastocystis* ST1 has a mostly complete folate biosynthesis pathway, lacking only the alkaline phosphatase responsible for the dephosphorylation of 7,8-dihydroneopterin 3′-triphosphate. However, this enzyme is also lacking in many probiotic bacteria such as bifidobacterial, which appear to use another NUDIX enzyme [97]. While genes encoding homologs of this protein could not be identified, it seems likely that *Blastocystis* ST1 uses an uncharacterized alkaline phosphatase to complete the pathway.

Unlike most parasites, *Blastocystis* ST1 appears to have a nearly complete de novo thiamine (vitamin B1) biosynthesis pathway. In this pathway, 4-amino-2-methyl-5-hydroxymethylpyrimidine (HMP) is pyrophosphorylated by HMP kinase sequentially to form HMP-PP, which is in turn condensed with thiazole to form thiamine phosphate by thiamine-phosphate pyrophosphorylase. Only the thiazole salvage enzyme hydroxyethylthiazole kinase (ThiM) could be identified, suggesting thiamine biosynthesis in *Blastocystis* ST1 is either dependent on exogenous thiazole or synthesizes thiazole by an unknown mechanism. The latter is not unprecedented, as *P*. *falciparum* has been shown to synthesize vitamin B1 de novo despite having the same repertoire of thiamine synthesis-related genes as *Blastocystis* [98].

### Cytochrome P450 gene family

Cytochrome P450 (CYP) is a large and versatile heme-containing protein superfamily containing at least 317 families and numerous subfamilies (https://cyped.biocatnet.de/sequence-browser). These proteins are found in all domains of life and in all major eukaryotic lineages. However, no homologs were found of any of the CYP families in the 3 *Blastocystis* genomes. Genomes available for other stramenopiles do encode various CYPs. The diatom *P*. *tricornutum* has 3 CYP genes, consisting of 2 CYP97 and 1 CYP51 enzymes, whereas *T*. *pseudonana* encodes 9 family members [99]. *P*. *falciparum* was the first documented eukaryotic species without a CYP-encoding gene [100]. However, it should be noted that Kinetoplastida such as *T*. *brucei* have a limited set of CYPs devoted to sterol synthesis (e.g., CYP51A). Another apicomplexan parasite, *T*. *gondii*, has a single CYP gene, encoding a steroid 11-β hydroxylase. Since sterols are essential for eukaryotic membranes, the lack of CYP51 in *Blastocystis* suggests that it obtains sterols from its host, as does *Giardia* [101].

### Concluding remarks

The intestinal eukaryote *Blastocystis* continues to be of significant clinical interest because of it widespread prevalence. Despite years of study, however, its pathogenicity and role in the gut remain controversial. This ambiguity is compounded by the presence of STs that, while morphologically indistinguishable, are nevertheless genetically heterogeneous. The extent of the differences and the degree to which they matter clinically are still unclear, but the increase in genomic data reported here opens up the possibility of answering some of the outstanding questions through comparative analyses.

Structurally, there is considerable variation among the 3 *Blastocystis* ST genomes now available in terms of genome size and GC-content. ST1 also displays sizeable differences compared to ST7 and ST4 with regard to the number of genes identified and general gene characteristics, such as intron numbers and gene size. However, the use of a novel mechanism by which stop codons are generated was examined thoroughly in ST1 and led us to conclude that some of the differences in gene numbers and characteristics could be due to previous annotation efforts in ST4 and ST7 not taking the stop codon issue into account.

While the need for reannotation of ST4 and ST7 affects the quality of the protein data sets from these STs to some extent, nevertheless, divergence at the amino acid level between homologous proteins has been demonstrated that is almost an order of magnitude greater than that seen between species within other genera of parasitic protistans. Analyses of protein classes of interest revealed numerous differences. Of interest will be the variation in the number and type of protease genes identified among the STs, since this class of protein has been linked to pathogenicity. Another intriguing area ripe for exploration and experimentation is the kinome. ST-specific expansions of protein kinase genes were identified. In particular, ST1 encodes a large number of novel proteins from the STE20/7 family, which is typically involved in responding to extracellular stimuli.

General aspects of the *Blastocystis* genome were examined that help in understanding its biology and how it relates to other stramenopiles and other parasitic protistans as well. Some of the highlights (see S1 Text for additional analyses) include an expanded proteome of the MRO, the absence of peroxisome-related genes, the presence of α-glucan metabolism, and intriguing expansions and additions to the repertoire of membrane-trafficking machinery proteins. The extensive structural and gene complement differences between the genomes of the 3 STs suggests the need for a revised taxonomy of *Blastocystis*. Since *Blastocystis* has been linked to disease in humans, the primary focus of any genomics analyses will likely be medically oriented. The availability of *Blastocystis* genomes will greatly assist future functional studies. In particular, the development and successful implementation of a transformation system, to allow finely tuned control over constructs as well as localization experiments, will depend on mining the genome for appropriate primers, promoters, and genes of interest. Resequencing to evaluate the success of transformations and any off-target responses will be facilitated by preexisting genomes. Pathogenomic studies of *Blastocystis* will benefit greatly from access to a genome of ST1. Comparisons with existing and future genomes from other STs and other isolates of ST1 will help in finding genes that are correlated with virulence as well as the development and testing of drug targets. Equally as important to elucidating the roles of *Blastocystis* STs in the human gut is understanding the biology of these anaerobic protists and their repertoires of metabolic pathways, which allow them to thrive in the gut environment.

## Methods and materials

### Culturing and DNA extraction

*Blastocystis* sp. NandII (ST1) was obtained from the American Type Culture Collection (ATCC 50177) and maintained in Locke's medium and horse serum egg slants at 35.6°C in an anaerobic chamber [102]. *Blastocystis* ST1 cells were harvested by centrifugation at 850xg for 15 min at 4°C. Total DNA was extracted with the standard CTAB protocol [103]. Subsequently, Hoechst dye cesium chloride (CsCl) centrifugation was used to obtain purified nuclear DNA. Extracted DNA was diluted with TE buffer to a final volume of 10 mL and resuspended in 11.5 g of CsCl. Ten mg of Hoechst dye was added and the mix was homogenized by shaking for 3 hours. The solution was then transferred into Quick-Seal centrifuge tubes and centrifuged in a fixed-angle Ti-75 rotor at 40,000xg for 44 h. The resulting DNA bands were visualized under long-wave UV light. The AT-rich organellar band was removed and the remaining nuclear DNA band retrieved with a 30-gauge needle. Hoechst dye was removed with 3 successive washes of water-saturated butanol. DNA was precipitated with 100% ethanol and suspended in water.

### RNA extraction, sequencing, and assembly

Total RNA was isolated using a modified Trizol protocol [104], in which the supernatant, after the first round, underwent another Trizol extraction. The resulting RNA was used to create a cDNA paired-end library (Vertis Biotechnologies AG [Germany]) that was then sequenced on a 4SLX Titanium Platform (Genome Quebec). The reads were filtered for primer, vector, and adaptor sequences. Following filtering, 69,155,000 read pairs remained. A second cDNA library was constructed (Beijing Genomics Institute) and RNA-Seq data was generated using the Illumina HiSeq 2000 system. As above, the reads were filtered for primer, vector, and adaptor sequences. The reads were also filtered by quality scores using FASTX-Toolkit (version 0.0.13) http://hannonlab.cshl.edu/fastx_toolkit/index.html. To be retained, a minimum of 70% of the bases had to have quality scores of 20 or better [99]. Ultimately, 370,469,956 reads remained.

The miraEST [105] and Trinity [106] assembly software programs were used to assemble the paired-end reads into contigs. 20,426 contigs were assembled. Only contigs above 200 bps were considered for further analysis. The longest contig was 18,604 bps and N50 was 793 bps.

### Genome sequencing and assembly

The *Blastocystis* ST1 genome was sequenced using 454 pyrosequencing and Illumina technologies. Illumina sequencing entailed generation of a mate paired-end library with an insert size of 3 kb. Following the filtering steps described above, 1,865,740 454 and 55,617,444 Illumina reads remained. The 454 and Illumina reads were assembled using the Ray genomic assembler software [107]. Only contigs above 200 bps were retained for further analysis.

Using different combinations of 454 and Illumina reads, 3 different assemblies were created. 2F was generated from just Illumina reads and contained 13,338 contigs. 2E was generated from all reads and contained 29,673 contigs. 2D was generated just from 454 reads. The software Minimus2 from the AMOS package [108] was used to merge the 3 into a single assembly with 14,304 contigs.

### Bacterial contamination

The genome assembly for *Blastocystis* ST1 was interrogated for the presence of contigs composed of bacterial reads. For contigs of less than 500 bps, BLASTx and BLASTn [49] searches were performed. All contigs with a percent identity of 95% and higher against entries in the nr and nt NCBI databases were considered contaminants. In total, 305 contigs were removed. Large contigs were split into 5,000-bp pieces while those less than 5,000 bps in length were kept intact. All the pieces were compared, using BLASTx, against a specialized database consisting of protein sets from all publicly available bacterial genomes and protein sets from 28 eukaryotic genomes drawn mainly from Chromalveolata and Archaeplastida. Pieces that only had matches for bacterial proteins were putatively designated as derived from bacterial contamination. Because of the possibility of LGT from bacteria, all of the 5,000-bp pieces from a large contig were required to be “bacterial” to warrant removal of the contig. In total, 459 contigs were set aside as being bacterial in nature and most probably resulting from bacterial contamination. 150 were also removed as clearly derived from contaminating *Saccharomyces cerevisiae* and *E*. *histolytica* DNA.

### Scaffolding

After the removal of bacterial/yeast/*Entamoeba* contamination, attempts were made to assemble the ST1 contigs into larger scaffolds. SSPACE [109], which is specially designed to work with preassembled contigs, was used on those contigs greater than 500 bps. The 2,494 contigs were assembled into 693 scaffolds with the N50 increasing from 8,801 bps to 50,205. The *Blastocysti*s ST1 genome sequences are available from the NCBI with the accession LXWW00000000.

### Estimation of genome coverage

In the absence of an experimentally determined genome size for ST1, the extent to which the final assembly represents the full genome was determined roughly using 2 methods. The first method entails taking a set of 679 conserved protein sequences derived from RNA-Seq data and matching them against the genome assembly. Of the conserved transcripts, 5.4% were not found in the genome assembly, suggesting that the current assembly encompasses 94.6% of the genome or at least the gene space of the genome [110].

The second strategy for estimating genome coverage was based on a method first used in the *Conus bullatus* genome paper [111] to estimate genome size. A database of 31.8 million 90-bp Illumina reads was used in BLASTn searches of the assembled contigs. Read coverage for contigs was estimated as (number_of_hits*read_length)/length_of_contig. The mode was determined from a histogram of the read coverages of the contigs and used to estimate genome size: (number_of_reads*read_size)/read_coverage_mode ((31,826,528*100)/153 = 18.7 Mb).

The estimated size was then compared with the assembled genome size (17.4 Mb/18.7 Mb = 93%).

Additionally, the protein data sets for the 3 genomes were used for BUSCO [112] analyses to assess their completeness based on a set of 429 highly conserved single copy eukaryotic genes. An initial BUSCO analysis was done using the ST protein data set. Any conserved genes that were considered missing were then compared against the appropriate ST genomic scaffolds to determine if the gene had been missed during annotation. Given the evolutionary distances between *Blastocystis* and the organisms used to generate the BUSCO test set, the likelihood of achieving 100% coverage was low. More useful was a comparison of the results for the 3 STs.

### Average level of heterozygosity

The average level of heterozygosity for ST1 was determined by aligning trimmed genomic reads to the genomic assembly with Bowtie 2.3.1 [113]. The program mpileup from SAMtools [114] was used to determine the read depth at each position of the assembly as well as the breakdown of the 4 possible bases. An in-house perl script was used to parse the mpileup data. Positions with less than 10X coverage were ignored. A site was deemed to be heterozygous if at least 2 different bases were present and there were at least 2 (or 3) reads with the different bases. The number of heterozygous sites was then divided by the total number of sites (with ≥10X coverage).

### Gene modeling

A set of 679 protein sequences was generated from the ST1 RNA-Seq clustered transcripts and used to train AUGUSTUS [115] on a repeat masked assembly [116]. The training set contained protein sequences with the following criteria: at least 1 intron, less than 80% identical at the amino acid level to any other sequence in the set, and a start site based on highly similar eukaryotic protein sequences. The script optimize_augustus.pl was also run to create species-specific meta parameters. A set of 32,269 Trinity [106] assembled RNA-Seq transcripts were used to generate a hints file for donor/acceptor intron sites. Subsequent gene modeling generated 5,637 gene models, which were then corrected with PASA [117] based on RNA-Seq transcripts. Because of the unusual nature of termination codons in *Blastocystis* [25], it was necessary to set the parameter stopCodonExcludedFromCDS to true. Of the final gene model set, 58% was examined manually at some point to check various aspects such as intron boundaries, stop codons, and chimeric models. The degree to which intron boundaries were confirmed by RNA-Seq data was determined by mapping RNA-Seq data to the genome assembly using Bowtie2 [113]. Using the intron boundary positions and the alignment positions of individual reads from the SAM file, an in-house perl script calculated that 70.3% of the intron boundaries were confirmed by 5 or more RNA-Seq reads (60.1% with 10 or more RNA-Seq reads).

### Functional annotation

To assist in the manual annotation of the gene models, various information was generated for each model. The models were compared against the conserved domain database [118] and domain hits as well as Pfam [119] hits were extracted from the results. The models were also compared against the NCBI protein database as well as the protein data set for ST7 using BLASTp. SignalP results were generated using a locally installed version of SignalP. The genome browser Genomeview [120] was chosen for annotation because of its relative ease in setting up and populating with information via gff files and the ability to alter the information using custom scripts. The genome browser also included tracks for genome reads mapped to the scaffolds (Bowtie2 [113] and SAMtools [114]), genome read map coverage, RNA-Seq reads mapped to the scaffolds, and RNA-Seq map coverage.

Because of the possibility of missed genes, the intergenic spacers were identified and the sequences compared against the NCBI protein database (nr) as well as the ST7 protein data set using BLASTx. Potentially missed genes were mapped to the scaffolds and displayed in the genome browser for confirmation and correction through manual annotation. The ST7 protein data set was also compared to the gene models for ST1 using BLASTp and the results mapped to the scaffolds as a genome browser track.

### Comparative genomics

Automated annotation for eukaryote genomes, particularly those from nonmodel organisms and poorly sampled lineages, are prone to missed genes, i.e., the gene-finding algorithms fail to find all of the protein-coding regions. While having a transcriptome is invaluable for reducing the level of missed genes, invariably, some are not found. Therefore, as a general rule, the various analyses detailed below that involve presence/absence of genes, particularly when comparing STs, used techniques such as tBLASTn to interrogate the full genome sequence before declaring that a gene was missing from an ST. Additional steps to confirm the presence/absence of a gene are indicated in the individual sections.

### GC content

The genomic scaffolds for STs 1, 4, and 7 were ordered according to their lengths. They were further stripped of their *N*s and header lines. The program GC-Profile [121] as implemented at http://tubic.tju.edu.cn/GC-Profile/ was used to create GC profile and GC content graphs for the 3 genomes. As recommended by the authors, a halting parameter of 300 and a minimum segmentation length of 3,000 bps were used.

GC-Profile, a windowless method, was chosen from many different algorithms to avoid some of the disadvantages present in other methods to calculate GC content such as GC patterns dependent on window size, and lack of resolution [121,122].

### Read-depth coverage SNP

The SAMtools option depth [114] was used to determine the read-depth coverage at each position of the genome scaffolds. These values were used to calculate median read-depth coverage and normalized median read-depth coverage using in-house scripts. SNPs were detected using the SAMtools options mpileup and bcftools.

### Amino acid divergence between STs

The level of amino acid divergence between *Blastocystis* protein sequence data sets was analyzed using reciprocal best BLAST hit protein sequence pairs. Because of concerns about retained introns in some of the STs as well as issues with incorrect stop codons, the calculation of amino acid identity scores for gene pairs was restricted to high-scoring segment pairs without the inclusion of gaps. The values were generated using an in-house perl script.

### Intron categories

For *Blastocystis* ST1, the analysis of different intron categories was based on the final annotation of the genome, incorporating data from RNA-Seq and extensive manual curation. To analyze introns in the *Blastocystis* ST7 genome, the available annotation of the genome could not be relied on because it proved to be inaccurate in many cases. Therefore, analysis was restricted to introns that have direct support from EST sequences. Available ESTs were aligned to the genome sequence using STAR [123] and introns indicated by the alignments were identified and manually corrected if needed. Homologs of protein subunits specific for the minor spliceosome were identified by BLAST (BLASTp, tBLASTn) using sequences of previously defined subunits from *Homo sapiens* and plants. The identity of the candidate orthologs was confirmed by reciprocal BLASTp searches against the nr database at NCBI. Incorrect or missing gene models in *Blastocystis* ST1 were corrected or added to the annotation of the genome. Most of the identified homologs in *Blastocystis* ST7 are either represented by incorrect gene models or completely missing from the annotated genes but could be found in the assembly with tBLASTn. Models for snRNA in both STs were identified using the program *cmscan* from the Infernal package [124].

### Mitochondrial protein predictions

A multipronged approach was used to predict mitochondrial proteins. (1) Each gene model and translated transcript was queried for N-terminal mitochondrial targeting sequences using Mitoprot and TargetP [125,126]. Sequences that returned a score greater than 0.5 were further investigated as MRO candidates. (2) The top 10 BLAST hits retrieved from the nr database at NCBI were surveyed for any mitochondrial annotation and manually investigated. (3) All gene models and translated transcripts were queried against a local curated MitoMiner data set [127] and significant hits (e-value < 1e-10) were further investigated. (4) Finally, a local MRO protein data set from various anaerobic protists including *Pygsuia biforma*, *T*. *vaginalis*, and *Mastigamoeba balamuthi* was queried against the gene models and translated transcript data sets. Mitochondrial targeting sequence scores and protein annotations are provided in S3 Data. Those proteins previously unrecognized as mitochondrial in *Blastocystis* species are shown in color in Fig 7.

### Cell cycle

Homologs of proteins suspected to be involved in cytokinesis were identified by BLASTp against the *Blastocystis* ST1 predicted proteome (e-value cutoff: 10e-10), using as queries previously identified mammalian orthologs [128]. Absence of homologs was verified by tBLASTn against the genomic scaffolds and transcriptome. *Blastocystis* hits were then used as queries to run reversed BLASTp searches against nr and results were manually inspected to confirm assignation to a given protein family. Identification of homologs of the APC/C complex and targets was carried out in a similar fashion, using as queries previously identified homologs in *Blastocystis* ST1 or, if these did not exist, various other eukaryotic homologs [78]. For calcium-binding proteins, *H*. *sapiens* homologs of protein families identified in [129] were used as queries to carry out BLASTp searches against the *Blastocystis* ST1 predicted proteome but also against all eukaryotic protein data present in GenBank as of February 2016 ("Eukaryota[Organism]" option of the BLAST+ package).

### DNA repair and meiosis

A query database including 135 DNA repair genes, meiosis-specific genes, and meiosis-related genes from a wide range of eukaryotes was established using literature and key word searches of the NCBI database. Genes with functional experimental evidence were used as queries to retrieve similar proteins using PSI-blast. After retrieval, protein sequence alignments were created and used to build HMM profiles with HMMER3.1b2 [130] to enable gene searches in *Blastocystis* STs 1, 4, and 7. Retrieval of genes was restricted to e-values below 1e-04. Phylogenetic trees were constructed with FastTree [131] using closely related paralogs and/or reconstructing entire gene families for each gene of interest. Protein domains were mapped to the trees to facilitate gene recognition. In a few cases, secondary structure predictions were made to confirm the gene identification.

### Kinome analysis

221 kinases were identified from the protein-coding gene set of *Blastocystis* ST1 using HMMER [130]. The STYKc profile (SM00221) from the SMART database [132] was used, as was the Pfam Pkinase profile (PF00069) [119]. *Blastocystis* kinases were classified by their positions in phylogenetic trees inferred from the alignments of the HMMER output (both neighbor joining and maximum likelihood trees were taken into consideration). *Blastocystis* gene models with 2 kinase domains were classified by the phylogenetic position of the domain with a better HMMER score. The kinase classification follows the webite www.kinase.com [33].

The 221 kinases identified from *Blastocystis* ST1 were used to query the protein-coding gene sets from STs 4 and 7. The annotations for STs 4 and 7 were also searched for the key word “kinase” and putative protein kinases were checked against the kinase database (www.kinase.com) using the BLAST search tool.

The resulting sequences from STs 1, 4, and 7 were aligned using MUSCLE [133], followed by manual inspection of the alignment. A maximum likelihood tree was inferred by the RAxML program [134] with the PROTGAMMALG model and visualized using Figtree (http://tree.bio.ed.ac.uk/software/figtree/).

### CYP

The *Blastocystis* STs 1 and 7 protein models were searched by BLASTp with CYP protein sequences representing the CYP2 (DmelCYP307A1, DmelCYP303A1, and DmelCYP18A1), CYP3 (HsCYP3A4, HzCYP321A1, DmelCYP6A2, DmelCYP6A8, and DmelCYP6G1), and CYP4 (DmelCYP4G15 and DmelCYP4G1) animal clades [135]. CYPs from the mitochondrial clan were searched using DmelCYP314A1, DmelCYP302A1, DmelCYP12A1, DmelCYP301A1, DmelCYP49A1, and DmelCYP315A1. Additional animal and fungi CYP clans [136], clan 51, clan 7, clan 26, clan 20, clan 46, clan 19, and clan 74, were also used to search for CYPs in the *Blastocystis* protein models as well as the CYPome from sequenced genomes of *Guillardia theta*, *T*. *pseudonana*, and *Phytophthora sojae*. CYP51 clan involved in sterol biosynthesis was further studied using evolutionarily close CYP51s including *Galdieria sulphuraria* CYP51, *Porphyridium purpureum* CYP51R1, *Batrachochytrium dendrobatidis* CYP51F1, *Cyanidioschyzon merolae* CYP51G1, *Dictyostelium discoideum* CYP516A1, *L*. *major* CYP51E1, *Monosiga brevicollis* CYP51A1, *T*. *pseudonana* CYP51C1, and *Chlamydomonas reinhardtii* CYP51G.

CYP searches for other organisms were done as above but using NCBI, CYPED (https://cyped.biocatnet.de/), and the eukaryotic pathogen database EuPathDB (http://eupathdb.org/eupathdb/) [137–139].

### Membrane-trafficking system

The components of the membrane-trafficking system in *Blastocystis* STs 1, 4, and 7 were identified using both comparative genomic and phylogenetic methods. To identify potential homologs, functionally characterized membrane-trafficking components from *H*. *sapiens* and *S*. *cerevisiae* were used as queries to search the *Blastocystis* predicted proteins using BLASTp with default search parameters. Hits with an e-value ≤5e-02 were considered candidate homologs and were used as BLAST queries to reciprocally search predicted proteins of the *H*. *sapiens* and *S*. *cerevisiae* genomes. The *Blastocystis* protein was considered homologous to the initial *H*. *sapiens* query if it retrieved the query or a clear ortholog as (i) the top hit and (ii) with an e-value ≤5e-02.

If a component could not be positively identified using BLAST searching, a hidden Markov model (HMM) was created using HMMER version 3 [140], including clear homologs from combinations of the following taxa: *H*. *sapiens* (NCBI, http://www.ncbi.nlm.nih.gov/), *S*. *cerevisiae* (NCBI), *D*. *discoideum* (dictyBase, http://dictybase.org/), *Arabidopsis thaliana* (NCBI), *C*. *reinhardtii* (Phytozome, http://www.phytozome.net/), *P*. *sojae* (JGI, http://www.jgi.doe.gov/), *T*. *pseudonana* (JGI), *Naegleria gruberi* (JGI), *Bigelowiella natans* (JGI), and *Emiliania huxleyi* (JGI). The HMMs were used to search the *Blastocystis* STs 1, 4, and 7 genomes (hmmsearch). Hits with an e-value ≤5e-02 were used as BLASTp queries to search the *H*. *sapiens* genome, and homologs were identified using the same criteria as were used in the initial BLASTp searches.

If BLASTp or HMMer failed to identify a membrane-trafficking component, tBLASTn searches were performed, using the *H*. *sapiens* and *S*. *cerevisiae* queries to search the *Blastocystis* scaffolds. The region of the scaffold with a hit that had an e-value ≤5e-02 was excised and used as a BLASTx query to search the *H*. *sapiens* genome, and homologs were identified using the same criteria as were used in the BLASTp and HMMer searches.

The more permissive e-value cutoff of 5e-02 was intended to reduce the number of false negatives from divergent *Blastocystis* genes when searching with the experimentally characterized opisthokont queries. Nonetheless, we found in post hoc assessment that over 93% of the orthologs identified had e-values of lower than 1e-05 in either the forward or reverse BLAST searches.

In searching for the components of a protein family in *Blastocystis*, Bayesian and maximum likelihood phylogenetics methods were used to more rigorously determine orthology. For each phylogeny, a combination of homologs from *H*. *sapiens*, *S*. *cerevisiae*, *T*. *pseudonana* and *P*. *sojae*, *P*. *infestans*, *A*. *thaliana*, *D*. *discoideum*, and *N*. *gruberi* were collected and aligned with the *Blastocystis* homologs using MUSCLE version 3.8.31 [133] and manually adjusted. Positions of ambiguous homology were removed (alignments available upon request) and ProtTest 3.2 [141] was used to determine the best-fit model of sequence evolution, which, in all cases, was the LG model [142], incorporating rate among site and invariant site corrections where relevant.

Phylobayes 3.3 [143] was used to produce the optimal topology and posterior probability values. Analyses were run until the average standard deviation of the split frequencies fell below 0.1 and the effective sample size was at least 100. Once convergence occurred, the first 20% of sampled trees were removed. Additionally, RAxML and, in some cases, PhyML version 3 [144] were used to obtain maximum likelihood bootstrap values (100 pseudoreplicates). Resultant phylogenetic trees were viewed using FigTree v1.4.0.

### Peroxisomal machinery

To identify putative *PEX* genes in *Blastocystis* STs 1, 4, and 7, protein sequences from *H*. *sapiens*, *S*. *cerevisiae*, and/or *Neurospora crassa* were used as queries for BLASTp and tBLASTn searches against locally hosted *Blastocystis* protein sequences and scaffolds, respectively. Candidate homologs with e-values less than or equal to 0.05 were subjected to reciprocal BLASTp searches against the locally hosted query genome as well as the locally hosted nr database. The retrieval of the original query as the top reciprocal BLASTp hit with an e-value less than or equal to 0.05 was the criteria for identification of a putative homolog.

To identify putative *PEX* genes in *A*. *thaliana* and the stramenopile genomes of *P*. *ramorum*, *T*. *pseudonana*, and *P*. *tricornutum*, *H*. *sapiens*, *S*. *cerevisiae*, and/or *N*. *crassa* protein sequences were used as queries for pHMMer [145] searches against locally hosted query genomes. Candidate homologs with e-values less than or equal to 0.05 were subjected to reciprocal pHMMer searches against the locally hosted genome and NR database. Thus, the retrieval of the original query as the top reciprocal pHMMer hit with an e-value less than or equal to 0.05 was the criteria for identification of a putative homolog. Results were compared to previously listed *PEX* genes in *A*. *thaliana* [146] and *P*. *tricornutum* [68]. Newly identified *A*. *thaliana*, *P*. *ramorum*, *T*. *pseudonana*, and *P*. *tricornutum PEX* genes were subsequently used as queries for BLASTp and tBLASTn searches against locally hosted *Blastocystis* protein sequences and scaffolds, respectively. Candidate homologs were subjected to reciprocal BLASTp searches according to the criteria described above.

### Identification and analysis of CAZymes

The CAZy annotation pipeline was used to analyze 5,966 predicted proteins from the *Blastocystis* ST1 genome using a 2-step procedure of identification and annotation [147]. Sequences were subjected to BLASTp analysis against the CAZy database, composed of full-length proteins. Hits with an e-value <0.1 were then subjected to a modular annotation procedure using BLASTp against libraries of catalytic and carbohydrate binding modules and profile HMMs [148]. The results were complemented with signal peptide, transmembrane, and glycosylphosphatidylinisotol (GPI) anchor predictions [149,150]. Fragmentary gene models and all models suspected of containing errors were identified and flagged. A final functional annotation step involved performing BLASTp comparisons against a library of protein modules derived from biochemically characterized enzymes [147]. Three other stramenopile species, the diatom *T*. *pseudonana*, and the oomycetes, *Albugo laibachii* Nc14 and *P*. *infestans* T30-4, are present in the CAZy database and were used for comparison. The predicted CAZymes encoded by the genome of the brown alga *Ectocarpus siliculosus* (http://bioinformatics.psb.ugent.be/orcae/overview/Ectsi) were also annotated using the same procedures for comparison with other stramenopiles.

### Intermediary metabolism homology probing

To predict various pathways related to intermediary metabolism (e.g., nucleotide, amino acid, cofactor metabolism, etc.), the predicted gene models and RNA-Seq transcripts were annotated using the KEGG automated annotation server (KAAS) using the single-directional best hit approach (see http://www.genome.jp/tools/kaas/). Each pathway was individually searched for completeness and results are summarized in S8 Data. In some cases, in which KAAS did not predict an ortholog capable of a reaction, query sequences were retrieved from KEGG and manually searched against the scaffold and transcriptome data.

### Secretome

All predicted gene models were run through SignalP with a threshold of 0.70. The selected models were then run through TargetP in order to identify a secretory pathway signal peptide with a cutoff of 0.70. Subsequently, the remaining models were run through WoLF PSORT [151] and only those predicted as extracellular were retained. As a final step, TMHMM [152] was used as a final checkpoint to find and remove any model with predicted transmembrane domains. Therefore, the secretome was identified as having a secretory pathway signal peptide, extracellular localization, and absence of transmembrane regions.

### Proteases

All gene models were run through the MEROPS database [51]. Only the models with predicted active sites were considered. Annotation and naming of the models follows the MEROPS database terminology (S2 Data).

### Flagellar-associated proteins

A reciprocal best BLAST analysis was performed for the protein data sets from *Blastocystis* ST1 and *C*. *reinhardtii* using the program orthoparahomlist.pl with default settings (Stanke M. Orthoparahomlist.pl script. 2011. https://github.com/goshng/RNASeqAnalysis/blob/master/pl/orthoparahomlist.pl). The resulting list of orthologs was compared against the 213 *C*. *reinhardtii* conserved ciliary proteins [153] to determine which of the orthologs found in *Blastocystis* ST1 had matches.

### GTPases

Members of the Ras superfamily were searched for in the 3 *Blastocystis* genomes using well-characterized representative sequences from other eukaryotes (*H*. *sapiens*, *P*. *sojae*, and others) as queries. Both the databases of predicted protein sequences (using BLASTp) and the genome sequence assemblies (using tBLASTn) were searched to ensure that no gene was missed because of the lack of a corresponding protein prediction. Indeed, a number of genes that were missing in the protein sequence databases were identified in all 3 STs. The missing or inaccurate gene models in ST1 were manually predicted or corrected using various lines of evidence (transcriptomic data and comparisons to homologs) and incorporated into the main genome annotation. Models missing for the ST4 and ST7 genomes were constructed only when the phylogenetic relationship to ST1 genes was unclear from the tBLASTn searches. Orthologous and paralogous relationships among *Blastocystis* Ras superfamily genes were established primarily on the basis of reciprocal BLAST searches, but phylogenetic analyses were required in a few cases. Orthology to conserved eukaryotic groups of GTPases was, in most cases, obvious from BLAST comparisons, except for several highly divergent paralogs related or similar to Rab GTPases, which most likely represent lineage-specific, rapidly evolving genes derived from duplications of some common Rab genes. A more detailed analysis was carried out for the *Blastocystis* family of Miro proteins. These sequences and Miro homologs from 2 newly analyzed stramenopile lineages (Labyrinthulea, represented by *Aurantichytrium limacisporum*, and Placididea, represented by *Cafeteria* sp. Caron lab isolate) were added to a selection of Miro sequences previously analyzed by Vlahou et al. (2011) [63], excluding the very divergent sequences of *N*. *gruberi*, ciliates, and trypanosomatids. The sequences were aligned using MAFFT (version 7, http://mafft.cbrc.jp/alignment/server/, [154]) and the alignment was trimmed using the Gblocks server (http://molevol.cmima.csic.es/castresana/Gblocks_server.html, [155]) with the least stringent parameters, keeping 245 aligned amino acid positions. The tree was calculated using RAxML-HPC (8.2.8) run at the Cyberinfrastructure for Phylogenetic Research (CIPRES) Portal (http://www.phylo.org/sub_sections/portal/), employing the LG+Г+F substitution model and rapid bootstrapping followed by a thorough search for the optimal tree.

## Supporting information

S1 Text Supplemental Analyses. (DOCX)

S1 Fig Genome browser screengrab of overlapping genes models in *Blastocystis* ST4. The 3′ ends of KNB46045 and KNB46046 overlap on scaffold NW_014569526. The presently annotated stop codon for KNB46045 (TGA) is represented by a red box with a dark blue outline. Where polyadenylation starts and creates an alternative stop codon (TAA) is indicated with a transparent blue circle and the motif (TGTTTGTT) that determines where the polyadenylation would start is denoted by an orange bordered box.
(DOCX)

S2 Fig Sequence logo of signal peptides in 89 *Blastocystis* ST1 secreted proteins. (DOCX)

S3 Fig Presence of components of the membrane-trafficking machinery in *Blastocystis* ST1, ST4, and ST7 compared with the last eukaryotic common ancestor (LECA). The numbers within the pie slices indicate the number of isoforms.
(DOCX)

S4 Fig General model for meiotic recombination in *Blastocystis* ST1. **Abbreviations:** CO, crossover; DSB, double-strand break; NCO, noncrossover.
(DOCX)

S5 Fig Distribution of allele frequencies of heterozygous sites across *Blastocystis* ST1 genomic scaffolds. (DOCX)

S6 Fig Median read depth of scaffolds greater than 10,000 bps in *Blastocystis* ST1. Read depth was scaled to give the median value (113) of the scaffold medians a value of 1.00.
(DOCX)

S7 Fig Distribution of read depth along the nondisomic scaffold 113. Y-axis corresponds to read depth, x-axis shows scaffold position. Upper bar denotes median read depth for scaffold 113 (213), while lower bar indicates median read depth (113) of all scaffolds over 10,000 bps.
(DOCX)

S8 Fig Distribution of read depth along disomic scaffold 102. Y-axis corresponds to read depth, x-axis shows scaffold position. Horizontal bar denotes median read depth for scaffold 102 as well as indicating median read depth (113) of all scaffolds over 10,000 bps.
(DOCX)

S9 Fig 40S ribosomal protein S23 and flanking genes in *Blastocystis* ST1 and ST7. The middle gene corresponds to RPS23. ST1 genes are in blue, ST7 in red. ST1 has 6 copies of RPS23, all of which differ from each other at the nucleotide level. The 3 copies of RPS23 in ST7 are aligned with the corresponding copy in ST1 based on similar flanking genes. Two ST1 copies (AV274_5206 and AV274_5757) do not have a corresponding ST7 copy and, based on the arrangement of flanking genes, represent either insertions in ST1 or deletions in ST7.
(DOCX)

S10 Fig GC profile and GC content graphs for *Blastocystis* ST1, ST4, and ST7. GC profile graphs (A1, B1, and C1 for ST1, ST4, and ST7, respectively) are based on a windowless method. Straight lines denote a region of relatively homogenous GC content. An upward line indicates an abrupt increase in GC content while a downward line indicates an abrupt decrease in GC-content. GC content graphs (A2, B2, and C2 for ST1, ST4, and ST7, respectively) indicate the percentage of GC bases across a segment. The graphs were generated using http://tubic.tju.edu.cn/GC-Profile/ with a halting parameter of 300 and a minimum segment length of 3,000.
(DOCX)

S1 Table Minor spliceosome-specific components in *Blastocystis* ST1 and ST7. (DOCX)

S2 Table Median sequence identity of matching regions of orthologous proteins from pairs of protozoan pathogens. (DOCX)

S3 Table α-glucan-related genes in *Cafeteria* transcriptomes. (DOCX)

S4 Table Telomerase-related genes in *Blastocystis* STs 1, 4, and 7. (DOCX)

S5 Table Telomeric repeats (TTAGGG) in *Blastocystis* ST1. (DOCX)

S6 Table Copy numbers of 40S ribosomal proteins in *Blastocystis* ST1 and ST7. (DOCX)

S7 Table Copy numbers of 60S ribosomal proteins in *Blastocystis* ST1 and ST7. (DOCX)

S8 Table Carbohydrate active enzymes (CAZymes) present in *Blastocystis* ST1 and selected stramenopile genomes. **Abbreviations:** CBM, carbohydrate-binding modules; CE, carbohydrate esterases; GH, glycosyl hydrolases; GT, glycosyl transferases; PL, polysaccharide lyase.
(DOCX)

S9 Table Number of different CAZy families found in *Blastocystis* ST1 and selected stramenopile genomes. (DOCX)

S10 Table *Blastocystis* ST1 protein models with matches to a *Chlamydomonas reinhardtii* flagellar data set. (DOCX)

S1 Data Secretome for *Blastocystis* ST1. (XLSX)

S2 Data Classification of proteases—*Blastocystis* STs 1, 4, and 7. (XLSX)

S3 Data Mitochondrial predicted proteome—*Blastocystis* ST1. (XLSX)

S4 Data Ras superfamily GTPases in *Blastocystis* STs 1, 4, and 7. (XLS)

S5 Data Anaphase promoting complex (APC) subunits—*Blastocystis* ST1. (XLSX)

S6 Data DNA repair and meiosis-specific and related loci in *Blastocystis* STs 1, 4, and 7. (XLSX)

S7 Data Carbohydrate active enzymes—*Blastocystis* ST1. (XLSX)

S8 Data Intermediary metabolism—*Blastocystis* ST1. (XLSX)

S9 Data Proteins suspected to be involved in cytokinesis—*Blastocystis* ST1. (XLSX)

S10 Data Calcium-associated proteins—*Blastocystis* ST1. (XLSX)

S11 Data Introns in *Blastocystis* STs 1, 4, and 7. (XLSX)

S12 Data Amino acid identities for orthologs. (TXT)

## Abbreviations

ACS: acetyl-CoA synthetase
APC/C: anaphase promoting complex/cyclosome
BLAST: Basic Local Alignment Search Tool
BUSCO: Benchmarking Universal Single-Copy Orthologs
Cdh1: cadherin-1
CAMKL: calcium/calmodulin-dependent-like
CAZy: database of carbohydrate-active enzymes
CAZyme: carbohydrate active enzyme
Cdc20: cell-division cycle protein 20
CYP: cytochrome P450
ER: endoplasmic reticulum
ESCRT: endosomal sorting complexes required for transport
EST: expressed sequence tag
e-value: expect value
GC: guanine-cytosine
GH: glycoside hydrolase
GT: glycosyltransferase
HMP: hydroxymethylpyrimidine
iDBE: indirect debranching enzyme
IgA: immunoglobulin A
KOG: eukaryotic orthologous groups
LECA: last eukaryotic common ancestor
LGT: lateral gene transfer
MAPK: mitogen-activated protein kinase
MRO: mitochondrion-related organelle
PFO: pyruvate:ferredoxin oxidoreductase
RAxML: Randomized Axelerated Maximum Likelihood
RNA-Seq: RNA-sequencing
snRNA: small nuclear RNA
SRβ: membrane-anchored β subunit
SSU: small subunit
ST: subtype
ThiM: hydroxyethylthiazole kinase
TPR: tetratricopeptide repeat
TPS: trehalose phosphate synthetase

## References

[1] Scanlan PD, Stensvold CR. Blastocystis: getting to grips with our guileful guest. Trends Parasitol. 2013 11;29(11):523–9. doi: 10.1016/j.pt.2013.08.006 24080063 10.1016/j.pt.2013.08.006

[2] Wawrzyniak I, Poirier P, Viscogliosi E, Dionigia M, Texier C, Delbac F, et al Blastocystis, an unrecognized parasite: an overview of pathogenesis and diagnosis. Ther Adv Infect Dis. 2013 10;1(5):167–78. doi: 10.1177/2049936113504754 25165551 10.1177/2049936113504754 PMC4040727

[3] Roberts T, Stark D, Harkness J, Ellis J. Update on the pathogenic potential and treatment options for Blastocystis sp. Gut Pathog. 2014 5 28;6:17,4749-6-17. eCollection 2014. 10.1186/1757-4749-6-17 PMC4039988 24883113

[4] Scanlan PD, Stensvold CR, Rajilic-Stojanovic M, Heilig HG, De Vos WM, O'Toole PW, et al The microbial eukaryote Blastocystis is a prevalent and diverse member of the healthy human gut microbiota. FEMS Microbiol Ecol. 2014 10;90(1):326–30. doi: 10.1111/1574-6941.12396 25077936 10.1111/1574-6941.12396

[5] Alfellani MA, Stensvold CR, Vidal-Lapiedra A, Onuoha ES, Fagbenro-Beyioku AF, Clark CG. Variable geographic distribution of Blastocystis subtypes and its potential implications. Acta Trop. 2013 4;126(1):11–8. doi: 10.1016/j.actatropica.2012.12.011 23290980 10.1016/j.actatropica.2012.12.011

[6] Stensvold CR, Clark CG. Current status of Blastocystis: A personal view. Parasitol Int. 2016 12;65(6 Pt B):763–71. 27247124 10.1016/j.parint.2016.05.015

[7] Ajjampur SS, Tan KS. Pathogenic mechanisms in Blastocystis spp.—Interpreting results from in vitro and in vivo studies. Parasitol Int. 2016 12;65(6 Pt B):772–9. 27181702 10.1016/j.parint.2016.05.007

[8] Nourrisson C, Wawrzyniak I, Cian A, Livrelli V, Viscogliosi E, Delbac F, et al On Blastocystis secreted cysteine proteases: a legumain-activated cathepsin B increases paracellular permeability of intestinal Caco-2 cell monolayers. Parasitology. 2016 9 9:1–10. 10.1017/S0031182016001396 27609526

[9] Wu Z, Mirza H, Tan KS. Intra-subtype variation in enteroadhesion accounts for differences in epithelial barrier disruption and is associated with metronidazole resistance in Blastocystis subtype-7. PLoS Negl Trop Dis. 2014 5 22;8(5):e2885 doi: 10.1371/journal.pntd.0002885 24851944 10.1371/journal.pntd.0002885 PMC4031124

[10] Mohamed RT, El-Bali MA, Mohamed AA, Abdel-Fatah MA, El-Malky MA, Mowafy NM, et al Subtyping of Blastocystis sp. isolated from symptomatic and asymptomatic individuals in Makkah, Saudi Arabia. Parasit Vectors. 2017 4 7;10(1):174,017-2114-8. 10.1186/s13071-017-2114-8 PMC5383971 28388938

[11] Alinaghizade A, Mirjalali H, Mohebali M, Stensvold CR, Rezaeian M. Inter- and intra-subtype variation of Blastocystis subtypes isolated from diarrheic and non-diarrheic patients in Iran. Infect Genet Evol. 2017 6;50:77–82. doi: 10.1016/j.meegid.2017.02.016 28238960 10.1016/j.meegid.2017.02.016

[12] Seyer A, Karasartova D, Ruh E, Gureser AS, Turgal E, Imir T, et al Epidemiology and Prevalence of Blastocystis spp. in North Cyprus. Am J Trop Med Hyg. 2017 5;96(5):1164–70. doi: 10.4269/ajtmh.16-0706 28167596 10.4269/ajtmh.16-0706 PMC5417212

[13] Andersen LO, Bonde I, Nielsen HB, Stensvold CR. A retrospective metagenomics approach to studying Blastocystis. FEMS Microbiol Ecol. 2015 7;91(7): doi: 10.1093/femsec/fiv072 Epub 2015 Jun 29. 26130823 10.1093/femsec/fiv072

[14] Audebert C, Even G, Cian A, Blastocystis Investigation Group, Loywick A, Merlin S, et al Colonization with the enteric protozoa Blastocystis is associated with increased diversity of human gut bacterial microbiota. Sci Rep. 2016 5 5;6:25255 doi: 10.1038/srep25255 27147260 10.1038/srep25255 PMC4857090

[15] Nourrisson C, Scanzi J, Pereira B, NkoudMongo C, Wawrzyniak I, Cian A, et al Blastocystis is associated with decrease of fecal microbiota protective bacteria: comparative analysis between patients with irritable bowel syndrome and control subjects. PLoS ONE. 2014 11 3;9(11):e111868 doi: 10.1371/journal.pone.0111868 25365580 10.1371/journal.pone.0111868 PMC4218853

[16] Nagel R, Traub RJ, Allcock RJ, Kwan MM, Bielefeldt-Ohmann H. Comparison of faecal microbiota in Blastocystis-positive and Blastocystis-negative irritable bowel syndrome patients. Microbiome. 2016 8 31;4(1):47,016-0191-0. 10.1186/s40168-016-0191-0 PMC5007835 27580855

[17] Perez-Brocal V, Clark CG. Analysis of two genomes from the mitochondrion-like organelle of the intestinal parasite Blastocystis: complete sequences, gene content, and genome organization. Mol Biol Evol. 2008 11;25(11):2475–82. doi: 10.1093/molbev/msn193 18765437 10.1093/molbev/msn193 PMC2568035

[18] Stechmann A, Hamblin K, Perez-Brocal V, Gaston D, Richmond GS, van der Giezen M, et al Organelles in Blastocystis that blur the distinction between mitochondria and hydrogenosomes. Curr Biol. 2008 4 22;18(8):580–5. doi: 10.1016/j.cub.2008.03.037 18403202 10.1016/j.cub.2008.03.037 PMC2428068

[19] Wawrzyniak I, Roussel M, Diogon M, Couloux A, Texier C, Tan KS, et al Complete circular DNA in the mitochondria-like organelles of Blastocystis hominis. Int J Parasitol. 2008 10;38(12):1377–82. doi: 10.1016/j.ijpara.2008.06.001 18694756 10.1016/j.ijpara.2008.06.001

[20] Jacob AS, Andersen LO, Pavinski Bitar P, Richards VP, Shah S, Stanhope MJ, et al Blastocystis mitochondrial genomes appear to show multiple independent gains and losses of start and stop codons. Genome Biol Evol. 2016 11 3. 10.1093/gbe/evw255 PMC5203790 27811175

[21] Denoeud F, Roussel M, Noel B, Wawrzyniak I, Da Silva C, Diogon M, et al Genome sequence of the stramenopile Blastocystis, a human anaerobic parasite. Genome Biol. 2011;12(3):R29,2011-12-3-r29. Epub 2011 Mar 25. 10.1186/gb-2011-12-3-r29 PMC3129679 21439036

[22] Wawrzyniak I, Courtine D, Osman M, Hubans-Pierlot C, Cian A, Nourrisson C, et al Draft genome sequence of the intestinal parasite Blastocystis subtype 4-isolate WR1. Genom Data. 2015 2 2;4:22–3. doi: 10.1016/j.gdata.2015.01.009 26484170 10.1016/j.gdata.2015.01.009 PMC4535960

[23] Jerlstrom-Hultqvist J, Franzen O, Ankarklev J, Xu F, Nohynkova E, Andersson JO, et al Genome analysis and comparative genomics of a Giardia intestinalis assemblage E isolate. BMC Genomics. 2010 10 7;11:543,2164-11-543. 10.1186/1471-2164-11-543 PMC3091692 20929575

[24] Rogers MB, Hilley JD, Dickens NJ, Wilkes J, Bates PA, Depledge DP, et al Chromosome and gene copy number variation allow major structural change between species and strains of Leishmania. Genome Res. 2011 12;21(12):2129–42. doi: 10.1101/gr.122945.111 22038252 10.1101/gr.122945.111 PMC3227102

[25] Klimes V, Gentekaki E, Roger AJ, Elias M. A large number of nuclear genes in the human parasite blastocystis require mRNA polyadenylation to create functional termination codons. Genome Biol Evol. 2014 7 10;6(8):1956–61. doi: 10.1093/gbe/evu146 25015079 10.1093/gbe/evu146 PMC4159000

[26] Rogozin IB, Carmel L, Csuros M, Koonin EV. Origin and evolution of spliceosomal introns. Biol Direct. 2012 4 16;7:11,6150-7-11. 10.1186/1745-6150-7-11 PMC3488318 22507701

[27] Shoguchi E, Shinzato C, Kawashima T, Gyoja F, Mungpakdee S, Koyanagi R, et al Draft assembly of the Symbiodinium minutum nuclear genome reveals dinoflagellate gene structure. Curr Biol. 2013 8 5;23(15):1399–408. doi: 10.1016/j.cub.2013.05.062 23850284 10.1016/j.cub.2013.05.062

[28] Will CL, Schneider C, Hossbach M, Urlaub H, Rauhut R, Elbashir S, et al The human 18S U11/U12 snRNP contains a set of novel proteins not found in the U2-dependent spliceosome. RNA. 2004 6;10(6):929–41. doi: 10.1261/rna.7320604 15146077 10.1261/rna.7320604 PMC1370585

[29] Turunen JJ, Niemela EH, Verma B, Frilander MJ. The significant other: splicing by the minor spliceosome. Wiley Interdiscip Rev RNA. 2013 Jan-Feb;4(1):61–76. doi: 10.1002/wrna.1141 23074130 10.1002/wrna.1141 PMC3584512

[30] Noel C, Dufernez F, Gerbod D, Edgcomb VP, Delgado-Viscogliosi P, Ho LC, et al Molecular phylogenies of Blastocystis isolates from different hosts: implications for genetic diversity, identification of species, and zoonosis. J Clin Microbiol. 2005 1;43(1):348–55. doi: 10.1128/JCM.43.1.348-355.2005 15634993 10.1128/JCM.43.1.348-355.2005 PMC540115

[31] Yoshikawa H, Koyama Y, Tsuchiya E, Takami K. Blastocystis phylogeny among various isolates from humans to insects. Parasitol Int. 2016 12;65(6 Pt B):750–9. 27091546 10.1016/j.parint.2016.04.004

[32] Manning G, Plowman GD, Hunter T, Sudarsanam S. Evolution of protein kinase signaling from yeast to man. Trends Biochem Sci. 2002 10;27(10):514–20. 12368087 10.1016/s0968-0004(02)02179-5

[33] Manning G, Whyte DB, Martinez R, Hunter T, Sudarsanam S. The protein kinase complement of the human genome. Science. 2002 12 6;298(5600):1912–34. doi: 10.1126/science.1075762 12471243 10.1126/science.1075762

[34] Chuang WL, Lin PY, Lin HC, Chen YL. The Apoptotic Effect of Ursolic Acid on SK-Hep-1 Cells is Regulated by the PI3K/Akt, p38 and JNK MAPK Signaling Pathways. Molecules. 2016 4 20;21(4):460 doi: 10.3390/molecules21040460 27104510 10.3390/molecules21040460 PMC6274268

[35] Ellis JG 4th, Davila M, Chakrabarti R. Potential involvement of extracellular signal-regulated kinase 1 and 2 in encystation of a primitive eukaryote, Giardia lamblia. Stage-specific activation and intracellular localization. J Biol Chem. 2003 1 17;278(3):1936–45. doi: 10.1074/jbc.M209274200 12397063 10.1074/jbc.M209274200

[36] Hua SB, Wang CC. Interferon-gamma activation of a mitogen-activated protein kinase, KFR1, in the bloodstream form of Trypanosoma brucei. J Biol Chem. 1997 4 18;272(16):10797–803. 9099733 10.1074/jbc.272.16.10797

[37] Roman E, Arana DM, Nombela C, Alonso-Monge R, Pla J. MAP kinase pathways as regulators of fungal virulence. Trends Microbiol. 2007 4;15(4):181–90. doi: 10.1016/j.tim.2007.02.001 17321137 10.1016/j.tim.2007.02.001

[38] Yu Z, An B, Ramshaw JA, Brodsky B. Bacterial collagen-like proteins that form triple-helical structures. J Struct Biol. 2014 6;186(3):451–61. doi: 10.1016/j.jsb.2014.01.003 24434612 10.1016/j.jsb.2014.01.003 PMC4096566

[39] Caswell CC, Barczyk M, Keene DR, Lukomska E, Gullberg DE, Lukomski S. Identification of the first prokaryotic collagen sequence motif that mediates binding to human collagen receptors, integrins α_2_β_1_ and α_11_β_1_. J Biol Chem. 2008 12 26;283(52):36168–75. doi: 10.1074/jbc.M806865200 18990704 10.1074/jbc.M806865200 PMC2605992

[40] Paterson GK, Nieminen L, Jefferies JM, Mitchell TJ. PclA, a pneumococcal collagen-like protein with selected strain distribution, contributes to adherence and invasion of host cells. FEMS Microbiol Lett. 2008 8;285(2):170–6. doi: 10.1111/j.1574-6968.2008.01217.x 18557785 10.1111/j.1574-6968.2008.01217.x

[41] Sylvestre P, Couture-Tosi E, Mock M. A collagen-like surface glycoprotein is a structural component of the Bacillus anthracis exosporium. Mol Microbiol. 2002 7;45(1):169–78. 12100557 10.1046/j.1365-2958.2000.03000.x

[42] Dunn LA, Boreham PF, Stenzel DJ. Ultrastructural variation of Blastocystis hominis stocks in culture. Int J Parasitol. 1989 2;19(1):43–56. 2707962 10.1016/0020-7519(89)90020-9

[43] Zaman V. Phase-contrast microscopy of cell division in Blastocystis hominis. Ann Trop Med Parasitol. 1997 3;91(2):223–4. 9307666 10.1080/00034983.1997.11813134

[44] Zaman V, Howe J, Ng M, Goh TK. Scanning electron microscopy of the surface coat of Blastocystis hominis. Parasitol Res. 1999 12;85(12):974–6. 10599919 10.1007/s004360050668

[45] Klemba M, Goldberg DE. Biological roles of proteases in parasitic protozoa. Annu Rev Biochem. 2002;71:275–305. doi: 10.1146/annurev.biochem.71.090501.145453 12045098 10.1146/annurev.biochem.71.090501.145453

[46] Puthia MK, Vaithilingam A, Lu J, Tan KS. Degradation of human secretory immunoglobulin A by Blastocystis. Parasitol Res. 2005 11;97(5):386–9. doi: 10.1007/s00436-005-1461-0 16151742 10.1007/s00436-005-1461-0

[47] Puthia MK, Lu J, Tan KS. Blastocystis ratti contains cysteine proteases that mediate interleukin-8 response from human intestinal epithelial cells in an NF-kappaB-dependent manner. Eukaryot Cell. 2008 3;7(3):435–43. doi: 10.1128/EC.00371-07 18156286 10.1128/EC.00371-07 PMC2268520

[48] Sajid M, McKerrow JH. Cysteine proteases of parasitic organisms. Mol Biochem Parasitol. 2002 3;120(1):1–21. 11849701 10.1016/s0166-6851(01)00438-8

[49] Altschul SF, Madden TL, Schaffer AA, Zhang J, Zhang Z, Miller W, et al Gapped BLAST and PSI-BLAST: a new generation of protein database search programs. Nucleic Acids Res. 1997 9 1;25(17):3389–402. 9254694 10.1093/nar/25.17.3389 PMC146917

[50] Mottram JC, Coombs GH, Alexander J. Cysteine peptidases as virulence factors of Leishmania. Curr Opin Microbiol. 2004 8;7(4):375–81. doi: 10.1016/j.mib.2004.06.010 15358255 10.1016/j.mib.2004.06.010

[51] Rawlings ND, Waller M, Barrett AJ, Bateman A. MEROPS: the database of proteolytic enzymes, their substrates and inhibitors. Nucleic Acids Res. 2014 1;42(Database issue):D503–9. doi: 10.1093/nar/gkt953 24157837 10.1093/nar/gkt953 PMC3964991

[52] Rendon-Gandarilla FJ, Ramon-Luing Lde L, Ortega-Lopez J, Rosa de Andrade I, Benchimol M, Arroyo R. The TvLEGU-1, a legumain-like cysteine proteinase, plays a key role in Trichomonas vaginalis cytoadherence. Biomed Res Int. 2013;2013:561979 doi: 10.1155/2013/561979 23509742 10.1155/2013/561979 PMC3581150

[53] Baker RP, Wijetilaka R, Urban S. Two Plasmodium rhomboid proteases preferentially cleave different adhesins implicated in all invasive stages of malaria. PLoS Pathog. 2006 10;2(10):e113 doi: 10.1371/journal.ppat.0020113 17040128 10.1371/journal.ppat.0020113 PMC1599764

[54] Baxt LA, Baker RP, Singh U, Urban S. An Entamoeba histolytica rhomboid protease with atypical specificity cleaves a surface lectin involved in phagocytosis and immune evasion. Genes Dev. 2008 6 15;22(12):1636–46. doi: 10.1101/gad.1667708 18559479 10.1101/gad.1667708 PMC2428061

[55] Brossier F, Jewett TJ, Sibley LD, Urban S. A spatially localized rhomboid protease cleaves cell surface adhesins essential for invasion by Toxoplasma. Proc Natl Acad Sci U S A. 2005 3 15;102(11):4146–51. doi: 10.1073/pnas.0407918102 15753289 10.1073/pnas.0407918102 PMC554800

[56] Muller M, Mentel M, van Hellemond JJ, Henze K, Woehle C, Gould SB, et al Biochemistry and evolution of anaerobic energy metabolism in eukaryotes. Microbiol Mol Biol Rev. 2012 6;76(2):444–95. doi: 10.1128/MMBR.05024-11 22688819 10.1128/MMBR.05024-11 PMC3372258

[57] Lantsman Y, Tan KS, Morada M, Yarlett N. Biochemical characterization of a mitochondrial-like organelle from Blastocystis sp. subtype 7. Microbiology. 2008 9;154(Pt 9):2757–66. doi: 10.1099/mic.0.2008/017897-0 18757809 10.1099/mic.0.2008/017897-0

[58] Rotte C, Stejskal F, Zhu G, Keithly JS, Martin W. Pyruvate: NADP+ oxidoreductase from the mitochondrion of Euglena gracilis and from the apicomplexan Cryptosporidium parvum: a biochemical relic linking pyruvate metabolism in mitochondriate and amitochondriate protists. Mol Biol Evol. 2001 5;18(5):710–20. 11319255 10.1093/oxfordjournals.molbev.a003853

[59] Suomi F, Menger KE, Monteuuis G, Naumann U, Kursu VA, Shvetsova A, et al Expression and evolution of the non-canonically translated yeast mitochondrial acetyl-CoA carboxylase Hfa1p. PLoS ONE. 2014 12 11;9(12):e114738 doi: 10.1371/journal.pone.0114738 25503745 10.1371/journal.pone.0114738 PMC4263661

[60] Hamann E, Gruber-Vodicka H, Kleiner M, Tegetmeyer HE, Riedel D, Littmann S, et al Environmental Breviatea harbour mutualistic Arcobacter epibionts. Nature. 2016 6 9;534(7606):254–8. doi: 10.1038/nature18297 27279223 10.1038/nature18297 PMC4900452

[61] Stairs CW, Leger MM, Roger AJ. Diversity and origins of anaerobic metabolism in mitochondria and related organelles. Philos Trans R Soc Lond B Biol Sci. 2015 9 26;370(1678):20140326 doi: 10.1098/rstb.2014.0326 26323757 10.1098/rstb.2014.0326 PMC4571565

[62] Yamaoka S, Hara-Nishimura I. The mitochondrial Ras-related GTPase Miro: views from inside and outside the metazoan kingdom. Front Plant Sci. 2014 7 16;5:350 doi: 10.3389/fpls.2014.00350 25076955 10.3389/fpls.2014.00350 PMC4100572

[63] Vlahou G, Elias M, von Kleist-Retzow JC, Wiesner RJ, Rivero F. The Ras related GTPase Miro is not required for mitochondrial transport in Dictyostelium discoideum. Eur J Cell Biol. 2011 4;90(4):342–55. doi: 10.1016/j.ejcb.2010.10.012 21131095 10.1016/j.ejcb.2010.10.012

[64] Klinger CM, Nisbet RE, Ouologuem DT, Roos DS, Dacks JB. Cryptic organelle homology in apicomplexan parasites: insights from evolutionary cell biology. Curr Opin Microbiol. 2013 8;16(4):424–31. doi: 10.1016/j.mib.2013.07.015 23932202 10.1016/j.mib.2013.07.015 PMC4513074

[65] Perry RJ, Mast FD, Rachubinski RA. Endoplasmic reticulum-associated secretory proteins Sec20p, Sec39p, and Dsl1p are involved in peroxisome biogenesis. Eukaryot Cell. 2009 6;8(6):830–43. doi: 10.1128/EC.00024-09 19346454 10.1128/EC.00024-09 PMC2698310

[66] Gabaldon T. Peroxisome diversity and evolution. Philos Trans R Soc Lond B Biol Sci. 2010 3 12;365(1541):765–73. doi: 10.1098/rstb.2009.0240 20124343 10.1098/rstb.2009.0240 PMC2817229

[67] Philippi ML, Parish RW, Hohl HR. Histochemical and biochemical evidence for the presence of microbodies in phytophthora palmivora. Arch Microbiol. 1975 4 7;103(2):127–32. 1156089 10.1007/BF00436339

[68] Gonzalez NH, Felsner G, Schramm FD, Klingl A, Maier UG, Bolte K. A single peroxisomal targeting signal mediates matrix protein import in diatoms. PLoS ONE. 2011;6(9):e25316 doi: 10.1371/journal.pone.0025316 21966495 10.1371/journal.pone.0025316 PMC3178647

[69] Armbrust EV, Berges JA, Bowler C, Green BR, Martinez D, Putnam NH, et al The genome of the diatom Thalassiosira pseudonana: ecology, evolution, and metabolism. Science. 2004 10 1;306(5693):79–86. doi: 10.1126/science.1101156 15459382 10.1126/science.1101156

[70] Gabaldon T, Ginger ML, Michels PA. Peroxisomes in parasitic protists. Mol Biochem Parasitol. 2016 Sep—Oct;209(1–2):35–45. doi: 10.1016/j.molbiopara.2016.02.005 26896770 10.1016/j.molbiopara.2016.02.005

[71] Kienle N, Kloepper TH, Fasshauer D. Shedding light on the expansion and diversification of the Cdc48 protein family during the rise of the eukaryotic cell. BMC Evol Biol. 2016 10 18;16(1):215 doi: 10.1186/s12862-016-0790-1 27756227 10.1186/s12862-016-0790-1 PMC5070193

[72] Bonifacino JS, Glick BS. The mechanisms of vesicle budding and fusion. Cell. 2004 1 23;116(2):153–66. 14744428 10.1016/s0092-8674(03)01079-1

[73] Schlacht A, Herman EK, Klute MJ, Field MC, Dacks JB. Missing pieces of an ancient puzzle: evolution of the eukaryotic membrane-trafficking system. Cold Spring Harb Perspect Biol. 2014 10 1;6(10):a016048 doi: 10.1101/cshperspect.a016048 25274701 10.1101/cshperspect.a016048 PMC4176009

[74] Schwartz T, Blobel G. Structural basis for the function of the beta subunit of the eukaryotic signal recognition particle receptor. Cell. 2003 3 21;112(6):793–803. 12654246 10.1016/s0092-8674(03)00161-2

[75] Elias M, Patron NJ, Keeling PJ. The RAB family GTPase Rab1A from Plasmodium falciparum defines a unique paralog shared by chromalveolates and rhizaria. J Eukaryot Microbiol. 2009 Jul-Aug;56(4):348–56. doi: 10.1111/j.1550-7408.2009.00408.x 19602080 10.1111/j.1550-7408.2009.00408.x

[76] Kremer K, Kamin D, Rittweger E, Wilkes J, Flammer H, Mahler S, et al An overexpression screen of Toxoplasma gondii Rab-GTPases reveals distinct transport routes to the micronemes. PLoS Pathog. 2013 3;9(3):e1003213 doi: 10.1371/journal.ppat.1003213 23505371 10.1371/journal.ppat.1003213 PMC3591302

[77] Morse D, Webster W, Kalanon M, Langsley G, McFadden GI. Plasmodium falciparum Rab1A Localizes to Rhoptries in Schizonts. PLoS ONE. 2016 6 27;11(6):e0158174 doi: 10.1371/journal.pone.0158174 27348424 10.1371/journal.pone.0158174 PMC4922565

[78] Eme L, Trilles A, Moreira D, Brochier-Armanet C. The phylogenomic analysis of the anaphase promoting complex and its targets points to complex and modern-like control of the cell cycle in the last common ancestor of eukaryotes. BMC Evol Biol. 2011 9 23;11:265,2148-11-265. 10.1186/1471-2148-11-265 PMC3195147 21943402

[79] Harper JW, Burton JL, Solomon MJ. The anaphase-promoting complex: it's not just for mitosis any more. Genes Dev. 2002 9 1;16(17):2179–206. doi: 10.1101/gad.1013102 12208841 10.1101/gad.1013102

[80] Sivakumar S, Gorbsky GJ. Spatiotemporal regulation of the anaphase-promoting complex in mitosis. Nat Rev Mol Cell Biol. 2015 2;16(2):82–94. doi: 10.1038/nrm3934 25604195 10.1038/nrm3934 PMC4386896

[81] Chang L, Zhang Z, Yang J, McLaughlin SH, Barford D. Molecular architecture and mechanism of the anaphase-promoting complex. Nature. 2014 9 18;513(7518):388–93. doi: 10.1038/nature13543 25043029 10.1038/nature13543 PMC4456660

[82] Malik SB, Pightling AW, Stefaniak LM, Schurko AM, Logsdon JM Jr. An expanded inventory of conserved meiotic genes provides evidence for sex in Trichomonas vaginalis. PLoS ONE. 2008 Aug 6;3(8):e2879. 10.1371/journal.pone.0002879 PMC2488364 18663385

[83] Robert T, Nore A, Brun C, Maffre C, Crimi B, Bourbon HM, et al The TopoVIB-Like protein family is required for meiotic DNA double-strand break formation. Science. 2016 2 26;351(6276):943–9. doi: 10.1126/science.aad5309 26917764 10.1126/science.aad5309

[84] Carlton JM, Hirt RP, Silva JC, Delcher AL, Schatz M, Zhao Q, et al Draft genome sequence of the sexually transmitted pathogen Trichomonas vaginalis. Science. 2007 1 12;315(5809):207–12. doi: 10.1126/science.1132894 17218520 10.1126/science.1132894 PMC2080659

[85] Paul MJ, Primavesi LF, Jhurreea D, Zhang Y. Trehalose metabolism and signaling. Annu Rev Plant Biol. 2008;59:417–41. doi: 10.1146/annurev.arplant.59.032607.092945 18257709 10.1146/annurev.arplant.59.032607.092945

[86] Ball S, Colleoni C, Cenci U, Raj JN, Tirtiaux C. The evolution of glycogen and starch metabolism in eukaryotes gives molecular clues to understand the establishment of plastid endosymbiosis. J Exp Bot. 2011 3;62(6):1775–801. doi: 10.1093/jxb/erq411 21220783 10.1093/jxb/erq411

[87] Michel G, Tonon T, Scornet D, Cock JM, Kloareg B. Central and storage carbon metabolism of the brown alga Ectocarpus siliculosus: insights into the origin and evolution of storage carbohydrates in Eukaryotes. New Phytol. 2010 10;188(1):67–81. doi: 10.1111/j.1469-8137.2010.03345.x 20618908 10.1111/j.1469-8137.2010.03345.x

[88] Yoshikawa H, Nagashima M, Morimoto K, Yamanouti Y, Yap EH, Singh M. Freeze-fracture and cytochemical studies on the in vitro cyst form of reptilian Blastocystis pythoni. J Eukaryot Microbiol. 2003 Jan-Feb;50(1):70–5. 12674482 10.1111/j.1550-7408.2003.tb00108.x

[89] Chen XQ, Singh M, Howe J, Ho LC, Tan SW, Yap EH. In vitro encystation and excystation of Blastocystis ratti. Parasitology. 1999 2;118 (Pt 2)(Pt 2):151–60. 10028529 10.1017/s0031182098003667

[90] Zaman V, Howe J, Ng M. Ultrastructure of Blastocystis hominis cysts. Parasitol Res. 1995;81(6):465–9. 7567903 10.1007/BF00931787

[91] Keeling PJ, Burki F, Wilcox HM, Allam B, Allen EE, Amaral-Zettler LA, et al The Marine Microbial Eukaryote Transcriptome Sequencing Project (MMETSP): illuminating the functional diversity of eukaryotic life in the oceans through transcriptome sequencing. PLoS Biol. 2014 6 24;12(6):e1001889 doi: 10.1371/journal.pbio.1001889 24959919 10.1371/journal.pbio.1001889 PMC4068987

[92] Lanuza MD, Carbajal JA, Borras R. Identification of surface coat carbohydrates in Blastocystis hominis by lectin probes. Int J Parasitol. 1996 5;26(5):527–32. 8818733 10.1016/0020-7519(96)00010-0

[93] Crocker PR, Paulson JC, Varki A. Siglecs and their roles in the immune system. Nat Rev Immunol. 2007 4;7(4):255–66. doi: 10.1038/nri2056 17380156 10.1038/nri2056

[94] Eme L, Gentekaki E, Curtis B, Archibald JM, Roger AJ. Lateral Gene Transfer in the Adaptation of the Anaerobic Parasite Blastocystis to the Gut. Curr Biol. 2017 3 20;27(6):807–20. doi: 10.1016/j.cub.2017.02.003 28262486 10.1016/j.cub.2017.02.003

[95] Jarroll EL, Manning P, Berrada A, Hare D, Lindmark DG. Biochemistry and metabolism of Giardia. J Protozool. 1989 Mar-Apr;36(2):190–7. 2657035 10.1111/j.1550-7408.1989.tb01073.x

[96] Anderson IJ, Loftus BJ. Entamoeba histolytica: observations on metabolism based on the genome sequence. Exp Parasitol. 2005 7;110(3):173–7. doi: 10.1016/j.exppara.2005.03.010 15955308 10.1016/j.exppara.2005.03.010

[97] Rossi M, Amaretti A, Raimondi S. Folate production by probiotic bacteria. Nutrients. 2011 1;3(1):118–34. doi: 10.3390/nu3010118 22254078 10.3390/nu3010118 PMC3257725

[98] Muller S, Kappes B. Vitamin and cofactor biosynthesis pathways in Plasmodium and other apicomplexan parasites. Trends Parasitol. 2007 3;23(3):112–21. doi: 10.1016/j.pt.2007.01.009 17276140 10.1016/j.pt.2007.01.009 PMC2330093

[99] Bhattacharya D, Price DC, Chan CX, Qiu H, Rose N, Ball S, et al Genome of the red alga Porphyridium purpureum. Nat Commun. 2013;4:1941 doi: 10.1038/ncomms2931 23770768 10.1038/ncomms2931 PMC3709513

[100] Wisedpanichkij R, Grams R, Chaijaroenkul W, Na-Bangchang K. Confutation of the existence of sequence-conserved cytochrome P450 enzymes in Plasmodium falciparum. Acta Trop. 2011 7;119(1):19–22. doi: 10.1016/j.actatropica.2011.03.006 21510915 10.1016/j.actatropica.2011.03.006

[101] Furlong ST. Sterols of parasitic protozoa and helminths. Exp Parasitol. 1989 5;68(4):482–5. 2656283 10.1016/0014-4894(89)90134-3

[102] Zierdt CH, Donnolley CT, Muller J, Constantopoulos G. Biochemical and ultrastructural study of Blastocystis hominis. J Clin Microbiol. 1988 5;26(5):965–70. 2838509 10.1128/jcm.26.5.965-970.1988 PMC266497

[103] Doyle J.J. and Doyle J.L. A rapid DNA isolation procedure for small quantities of fresh leaf tissue. Phytochemical Bulletin. 1987;19:11.

[104] Rio DC, Ares M Jr, Hannon GJ, Nilsen TW. Purification of RNA using TRIzol (TRI reagent). Cold Spring Harb Protoc. 2010 6;2010(6):pdb.prot5439. 10.1101/pdb.prot5439 20516177

[105] Chevreux B, Pfisterer T, Drescher B, Driesel AJ, Muller WE, Wetter T, et al Using the miraEST assembler for reliable and automated mRNA transcript assembly and SNP detection in sequenced ESTs. Genome Res. 2004 6;14(6):1147–59. doi: 10.1101/gr.1917404 15140833 10.1101/gr.1917404 PMC419793

[106] Grabherr MG, Haas BJ, Yassour M, Levin JZ, Thompson DA, Amit I, et al Full-length transcriptome assembly from RNA-Seq data without a reference genome. Nat Biotechnol. 2011 5 15;29(7):644–52. doi: 10.1038/nbt.1883 21572440 10.1038/nbt.1883 PMC3571712

[107] Boisvert S, Laviolette F, Corbeil J. Ray: simultaneous assembly of reads from a mix of high-throughput sequencing technologies. J Comput Biol. 2010 11;17(11):1519–33. doi: 10.1089/cmb.2009.0238 20958248 10.1089/cmb.2009.0238 PMC3119603

[108] Treangen TJ, Sommer DD, Angly FE, Koren S, Pop M. Next generation sequence assembly with AMOS. Curr Protoc Bioinformatics. 2011 3;Chapter 11:Unit 11.8. 10.1002/0471250953.bi1108s33 PMC3072823 21400694

[109] Boetzer M, Henkel CV, Jansen HJ, Butler D, Pirovano W. Scaffolding pre-assembled contigs using SSPACE. Bioinformatics. 2011 2 15;27(4):578–9. doi: 10.1093/bioinformatics/btq683 21149342 10.1093/bioinformatics/btq683

[110] Wang Z, Hobson N, Galindo L, Zhu S, Shi D, McDill J, et al The genome of flax (Linum usitatissimum) assembled de novo from short shotgun sequence reads. Plant J. 2012 11;72(3):461–73. doi: 10.1111/j.1365-313X.2012.05093.x 22757964 10.1111/j.1365-313X.2012.05093.x

[111] Hu H, Bandyopadhyay PK, Olivera BM, Yandell M. Characterization of the Conus bullatus genome and its venom-duct transcriptome. BMC Genomics. 2011 1 25;12:60,2164-12-60. 10.1186/1471-2164-12-60 PMC3040727 21266071

[112] Simao FA, Waterhouse RM, Ioannidis P, Kriventseva EV, Zdobnov EM. BUSCO: assessing genome assembly and annotation completeness with single-copy orthologs. Bioinformatics. 2015 10 1;31(19):3210–2. doi: 10.1093/bioinformatics/btv351 26059717 10.1093/bioinformatics/btv351

[113] Langmead B, Salzberg SL. Fast gapped-read alignment with Bowtie 2. Nat Methods. 2012 3 4;9(4):357–9. doi: 10.1038/nmeth.1923 22388286 10.1038/nmeth.1923 PMC3322381

[114] Li H, Handsaker B, Wysoker A, Fennell T, Ruan J, Homer N, et al The Sequence Alignment/Map format and SAMtools. Bioinformatics. 2009 8 15;25(16):2078–9. doi: 10.1093/bioinformatics/btp352 19505943 10.1093/bioinformatics/btp352 PMC2723002

[115] Stanke M, Schoffmann O, Morgenstern B, Waack S. Gene prediction in eukaryotes with a generalized hidden Markov model that uses hints from external sources. BMC Bioinformatics. 2006 2 9;7:62 doi: 10.1186/1471-2105-7-62 16469098 10.1186/1471-2105-7-62 PMC1409804

[116] Tempel S. Using and understanding RepeatMasker. Methods Mol Biol. 2012;859:29–51. doi: 10.1007/978-1-61779-603-6_2 22367864 10.1007/978-1-61779-603-6_2

[117] Haas BJ, Delcher AL, Mount SM, Wortman JR, Smith RK Jr, Hannick LI, et al Improving the Arabidopsis genome annotation using maximal transcript alignment assemblies. Nucleic Acids Res. 2003 10 1;31(19):5654–66. doi: 10.1093/nar/gkg770 14500829 10.1093/nar/gkg770 PMC206470

[118] Marchler-Bauer A, Lu S, Anderson JB, Chitsaz F, Derbyshire MK, DeWeese-Scott C, et al CDD: a Conserved Domain Database for the functional annotation of proteins. Nucleic Acids Res. 2011 1;39(Database issue):D225–9. doi: 10.1093/nar/gkq1189 21109532 10.1093/nar/gkq1189 PMC3013737

[119] Bateman A, Coin L, Durbin R, Finn RD, Hollich V, Griffiths-Jones S, et al The Pfam protein families database. Nucleic Acids Res. 2004 1 1;32(Database issue):D138–41. doi: 10.1093/nar/gkh121 14681378 10.1093/nar/gkh121 PMC308855

[120] Abeel T, Van Parys T, Saeys Y, Galagan J, Van de Peer Y. GenomeView: a next-generation genome browser. Nucleic Acids Res. 2012 1;40(2):e12 doi: 10.1093/nar/gkr995 22102585 10.1093/nar/gkr995 PMC3258165

[121] Zhang R, Ou HY, Gao F, Luo H. Identification of Horizontally-transferred Genomic Islands and Genome Segmentation Points by Using the GC Profile Method. Curr Genomics. 2014 4;15(2):113–21. doi: 10.2174/1389202915999140328163125 24822029 10.2174/1389202915999140328163125 PMC4009839

[122] Elhaik E, Graur D, Josic K. Comparative testing of DNA segmentation algorithms using benchmark simulations. Mol Biol Evol. 2010 5;27(5):1015–24. doi: 10.1093/molbev/msp307 20018981 10.1093/molbev/msp307

[123] Dobin A, Davis CA, Schlesinger F, Drenkow J, Zaleski C, Jha S, et al STAR: ultrafast universal RNA-seq aligner. Bioinformatics. 2013 1 1;29(1):15–21. doi: 10.1093/bioinformatics/bts635 23104886 10.1093/bioinformatics/bts635 PMC3530905

[124] Nawrocki EP, Eddy SR. Infernal 1.1: 100-fold faster RNA homology searches. Bioinformatics. 2013 11 15;29(22):2933–5. doi: 10.1093/bioinformatics/btt509 24008419 10.1093/bioinformatics/btt509 PMC3810854

[125] Claros MG, Vincens P. Computational method to predict mitochondrially imported proteins and their targeting sequences. Eur J Biochem. 1996 11 1;241(3):779–86. 8944766 10.1111/j.1432-1033.1996.00779.x

[126] Emanuelsson O, Brunak S, von Heijne G, Nielsen H. Locating proteins in the cell using TargetP, SignalP and related tools. Nat Protoc. 2007;2(4):953–71. doi: 10.1038/nprot.2007.131 17446895 10.1038/nprot.2007.131

[127] Smith AC, Blackshaw JA, Robinson AJ. MitoMiner: a data warehouse for mitochondrial proteomics data. Nucleic Acids Res. 2012 1;40(Database issue):D1160–7. doi: 10.1093/nar/gkr1101 22121219 10.1093/nar/gkr1101 PMC3245170

[128] Eme L, Moreira D, Talla E, Brochier-Armanet C. A complex cell division machinery was present in the last common ancestor of eukaryotes. PLoS ONE. 2009;4(4):e5021 doi: 10.1371/journal.pone.0005021 19352429 10.1371/journal.pone.0005021 PMC2661371

[129] Tonelli FM, Santos AK, Gomes DA, da Silva SL, Gomes KN, Ladeira LO, et al Stem cells and calcium signaling. Adv Exp Med Biol. 2012;740:891–916. doi: 10.1007/978-94-007-2888-2_40 22453975 10.1007/978-94-007-2888-2_40 PMC3979962

[130] Wheeler TJ, Eddy SR. nhmmer: DNA homology search with profile HMMs. Bioinformatics. 2013 10 1;29(19):2487–9. doi: 10.1093/bioinformatics/btt403 23842809 10.1093/bioinformatics/btt403 PMC3777106

[131] Price MN, Dehal PS, Arkin AP. FastTree 2—approximately maximum-likelihood trees for large alignments. PLoS ONE. 2010 3 10;5(3):e9490 doi: 10.1371/journal.pone.0009490 20224823 10.1371/journal.pone.0009490 PMC2835736

[132] Letunic I, Goodstadt L, Dickens NJ, Doerks T, Schultz J, Mott R, et al Recent improvements to the SMART domain-based sequence annotation resource. Nucleic Acids Res. 2002 1 1;30(1):242–4. 11752305 10.1093/nar/30.1.242 PMC99073

[133] Edgar RC. MUSCLE: multiple sequence alignment with high accuracy and high throughput. Nucleic Acids Res. 2004 3 19;32(5):1792–7. doi: 10.1093/nar/gkh340 15034147 10.1093/nar/gkh340 PMC390337

[134] Stamatakis A. RAxML-VI-HPC: maximum likelihood-based phylogenetic analyses with thousands of taxa and mixed models. Bioinformatics. 2006 11 1;22(21):2688–90. doi: 10.1093/bioinformatics/btl446 16928733 10.1093/bioinformatics/btl446

[135] Feyereisen R. Insect CYP Genes and P450 Enzymes In: Insect Molecular Biology and Biochemistry. Elsevier; 2012 p. 236–316.

[136] Nelson DR, Goldstone JV, Stegeman JJ. The cytochrome P450 genesis locus: the origin and evolution of animal cytochrome P450s. Philos Trans R Soc Lond B Biol Sci. 2013 1 6;368(1612):20120474 doi: 10.1098/rstb.2012.0474 23297357 10.1098/rstb.2012.0474 PMC3538424

[137] Fischer M, Knoll M, Sirim D, Wagner F, Funke S, Pleiss J. The Cytochrome P450 Engineering Database: a navigation and prediction tool for the cytochrome P450 protein family. Bioinformatics. 2007 8 1;23(15):2015–7. doi: 10.1093/bioinformatics/btm268 17510166 10.1093/bioinformatics/btm268

[138] Sirim D, Wagner F, Lisitsa A, Pleiss J. The cytochrome P450 engineering database: Integration of biochemical properties. BMC Biochem. 2009 11 12;10:27,2091-10-27. 10.1186/1471-2091-10-27 PMC2779185 19909539

[139] Aurrecoechea C, Brestelli J, Brunk BP, Fischer S, Gajria B, Gao X, et al EuPathDB: a portal to eukaryotic pathogen databases. Nucleic Acids Res. 2010 1;38(Database issue):D415–9. doi: 10.1093/nar/gkp941 19914931 10.1093/nar/gkp941 PMC2808945

[140] Eddy SR. A probabilistic model of local sequence alignment that simplifies statistical significance estimation. PLoS Comput Biol. 2008 5 30;4(5):e1000069 doi: 10.1371/journal.pcbi.1000069 18516236 10.1371/journal.pcbi.1000069 PMC2396288

[141] Posada D. jModelTest: phylogenetic model averaging. Mol Biol Evol. 2008 7;25(7):1253–6. doi: 10.1093/molbev/msn083 18397919 10.1093/molbev/msn083

[142] Le SQ, Gascuel O. An improved general amino acid replacement matrix. Mol Biol Evol. 2008 7;25(7):1307–20. doi: 10.1093/molbev/msn067 18367465 10.1093/molbev/msn067

[143] Lartillot N, Lepage T, Blanquart S. PhyloBayes 3: a Bayesian software package for phylogenetic reconstruction and molecular dating. Bioinformatics. 2009 9 1;25(17):2286–8. doi: 10.1093/bioinformatics/btp368 19535536 10.1093/bioinformatics/btp368

[144] Guindon S, Dufayard JF, Lefort V, Anisimova M, Hordijk W, Gascuel O. New algorithms and methods to estimate maximum-likelihood phylogenies: assessing the performance of PhyML 3.0. Syst Biol. 2010 5;59(3):307–21. doi: 10.1093/sysbio/syq010 20525638 10.1093/sysbio/syq010

[145] Eddy SR. A new generation of homology search tools based on probabilistic inference. Genome Inform. 2009 10;23(1):205–11. 20180275

[146] Nito K, Kamigaki A, Kondo M, Hayashi M, Nishimura M. Functional classification of Arabidopsis peroxisome biogenesis factors proposed from analyses of knockdown mutants. Plant Cell Physiol. 2007 6;48(6):763–74. doi: 10.1093/pcp/pcm053 17478547 10.1093/pcp/pcm053

[147] Cantarel BL, Coutinho PM, Rancurel C, Bernard T, Lombard V, Henrissat B. The Carbohydrate-Active EnZymes database (CAZy): an expert resource for Glycogenomics. Nucleic Acids Res. 2009 1;37(Database issue):D233–8. doi: 10.1093/nar/gkn663 18838391 10.1093/nar/gkn663 PMC2686590

[148] Eddy SR. Profile hidden Markov models. Bioinformatics. 1998;14(9):755–63. 9918945 10.1093/bioinformatics/14.9.755

[149] Eisenhaber B, Schneider G, Wildpaner M, Eisenhaber F. A sensitive predictor for potential GPI lipid modification sites in fungal protein sequences and its application to genome-wide studies for Aspergillus nidulans, Candida albicans, Neurospora crassa, Saccharomyces cerevisiae and Schizosaccharomyces pombe. J Mol Biol. 2004 3 19;337(2):243–53. doi: 10.1016/j.jmb.2004.01.025 15003443 10.1016/j.jmb.2004.01.025

[150] Kall L, Krogh A, Sonnhammer EL. A combined transmembrane topology and signal peptide prediction method. J Mol Biol. 2004 5 14;338(5):1027–36. doi: 10.1016/j.jmb.2004.03.016 15111065 10.1016/j.jmb.2004.03.016

[151] Horton P, Park KJ, Obayashi T, Fujita N, Harada H, Adams-Collier CJ, et al WoLF PSORT: protein localization predictor. Nucleic Acids Res. 2007 7;35(Web Server issue):W585–7. doi: 10.1093/nar/gkm259 17517783 10.1093/nar/gkm259 PMC1933216

[152] Krogh A, Larsson B, von Heijne G, Sonnhammer EL. Predicting transmembrane protein topology with a hidden Markov model: application to complete genomes. J Mol Biol. 2001 1 19;305(3):567–80. doi: 10.1006/jmbi.2000.4315 11152613 10.1006/jmbi.2000.4315

[153] Hodges ME, Wickstead B, Gull K, Langdale JA. Conservation of ciliary proteins in plants with no cilia. BMC Plant Biol. 2011 12 30;11:185,2229-11-185. 10.1186/1471-2229-11-185 PMC3268115 22208660

[154] Katoh K, Standley DM. MAFFT: iterative refinement and additional methods. Methods Mol Biol. 2014;1079:131–46. doi: 10.1007/978-1-62703-646-7_8 24170399 10.1007/978-1-62703-646-7_8

[155] Castresana J. Selection of conserved blocks from multiple alignments for their use in phylogenetic analysis. Molecular biology and evolution. 2000;17:540 10742046 10.1093/oxfordjournals.molbev.a026334

