# Supplementary material for: Extreme genome diversity in the hyper-prevalent parasitic eukaryote Blastocystis
Source: PLoS Biol. 2017 Sep 11;15(9):e2003769. doi: 10.1371/journal.pbio.2003769 (PMC5608401; doi:10.1371/journal.pbio.2003769)
Supplement: S1 Text — (DOCX) [file pbio.2003769.s001.docx]

**Supplemental Information**

**Segmental duplications versus whole genome duplication**

The distribution of allelic frequencies for 6,390 SNPs across the genome of ST1 (S5 Fig) revealed a consistent diploid signal of 50% suggesting that the scaffolds are disomic. The contention that ST1 is diploid is further strengthened by examination of read depth coverage. Most of the scaffolds longer than 10,000 bps exhibit a highly similar median read depth (S6 Fig) value of 113. If a read depth of 113 is scaled so that a normal diploid state is 1 then 0.5 would represent monosomic scaffolds, 1.5 trisomic regions and 2 tetrasomic portions of the genome (S6 Fig). ST1 has a small number of scaffolds at or near 0.5 suggesting that there may be chromosomes or at least portions that are present in a single copy. Further examination of these monosomic scaffolds revealed, however, that unlike most of the genome for which both copies have been assembled together in the same scaffold (as evinced by the SNP allelic frequencies) these portions have remained separate due either to assembly artifacts or the presence of sufficient differences to make assembly problematic. Typically the monosomic scaffolds have a counterpart in the scaffolds with equally low read depth coverage.

*Blastocystis* ST1 also has a few scaffolds that have a scaled median read depth of 1.5 (trisomic) and 2 (tetrasomic) as well as a greater number with intermediate states. Examination of read depth coverage of individual scaffolds illustrates that these various ploidy states are real rather than an artifact of assembly or read pair mapping. S7 Fig depicts the read depth coverage of scaffold 113 that has a median value of 213 (1.88 scaled). The region between 30,000 and 68,000 has a consistently high read depth coverage approximately twice the typical value suggesting that there are four copies of this region in the genome. Similarly, scaffold 102, a "typical" scaffold with a median read depth of 113 (1 scaled) has a consistent disomic signal for most of its length (5,000-36,000) (S8 Fig). Between 3,000 and 5,000, however, the read depth spikes. The 27 SNPs across this region of elevated coverage do not exhibit a disomic signal of 50% allelic frequencies but instead have ratios indicative of 3-6 copies. The region 1-3,000 conversely has a read depth coverage of ~50. This region of low coverage matches a region in scaffold 44 that, if combined with it, would give a read depth close to that of the typical disomic regions.

It is unclear whether *Blastocystis* ST1 has duplicated or missing whole chromosomes due to the fragmented state of the genome assembly. Recent investigations of chromosome and gene copy number in *Leishmania* strains [1, 2] have suggested that aneuploidy can be an adaptive mechanism particularly for asexual, unicellular parasites [3]. The genotypic/chromosomal plasticity can be present within a single cell or between cells resulting in populations with different chromosome numbers. This mosaicism of cells with monosomic, disomic or trisomic chromosomes has been linked to intermediate levels of read depth coverage [2] in the *Leishmania* studies. Genome projects typically involve the sequencing of hundreds or thousands of cells that are presumed to be identical in their genomic content.

Clearly ST1, given that some scaffolds have long non-disomic stretches, has experienced some level of genomic duplication. These findings are consistent with the presence of duplicated genomic blocks in ST7 [4]. Most of the scaffolds with non-disomic type read depth coverage are less than 20,000 bps (S8 Fig). Moreover, as seen from scaffold 102 (S8 Fig) and 113 (S7 Fig) some of the scaffolds contain elevated stretches of high read depth coverage of varying length. This mosaicism would suggest segmental duplication rather than whole chromosome copies.

**Telomeres**

Telomerase is a ribonucleoprotein complex that adds a repeat sequence to the 3' end of telomeres. In humans, telomerase consists of the telomerase reverse transcriptase (TERT), a noncoding telomerase RNA (TERC) and dyskerin (DKC1) [5]. *Blastocystis* STs 1, 4 and 7 each have a single copy of TERT while ST1 has three copies of DKC1, ST4 has one copy of DKC1 and ST7 has two copies (S4 Table). STs 1, 4 and 7 also have two copies each of Est1 that stimulates DNA extension activity [6].

A search for telomere repeats in ST1 identified 17 possible sites (S5 Table). Of these, 13 were found at scaffold boundaries indicating that they are terminal repeats typically associated with telomeres. The remaining four sites were found in interior regions and of these one was bounded by a 700 bp gap suggesting that its internal placement might be an assembly artifact. The repeat motif identified was TTAGGG, which has been identified in other stramenopiles including *Blastocystis* ST7 and is possibly ancestral in eukaryotes [7]. The number of motifs ranged from 15 to 68 with most in the range of 20 to 40. Two of the 17 regions also had small runs of TTAGG adjacent to them. A similar search in ST7 identified nine possible telomere regions and confirmed the original finding of TTAGGG [7] as the repeat sequence. No telomere repeats were detected in the genome sequences available for ST4. The lack of telomere repeats in ST4 is likely an assembly artifact since repetitive regions are difficult to assemble and are often discarded.

**Ribosomal protein genes**

Eukaryotic ribosomes are composed of the 40S small subunit and the 60S large subunit with each subunit made up of RNA and many individual proteins. The four ribosomal RNAs and ~80 ribosomal proteins account for about 80% of the RNA and 5-10% of the protein in a cell [8]. Multiple copies of rRNA genes are usually present in eukaryotic genes either as single genes or in tandem arrays. Vital genes may also be highly expressed to accommodate the need for large quantities of a particular protein. Both of these methods appear to be used by *Blastocystis* to produce an abundance of ribosomal proteins.

Multiple copies of many ribosomal proteins is an uncommon genomic feature that seems mainly restricted to green plants. According to the Ribosomal Protein Gene Database the diatom *Phaeodactylum tricornutum* has two copies of RPS20, RPS29, RPL36A and RPLP2 while the rest of the ribosomal proteins are restricted to having one gene [9]. Another diatom, *Thalassiosira pseudonana*, has only one ribosomal protein, RPL23, that has more than one genomic copy, as does the red alga *Cyanidioschyzon merolae* (RPS13). Conversely *Arabidopsis thaliana* has at least two copies and often three or more of every single ribosomal protein as does *Glycine max*. Most of the genes in *Oryza sativa* have two or more copies. Among green algae *Chlamydomonas reinhardtii* does not have any additional copies of ribosomal protein genes while *Volvox* *carteri* has only one (RPS20). The parasites *Plasmodium falciparum*, *Toxoplasma gondii* and *Giardia lamblia* do not have additional copies of ribosomal protein genes [9].

Hence, it is interesting to note that all of the 25 subunits of the 40S subunit have multiple copies in ST1 (S6 Table). ST1 has more copies than ST7 for 15 of the 25 genes while ST7 has three genes with more copies than ST1. ST7 also has four genes for which there is a single copy. The ribosomal protein S23 is illustrative of the differences seen between ST1 and ST7 in 40S ribosomal proteins. ST1 has six copies (S9 Fig) while ST7 has three. All of the 145 amino acids are the same in the ST1 copies except for the gene AV274_4999 that differs in three residues. At the nucleotide level all six copies are slightly different from each other with identities ranging from 96% (415/434) to 99% (429/434). Moreover, the flanking gene sequences differ between the copies. Several of the copies have the same flanking sequence (AV274_4999 and AV274_3189 both have a flanking PGP synthase gene) but the other flanking gene is different indicating that the copies are independent and real rather than being artifacts of the assembly. The three ST7 genes also differ in their flanking genes but have counterparts with ST1 in terms of gene order. CBK19643 shares its flanking genes of 5'-3' exoribonuclease and RPL27 with ST1 gene AV274_3253 while CBK23735 corresponds to AV274_3189 and CBK24083 matches AV274_4759 in gene order (S9 Fig). The ST1 gene arrangements for AV274_5206 and AV274_5757 suggest that a copy of the RS23 gene has been inserted since ST7 has the same flanking genes but without the intervening RS23 gene. The final ST1 copy, AV274_4999, does not share its gene arrangement with ST7. One of its flanking genes, PGP synthase, is shared with CBK23735 but the other ST1 flanking gene, a 26S proteasome regulatory subunit, is not found near a RS23 gene in ST7. The corresponding gene in ST7 is CBK23077. As with many other genomic regions this is indicative of substantial duplication/rearrangement events. The ST1 RS23 genes do not appear to be processed pseudogenes since they all possess at least two introns with matching RNAseq data and no indication of a polyA insertion near the 3' end. Nor do they appear to be pseudogenes derived from decayed duplications.

A similar pattern of more multiple copies in ST1 than in ST7 is seen for the ribosomal proteins of the 60S subunit (S7 Table). Of the 45 large subunit protein genes identified for ST1, 44 had more than one copy while in ST7 all 44 identified genes had multiple copies. Twenty of the genes had more copies in ST1, seven had more copies in ST7 and 18 had the same number in the two subtypes. The percentage of genes with more copies in a particular subtype is higher for 40S genes. 60% of the ST1 genes had more copies than ST7 for 40S genes versus 44% for 60S genes. Similarly, 28% of the ST7 genes had more copies of 40S genes than for 60S genes (15%).

Why does *Blastocystis* possess multiple copies of almost all the ribosomal protein genes? The widespread presence of processed pseudogenes appears to be a mammalian phenomenon and does not match the characteristics of the ribosomal protein genes in *Blastocystis*. It is likely that the copies arose through multiple cases of segmental duplication. Perhaps they have not had sufficient time to decay to pseudogenes. Or, as in the case of green plants, having multiple copies of these vital genes has been found useful. *Blastocystis* does not have different tissues so the copies cannot be used for differential tissue expression. Any extraribosomal functions that these copies might possess are unknown. Perhaps the most plausible explanation is that an abundance of the building blocks of ribosomes allows this unicellular organism to take advantage of favorable growth conditions by rapidly ramping up protein production.

**Membrane trafficking System**

Overall, much of the membrane trafficking machinery is conserved in *Blastocystis* STs 1, 4 and 7. However, one conspicuous absence is in the Endosomal Sorting Complexes Required for Transport (ESCRTs) (S3 Fig). The ESCRTs are a set of five protein complexes that function together as a coat in the endocytic system, and are responsible for inward budding of endosomes to create a multi-vesicular body. No homologs could be identified for any of the four proteins that make up the ESCRT I complex, which is responsible for cargo sequestration and recruiting the ESCRT II complex. The other ESCRT complexes, with the exception of ESCRT 0 (known to be restricted to the Opisthokonta [10]), are largely complete, and some have multiple subunit paralogs. Additionally, a homolog of Tom1esc was found, a protein that has been implicated as an alternative ESCRT 0 in non-opisthokonts, due to its domain structure. It has previously been shown that although Tom1esc has a wide distribution across the tree of eukaryotes [11] and therefore was likely present in the Last Eukaryotic Common Ancestor (LECA), it is patchily distributed. Until now, Tom1esc was only identified in two members of the SAR clade, and its new-found presence in *Blastocystis* provides further evidence of its pan-eukaryotic distribution.

*Blastocystis* ST1 has a somewhat expanded set of adaptin machinery, the adaptor proteins that are involved in vesicle budding. Approximately half of the adaptin subunits have two or more paralogs; all are significantly different at the nucleotide level, and none of the sequences are fragments. There is also a large expansion of Sec24, a member of the COP II coat complex: seven paralogs in ST1, four in ST4, and four in ST7.

*ArfGAPs*

Arf GAPs act as regulators for the small GTPase Arf by stimulating the hydrolysis of GTP, and have also recently been proposed to act as Arf effectors. Of the 11 known Arf GAP subfamilies, eight (ArfGAP1, ArfGAP2/3, SMAP, ACAP, AGAP, ADAP, AGFG, and ArfGAPC2) are proposed to have been present in the LECA, whereas, three (ASAP, ARAP, and GIT) are found only within the Opisthokonta [10]. Of the eight ancient Arf GAP subfamilies, SMAP, ArfGAP1, ArfGAP2/3, ACAP, and AGFG are infrequently lost, and thus represent a core set of Arf GAPs in the LECA. In ST1, ST4 and ST7 at least one copy of four of these five ArfGAP subfamilies was found (S3 Fig). All three subtypes are missing ACAP, AGAP, ADAP, and ArfGAPC2, consistent with previous results showing multiple instances of secondary loss of these subfamilies.

*Tethering Factors*

HOPS

The HOPS complex is made up of six components: Vps11, Vps16, Vps18, Vps33, Vps39, and Vps41. At least one copy of each subunit was identified in STs 1, 4 and 7. Although it is expected that the complex is completely conserved, there are more paralogs in *Blastocystis* than in other stramenopiles such as *Phytophthora infestans*.

COG

The COG complex is made up of eight subunits, COG1-8, all of which are presumed to have been present in the LECA. STs 1, 4 and 7 have all the subunits, including two copies of COG8.

GARP

GARP is composed of four subunits: Vps51, Vps52, Vps53, and Vps54. In the three subtypes an entire complex was identified with multiple copies of some subunits. Specifically, two copies of Vps51 in ST1 and ST7 were found, but only a single copy in ST4; all three subtypes possess two copies of Vps52, while ST1 has three copies of Vps53. The presence of a complete complex is expected, as this is the case with *P. infestans*. However, the overall number of paralogs is larger than expected.

Exocyst

The exocyst complex is made up of eight components: Sec3, Sec5, Sec6, Sec8, Sec10, Sec15, Exo70, and Exo84. All three subtypes had one copy of each subunit.

Dsl1

Dsl1 is composed of three subunits: Sec39, Tip20, and Dsl1. Only a single copy of Tip20 was identified in all three subtypes. This is unexpected as all three subunits were expected to be present in the LECA, as well as in stramenopiles generally. This likely represents gene divergence, but could also represent genuine loss, as this is the case with parasites in the Apicomplexa [12].

TRAPP

There are two forms of the TRAPP complex: TRAPPI, which is composed of Bet3, Bet5, Trs20, Trs23, Trs31, Trs33, and Trs85. TRAPPII is composed of TRAPPI in addition to Trs65, Trs120, and Trs130. Subtypes 1, 4 and 7 have one copy each of the TRAPPI subunits except for Trs33 which is missing from all three. ST1 has two copies of Trs120 while ST4 and ST7 have a single copy.

*SM Proteins*

Sec/Munc-18 (SM) proteins regulate *trans* SNARE-SNARE interactions during the fusion of a vesicle with its target membrane. Four SM proteins exist, each with its own sub-cellular localization. Sly1 is active primarily in the early secretory system between the ER and the Golgi complex, Vps33 acts at late endosomes, Vps45 acts at the *trans*-Golgi Network, and Sec1 acts at the plasma membrane. At least one of each SM protein is expected to have been present in the Last Eukaryotic Common Ancestor. Subtypes 1, 4 and 7 of *Blastocystis* possess a single copy of Sly1, Vps45, and Sec1, and have four copies of Vps33. Additionally, STs 1, 4 and 7 possess one SM protein that was unable to be classified even with phylogenetic trees. This likely represents an additional divergent paralog of one of the other known SM proteins.

*SNAREs*

Qa-SNAREs

Six Qa-SNAREs are predicted to have been present in the LECA, including SynPM, SynE, Syn5, Syn16, Syn17, and Syn18. Two genes encoding Syn5 and one gene encoding Syn16 were identified in all three subtypes, as were three SNAREs that were unable to be classified, beyond being Qa-SNAREs. Further analysis will be required to assign orthology to these sequences.

Qb-SNAREs

Four Qb-SNAREs are thought to have been present in the LECA: Vti1, Bos1, Sec20, and Gos1. Except for Sec20, ST1, ST4 and ST7 had one copy each as well as an unclassified Qb-SNARE.

*Qc-SNAREs*

All three subtypes have a single copy of Syn6/10 and Syp7, with two copies of Bet1 and three unclassified Qc SNAREs. Use1 was not clearly identified in any of the three subtypes.

R-SNAREs

Four R-SNAREs are expected to have been present in the LECA: Sec22, VAMP7, Ykt6, and Tomosyn. The three subtypes had one copy each of Sec22, Ykt6 and three copies of VAMP7.

**Cell cycle, DNA repair and meiosis**

Cell cycles in eukaryotes are regulated by the TORC1 complex, and the transition from mitosis to meiosis occurs in response to amino acid limitation through the down-regulation of the TORC1 complex (i.e. TOR, RPTOR and LTS8) by Ilm1/GATOR1 regulators (i.e. Iml1, Npr2, Npr3) [13, 14]. *Blastocystis* STs 1, 4 and 7 have all the elements of the TORC1 complex but lack the Iml1 regulator. Meiosis can also be induced in fission yeast by the interaction of the Mei2p protein with the raptor homolog Mip1p [15, 16]. Although Mei2-like genes are not meiosis specific [17], the analyses detected a family expansion of Mei2-like genes with four paralogs, allowing for a hypothetical initiation of meiosis in a fashion similar to that observed in fission yeast.

Homologs of MutYH and Apex2, involved in base excision repair (BER), are present (S6 Data). Homologs of many of the core nucleotide excision repair (NER) protein-coding genes are also present except for Rad4, Rad23, Rad14, TFB1, TFB3 and Rad28. Homologs of conserved DNA damage response genes Suc1, Phr1 and the MCM gene family (involved in licensing and DNA replication) are present, as are the DNA damage checkpoint genes Rad9, Rad1, Mec3, Rad27, Rad53, Mec1 and Tel1. Homologs of DNA polymerase subunits involved in repair are also identified (delta, epsilon, PCNA, zeta and Rev1), while subunits beta, gamma, kappa and Rev7 are absent. Components of the post-replication repair Rad6 pathway (Rad6, Smc5 and Smc6) are present, as well as homologs of editing and processing nucleases (Fen1, Exo1, Spo11-2 and Top6BL). Chromatin structural components such as ATM (Tel1), RecQ helicases and condensins (Smc1 and Smc4) are also identified. Genes encoding essential mismatch repair (MMR) proteins, the MutS and MutL homologs Msh2, Msh6, Mlh1 and Pms1, which typically act together in meiotic crossovers and MMR, are all present, but MMR protein Msh3 is missing. Components of the homologous recombination (HR) machinery are encoded: Rad51, Rad55 (Xrcc2), Rad57 (Xrcc3), Dmc1, Hop2, Mnd1, Rad52, Rdh54, Brca2, Rad50, Mre11, Rad21 and the synaptonemal complex (SC) axial element protein, Hop1. The presence of genes encoding components of HR and not NHEJ suggests that HR is the principal mechanism for DNA double-strand break repair in *Blastocystis*, similar to *Trichomonas vaginalis* [18].

The *Blastocystis* ST1 genome contains most “core meiosis genes” [19] (S6 Data and S4 Fig), including the meiosis-related orthologs of Hop1, Spo11-2, Top6BL, Hop2, Mnd1, Dmc1, Mer3, Msh4 and Msh5. The presence of Spo11-2 but not Spo11-1 or Spo11-3 in *Blastocystis* (ST1, ST4 and ST7) is consistent with other stramenopiles and may indicate that meiotic recombination initiation could rely on a Spo11-2 homodimer [20] paired with topoVIB-like to form the meiosis-specific transesterase that cleaves double stranded DNA. Consistent with findings in other stramenopiles, alveolates, Amoebozoa and excavates [20], *Blastocystis* appears to lack Rec8 that encodes the meiosis-specific sister chromatid cohesin subunit in animals, fungi and plants. Sister chromatid cohesion and chromosome segregation during meiosis can occur without Rec8 [21, 22]. However, there is an expansion of the general cohesin subunit Rad21 (three paralogs in ST1, two in ST4, and none in ST7). Such expansions have been implicated in the emergence of different meiotic roles in mammals (i.e.RAD21-L) [23], as well as in mitosis in plants [24]. SCC3 was found in ST1 and ST4. In addition, *Blastocystis* clearly encodes meiosis-specific interhomolog recombination proteins Dmc1, Hop2 and Mnd1, which typically act together in organisms that have them. *Blastocystis* probably has randomly distributed meiotic crossovers, since it encodes the Mus81-Mms4 nuclease, in addition to SC-associated meiotic crossovers sensitive to positional interference (CI) that are mediated by the “ZMM” complex [25, 26], illustrated in S4 Fig. Mer3, Msh4 and Msh5 were found, a subset of ZMM proteins that are implicated in CI and SC assembly, and an SC axial element protein, Hop1 (S4 Fig). CI requires assemblage of the SC transverse elements, Zip1, Zip2, Zip3, and SYCP2, which could not be identified. These are known for sharing little or no identity with their functional homologs in other taxa [27, 28], so the inability to identify SC transverse elements in *Blastocystis* does not directly imply that meiosis lacks the SC. The presence of transcriptionally active SC-specific Mer3 and Hop1 genes, however, seem to indicate the SC. Evidence of active transcription was found for all of the detected DNA repair and meiosis genes in ST1. However, the transcription levels were low for these genes suggesting that it was not very common during the culture conditions from which *Blastocystis* ST1 RNA was prepared.

The gamete membrane protein HAP2 (essential for gamete fusion) [29, 30] was not identified. However, GEX1, implicated in nuclear fusion during sexual reproduction [30] and early embryogenesis [31, 32] was found. Both HAP2 and GEX1 can apparently be traced back to the last eukaryotic common ancestor (LECA) [33] and the analyses seem to suggest that if present, HAP2 is highly divergent from the currently known orthologs.

**Cytokinesis**

Among the homologs of proteins suspected to be involved in cytokinesis, *Blastocystis* ST1 has 58 components that are thought to be present in the ancestor of stramenopiles and conserved in most lineages [34] (S9 Data). Half of the missing components are directly or indirectly dependent on calcium (Ca^2+^): cam kinase II, calcyclin binding protein, annexins V, VI and VII, phospholipase C β 2 and G protein β-2. Based on this observation, the presence of the main known families of calcium-binding proteins (CBP) was further investigated [35]. Here again, analyses show that while *Blastocystis* has not lost all CBPs, more than half of the families found in other stramenopiles (and most eukaryotes) are absent in *Blastocystis* ST1 (S10 Data). Altogether, these results suggest a possible change in calcium-dependent signaling pathways and in the cellular role of this ion in this organism.

**Metabolism of carbohydrate active enzymes (CAZymes)**

A total of 203 CAZymes were identified in the *Blastocystis* ST1 genome: 49 glycosyl hydrolases (GHs), 132 glycosyl transferases (GTs), three carbohydrate esterases (CEs), 18 carbohydrate-binding modules (CBMs) and a single polysaccharide lyase (PL) (S8 Table). The PL9, which is distantly related to pectate lysases, is not present in other stramenopiles except for *Ectocarpus siliculosus*. *Blastocystis* contains some CAZy families absent from stramenopiles but well conserved in other taxonomic groups. These include GH20, GH29 and GH99 that are likely involved in N-glycan processing. The carbohydrate-active enzyme diversity is extremely variable in eukaryotes and the number of the various CAZy families in stramenopiles is apparently not related to their lifestyles (S9 Table). *Blastocystis*, with a reduced genome and parasitic lifestyle, has as many GH and GT families as the free-living diatom *T. pseudonana* and the macroalga *E. siliculosus*. It should be noted that a large number of the predicted *Blastocystis* CAZymes show weak sequence similarity to experimentally characterized CAZymes, preventing accurate functional predictions.

**Flagellar Proteins**

*Blastocystis* has never been described as possessing any flagellum [4]. Nevertheless, we interrogated the protein data set from ST1 for the presence of flagellar proteins. Hodges et al. [36] identified a core set of 213 proteins found in organisms with cilia and generally not found in organisms without ciliated stages.

A reciprocal best blast approach of proteins from *Chlamydomonas reinhardtii* found six orthologs from *Blastocystis* ST1 (S10 Table) that were part of the 213 conserved set of flagellar proteins. In contrast, *Phytopthora sojae*, which does have a flagellated stage, had 157 orthologs [36] to the *C. reinhardtii* 213 flagellar protein set.

**GC content**

The GC content of the three *Blastocystis* subtypes 1,4 and 7 is remarkable divergent, ranging from 39.6% in ST4 to 54.6% in ST1. A number of hypotheses have been put forward to explain the variability of GC content across the tree of life as well as GC heterogeneity in a single genome. One of the proposed rationales for differences in GC content, particularly among bacteria, is that there is selection for AT rich genomes among obligatory pathogens and symbionts [37] because GTP and CTP nucleotides are energetically more costly than ATP and UTP. The differences would be most pronounced when comparing GC content between free-living and non free-living relatives. Such a mechanism is unlikely to be at play in the case of *Blastocystis* subtypes 1,4 and 7 since they are all obligatory parasites inhabiting similar hosts and environments, frequently being found together. Nor does the phylogenetic evidence suggest that ST1, with its higher GC content, has been a parasite for less time than the other two subtypes and thus experiencing less selection against a high GC content.

Plots of the GC content across the individual genomes reveal ST1 and particularly ST4 to have fairly homogenous distributions (S10 Fig). Except for one small spike (89 Kb in length) the GC content fluctuations of ST1 stay within two percentage points of its average GC content of 54.6. ST4 is even more stable with two long GC similar segments within 2.5 percentage points of each other. ST7, on the other hand, has 12 GC similar segments that deviate at least 3 percentage points from the average value of 45.2. Why ST7 should have more compositionally homogeneous domains than ST1 and ST4 is at present unclear. One explanation for the genomic landscape of GC content is biased gene conversion [38]. This model predicts that frequent chromosomal rearrangements will lead to a homogenization of GC content. Given ST4's paucity of compositionally homogeneous domains one might speculate that its rate of recombination is higher than that of ST7 and similar across its chromosomes.

**Average level of heterozygosity**

The average level of heterozygosity for ST1 was calculated by examining the reads that mapped to each site in the genome and determining how many sites had more than one possible base type present. To be considered relevant the site had to have a read depth coverage of at least 10. This left 15,890,360 sites. Heterozygous sites were those for which at least two different base types were present with at least 2 (or 3) reads containing that base. Using at least 2 reads per possible base type as the cutoff, 48,098 sites were deemed heterozygous for an average level of 0.00302 while a cutoff of 3 reads gave an average heterozygosity value of 0.00243.

References

1. Sterkers Y, Lachaud L, Bourgeois N, Crobu L, Bastien P, Pages M. Novel insights into genome plasticity in Eukaryotes: mosaic aneuploidy in Leishmania. Mol Microbiol. 2012 Oct;86(1):15-23.

2. Rogers MB, Hilley JD, Dickens NJ, Wilkes J, Bates PA, Depledge DP, et al. Chromosome and gene copy number variation allow major structural change between species and strains of Leishmania. Genome Res. 2011 Dec;21(12):2129-42.

3. Mannaert A, Downing T, Imamura H, Dujardin JC. Adaptive mechanisms in pathogens: universal aneuploidy in Leishmania. Trends Parasitol. 2012 Sep;28(9):370-6.

4. Denoeud F, Roussel M, Noel B, Wawrzyniak I, Da Silva C, Diogon M, et al. Genome sequence of the stramenopile Blastocystis, a human anaerobic parasite. Genome Biol. 2011;12(3):R29,2011-12-3-r29. Epub 2011 Mar 25.

5. Cohen SB, Graham ME, Lovrecz GO, Bache N, Robinson PJ, Reddel RR. Protein composition of catalytically active human telomerase from immortal cells. Science. 2007 Mar 30;315(5820):1850-3.

6. DeZwaan DC, Freeman BC. The conserved Est1 protein stimulates telomerase DNA extension activity. Proc Natl Acad Sci U S A. 2009 Oct 13;106(41):17337-42.

7. Fulneckova J, Sevcikova T, Fajkus J, Lukesova A, Lukes M, Vlcek C, et al. A broad phylogenetic survey unveils the diversity and evolution of telomeres in eukaryotes. Genome Biol Evol. 2013;5(3):468-83.

8. Zhang Z, Harrison P, Gerstein M. Identification and analysis of over 2000 ribosomal protein pseudogenes in the human genome. Genome Res. 2002 Oct;12(10):1466-82.

9. Nakao A, Yoshihama M, Kenmochi N. RPG: the Ribosomal Protein Gene database. Nucleic Acids Res. 2004 Jan 1;32(Database issue):D168-70.

10. Schlacht A, Herman EK, Klute MJ, Field MC, Dacks JB. Missing pieces of an ancient puzzle: evolution of the eukaryotic membrane-trafficking system. Cold Spring Harb Perspect Biol. 2014 Oct 1;6(10):a016048.

11. Herman EK, Walker G, van der Giezen M, Dacks JB. Multivesicular bodies in the enigmatic amoeboflagellate Breviata anathema and the evolution of ESCRT 0. J Cell Sci. 2011 Feb 15;124(Pt 4):613-21.

12. Klinger CM, Klute MJ, Dacks JB. Comparative genomic analysis of multi-subunit tethering complexes demonstrates an ancient pan-eukaryotic complement and sculpting in Apicomplexa. PLoS One. 2013 Sep 27;8(9):e76278.

13. Wei Y, Reveal B, Reich J, Laursen WJ, Senger S, Akbar T, et al. TORC1 regulators Iml1/GATOR1 and GATOR2 control meiotic entry and oocyte development in Drosophila. Proc Natl Acad Sci U S A. 2014 Dec 30;111(52):E5670-7.

14. Dokudovskaya S, Rout MP. SEA you later alli-GATOR--a dynamic regulator of the TORC1 stress response pathway. J Cell Sci. 2015 Jun 15;128(12):2219-28.

15. Shinozaki-Yabana S, Watanabe Y, Yamamoto M. Novel WD-repeat protein Mip1p facilitates function of the meiotic regulator Mei2p in fission yeast. Mol Cell Biol. 2000 Feb;20(4):1234-42.

16. Jeffares DC, Phillips MJ, Moore S, Veit B. A description of the Mei2-like protein family; structure, phylogenetic distribution and biological context. Dev Genes Evol. 2004 Mar;214(3):149-58.

17. Anderson GH, Hanson MR. The Arabidopsis Mei2 homologue AML1 binds AtRaptor1B, the plant homologue of a major regulator of eukaryotic cell growth. BMC Plant Biol. 2005 Feb 21;5:2.

18. Carlton JM, Hirt RP, Silva JC, Delcher AL, Schatz M, Zhao Q, et al. Draft genome sequence of the sexually transmitted pathogen Trichomonas vaginalis. Science. 2007 Jan 12;315(5809):207-12.

19. Malik SB, Pightling AW, Stefaniak LM, Schurko AM, Logsdon JM,Jr. An expanded inventory of conserved meiotic genes provides evidence for sex in Trichomonas vaginalis. PLoS One. 2008 Aug 6;3(8):e2879.

20. Malik SB, Ramesh MA, Hulstrand AM, Logsdon JM,Jr. Protist homologs of the meiotic Spo11 gene and topoisomerase VI reveal an evolutionary history of gene duplication and lineage-specific loss. Mol Biol Evol. 2007 Dec;24(12):2827-41.

21. Howard-Till RA, Lukaszewicz A, Novatchkova M, Loidl J. A single cohesin complex performs mitotic and meiotic functions in the protist tetrahymena. PLoS Genet. 2013 Mar;9(3):e1003418.

22. Urban E, Nagarkar-Jaiswal S, Lehner CF, Heidmann SK. The cohesin subunit Rad21 is required for synaptonemal complex maintenance, but not sister chromatid cohesion, during Drosophila female meiosis. PLoS Genet. 2014 Aug 7;10(8):e1004540.

23. Lee J, Hirano T. RAD21L, a novel cohesin subunit implicated in linking homologous chromosomes in mammalian meiosis. J Cell Biol. 2011 Jan 24;192(2):263-76.

24. da Costa-Nunes JA, Capitao C, Kozak J, Costa-Nunes P, Ducasa GM, Pontes O, et al. The AtRAD21.1 and AtRAD21.3 Arabidopsis cohesins play a synergistic role in somatic DNA double strand break damage repair. BMC Plant Biol. 2014 Dec 16;14:353,014-0353-9.

25. Mercier R, Jolivet S, Vezon D, Huppe E, Chelysheva L, Giovanni M, et al. Two meiotic crossover classes cohabit in Arabidopsis: one is dependent on MER3,whereas the other one is not. Curr Biol. 2005 Apr 26;15(8):692-701.

26. Shodhan A, Lukaszewicz A, Novatchkova M, Loidl J. Msh4 and Msh5 function in SC-independent chiasma formation during the streamlined meiosis of Tetrahymena. Genetics. 2014 Nov;198(3):983-93.

27. Grishaeva TM, Bogdanov YF. Conservation and variability of synaptonemal complex proteins in phylogenesis of eukaryotes. Int J Evol Biol. 2014;2014:856230.

28. Patil S, Moeys S, von Dassow P, Huysman MJ, Mapleson D, De Veylder L, et al. Identification of the meiotic toolkit in diatoms and exploration of meiosis-specific SPO11 and RAD51 homologs in the sexual species Pseudo-nitzschia multistriata and Seminavis robusta. BMC Genomics. 2015 Nov 14;16:930,015-1983-5.

29. von Besser K, Frank AC, Johnson MA, Preuss D. Arabidopsis HAP2 (GCS1) is a sperm-specific gene required for pollen tube guidance and fertilization. Development. 2006 Dec;133(23):4761-9.

30. Ning J, Otto TD, Pfander C, Schwach F, Brochet M, Bushell E, et al. Comparative genomics in Chlamydomonas and Plasmodium identifies an ancient nuclear envelope protein family essential for sexual reproduction in protists, fungi, plants, and vertebrates. Genes Dev. 2013 May 15;27(10):1198-215.

31. Alandete-Saez M, Ron M, Leiboff S, McCormick S. Arabidopsis thaliana GEX1 has dual functions in gametophyte development and early embryogenesis. Plant J. 2011 Nov;68(4):620-32.

32. Abrams EW, Zhang H, Marlow FL, Kapp L, Lu S, Mullins MC. Dynamic assembly of brambleberry mediates nuclear envelope fusion during early development. Cell. 2012 Aug 3;150(3):521-32.

33. Speijer D, Lukes J, Elias M. Sex is a ubiquitous, ancient, and inherent attribute of eukaryotic life. Proc Natl Acad Sci U S A. 2015 Jul 21;112(29):8827-34.

34. Eme L, Moreira D, Talla E, Brochier-Armanet C. A complex cell division machinery was present in the last common ancestor of eukaryotes. PLoS One. 2009;4(4):e5021.

35. Tonelli FM, Santos AK, Gomes DA, da Silva SL, Gomes KN, Ladeira LO, et al. Stem cells and calcium signaling. Adv Exp Med Biol. 2012;740:891-916.

36. Hodges ME, Wickstead B, Gull K, Langdale JA. Conservation of ciliary proteins in plants with no cilia. BMC Plant Biol. 2011 Dec 30;11:185,2229-11-185.

37. Rocha EP, Danchin A. Base composition bias might result from competition for metabolic resources. Trends Genet. 2002 Jun;18(6):291-4.

38. Duret L, Galtier N. Biased gene conversion and the evolution of mammalian genomic landscapes. Annu Rev Genomics Hum Genet. 2009;10:285-311.
