# Supplementary figures and images for: Extreme genome diversity in the hyper-prevalent parasitic eukaryote Blastocystis
Source: PLoS Biol. 2017 Sep 11;15(9):e2003769. doi: 10.1371/journal.pbio.2003769 (PMC5608401; doi:10.1371/journal.pbio.2003769)

**
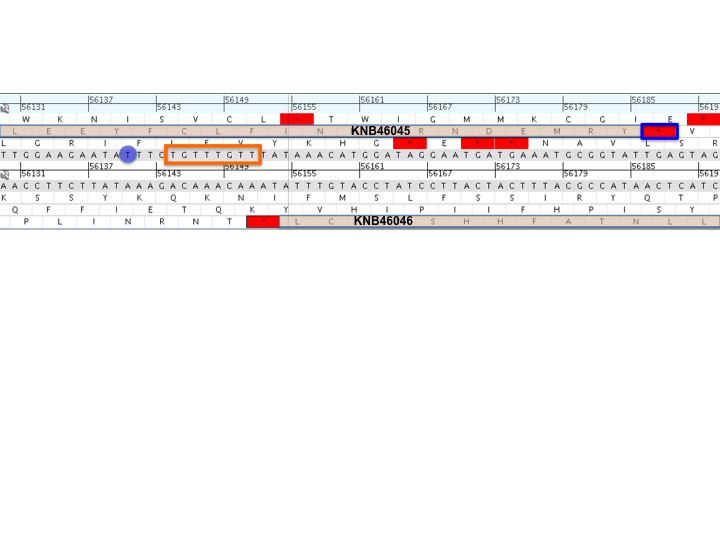
**

Supplement: S1 Fig — The 3′ ends of KNB46045 and KNB46046 overlap on scaffold NW_014569526. The presently annotated stop codon for KNB46045 (TGA) is represented by a red box with a dark blue outline. Where polyadenylation starts and creates an alternative stop codon (TAA) is indicated with a transparent blue circle and the motif (TGTTTGTT) that determines where the polyadenylation would start is denoted by an orange bordered box. (DOCX) [file pbio.2003769.s002.docx]

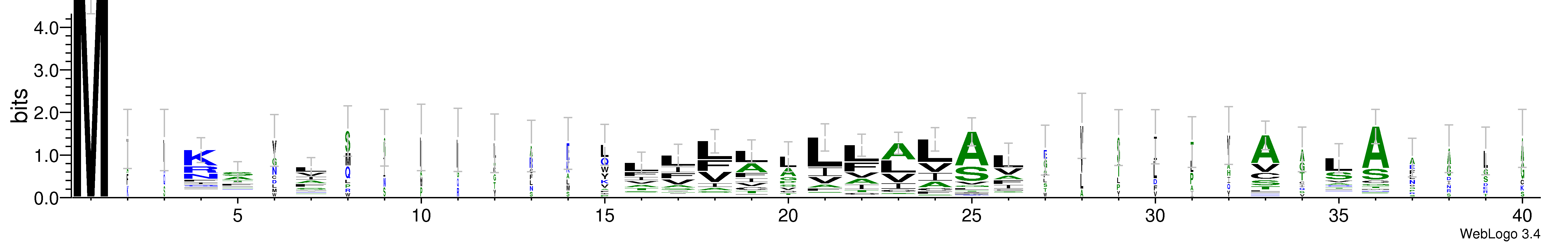

Supplement: S2 Fig — (DOCX) [file pbio.2003769.s003.docx]

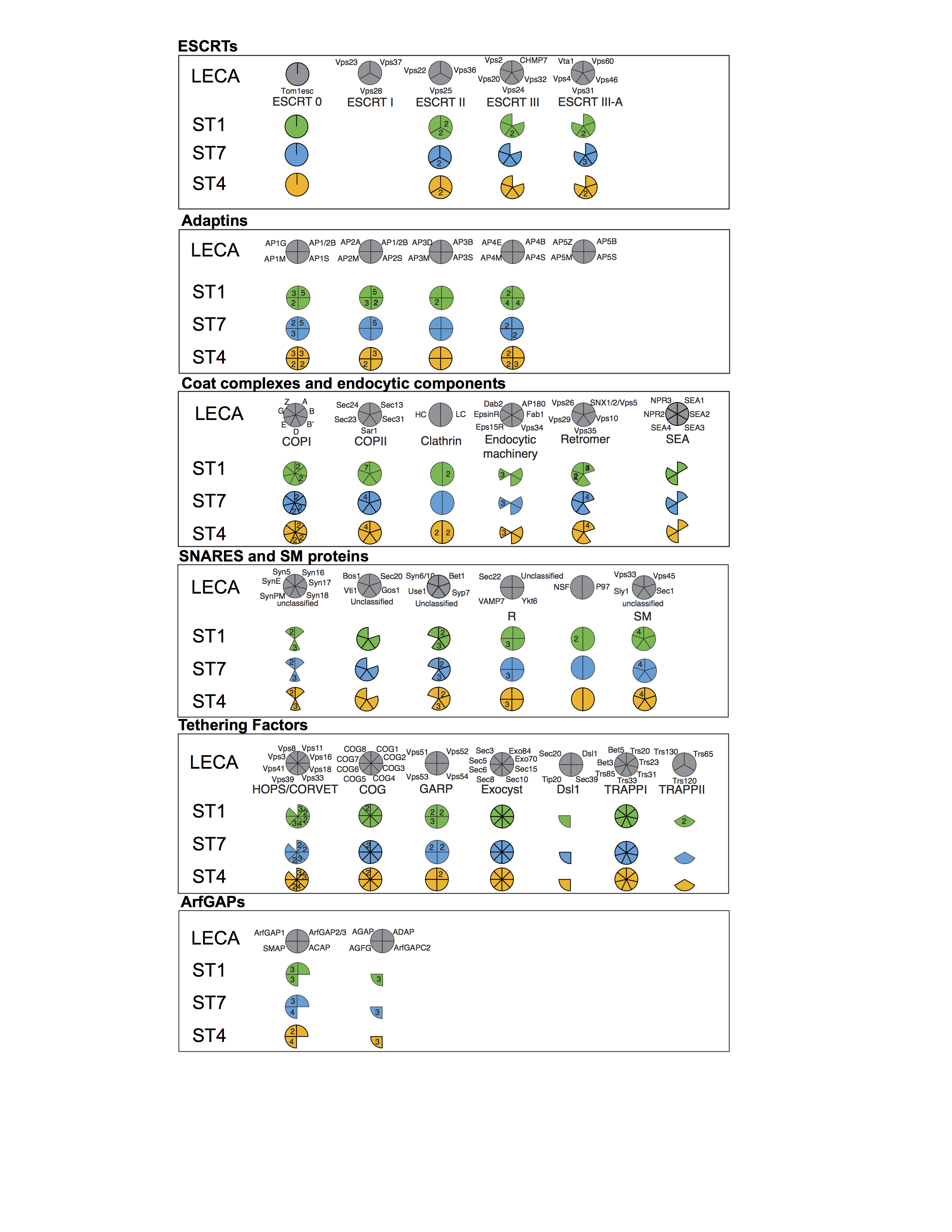

Supplement: S3 Fig — The numbers within the pie slices indicate the number of isoforms. (DOCX) [file pbio.2003769.s004.docx]

**
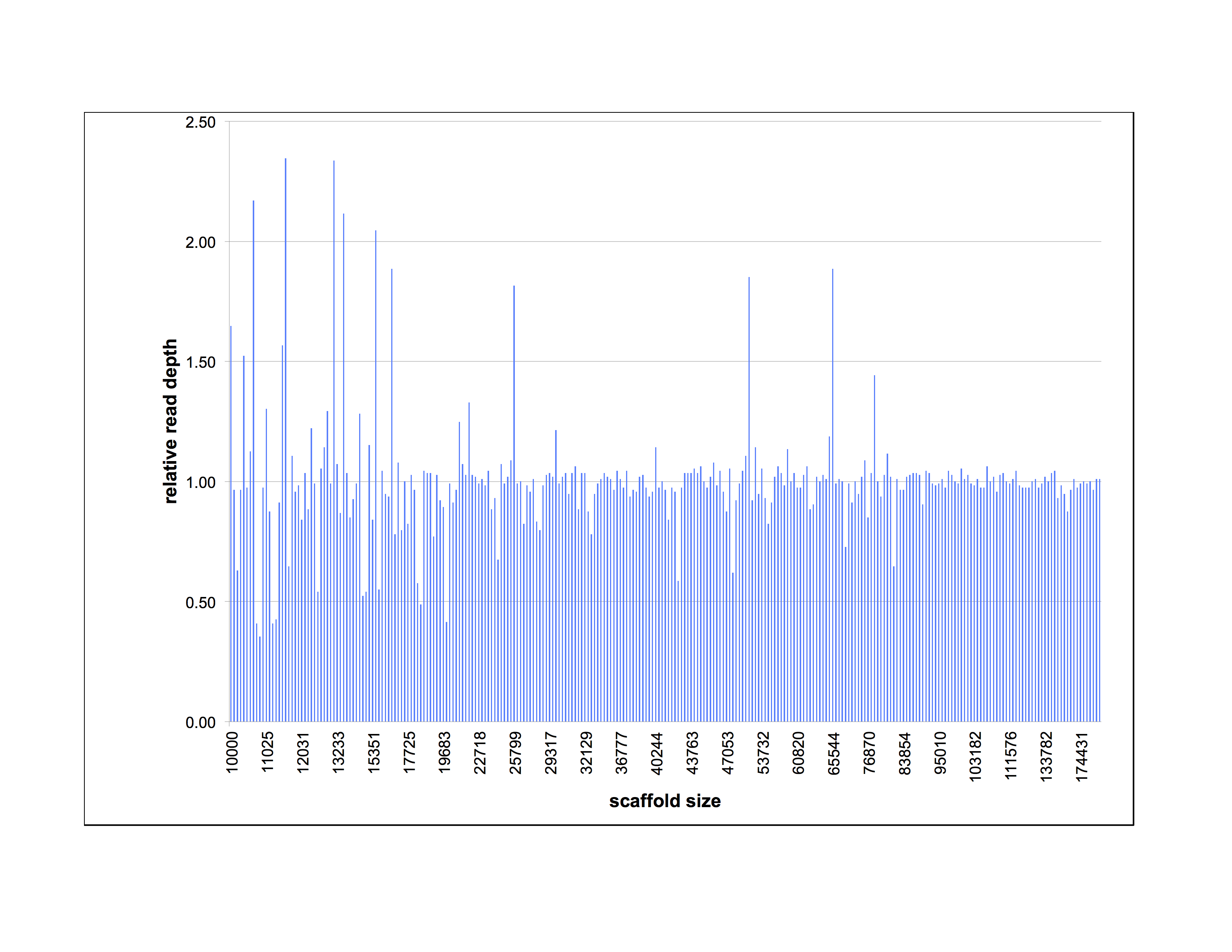
**

Supplement: S6 Fig — Read depth was scaled to give the median value (113) of the scaffold medians a value of 1.00. (DOCX) [file pbio.2003769.s007.docx]

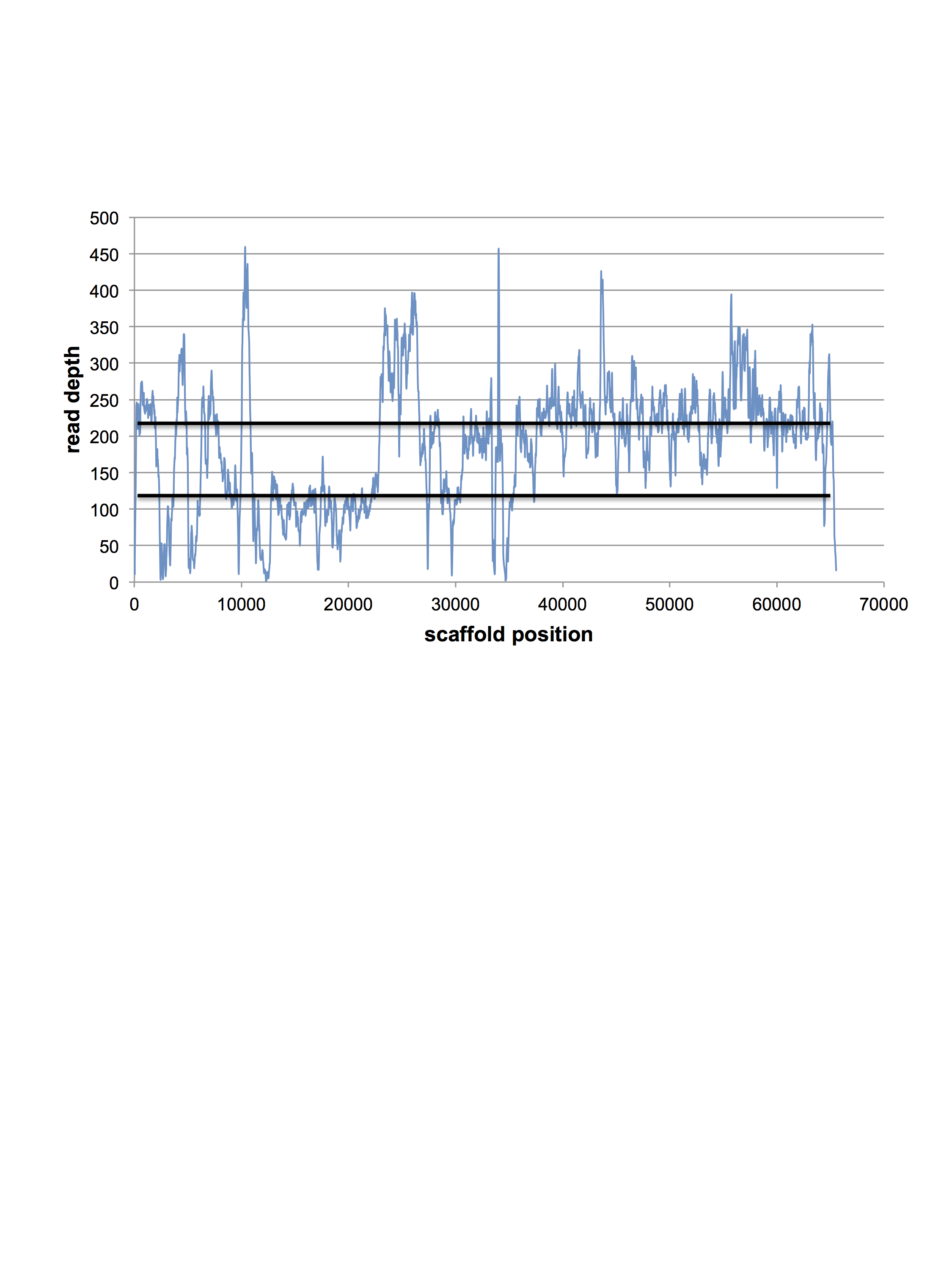

Supplement: S7 Fig — Y-axis corresponds to read depth, x-axis shows scaffold position. Upper bar denotes median read depth for scaffold 113 (213), while lower bar indicates median read depth (113) of all scaffolds over 10,000 bps. (DOCX) [file pbio.2003769.s008.docx]

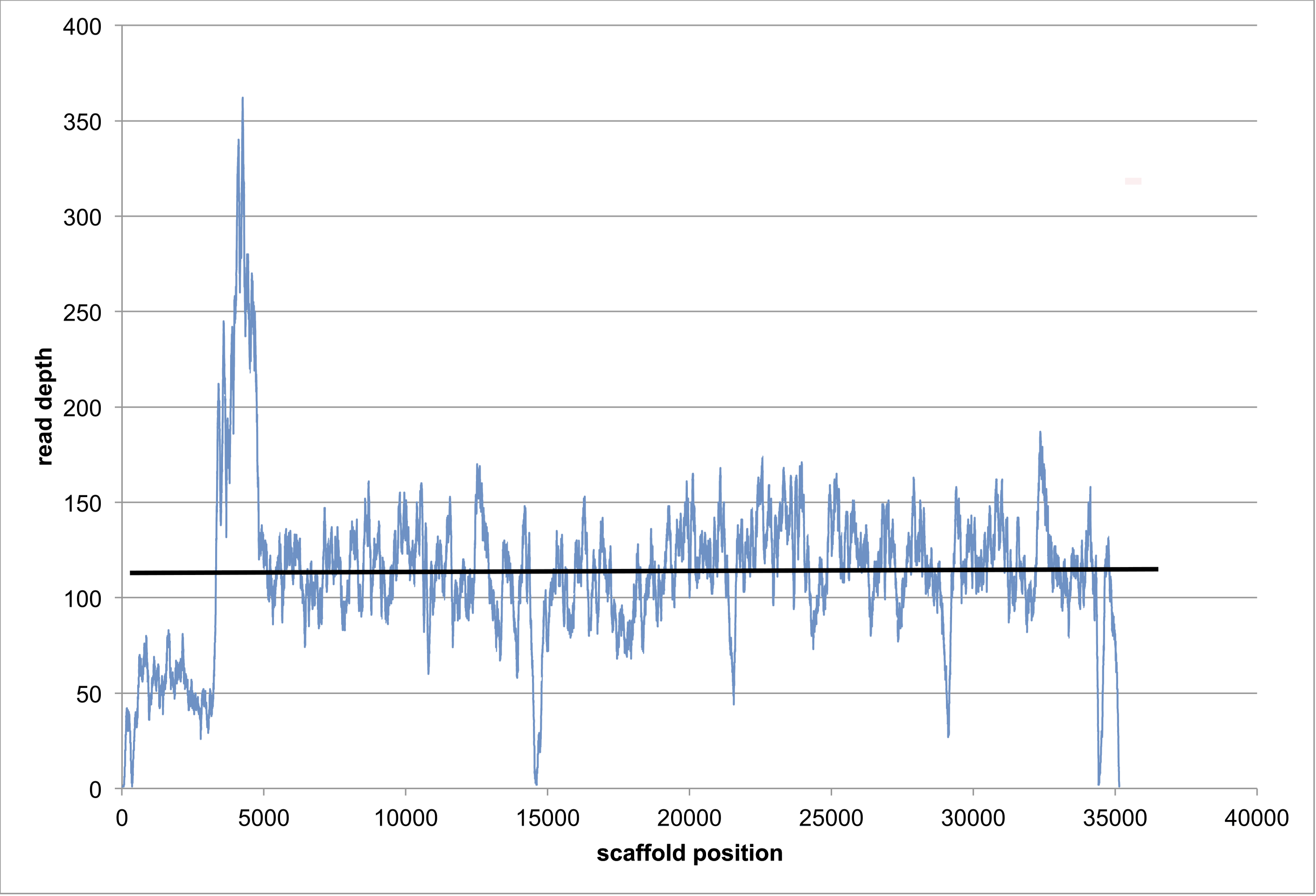

Supplement: S8 Fig — Y-axis corresponds to read depth, x-axis shows scaffold position. Horizontal bar denotes median read depth for scaffold 102 as well as indicating median read depth (113) of all scaffolds over 10,000 bps. (DOCX) [file pbio.2003769.s009.docx]

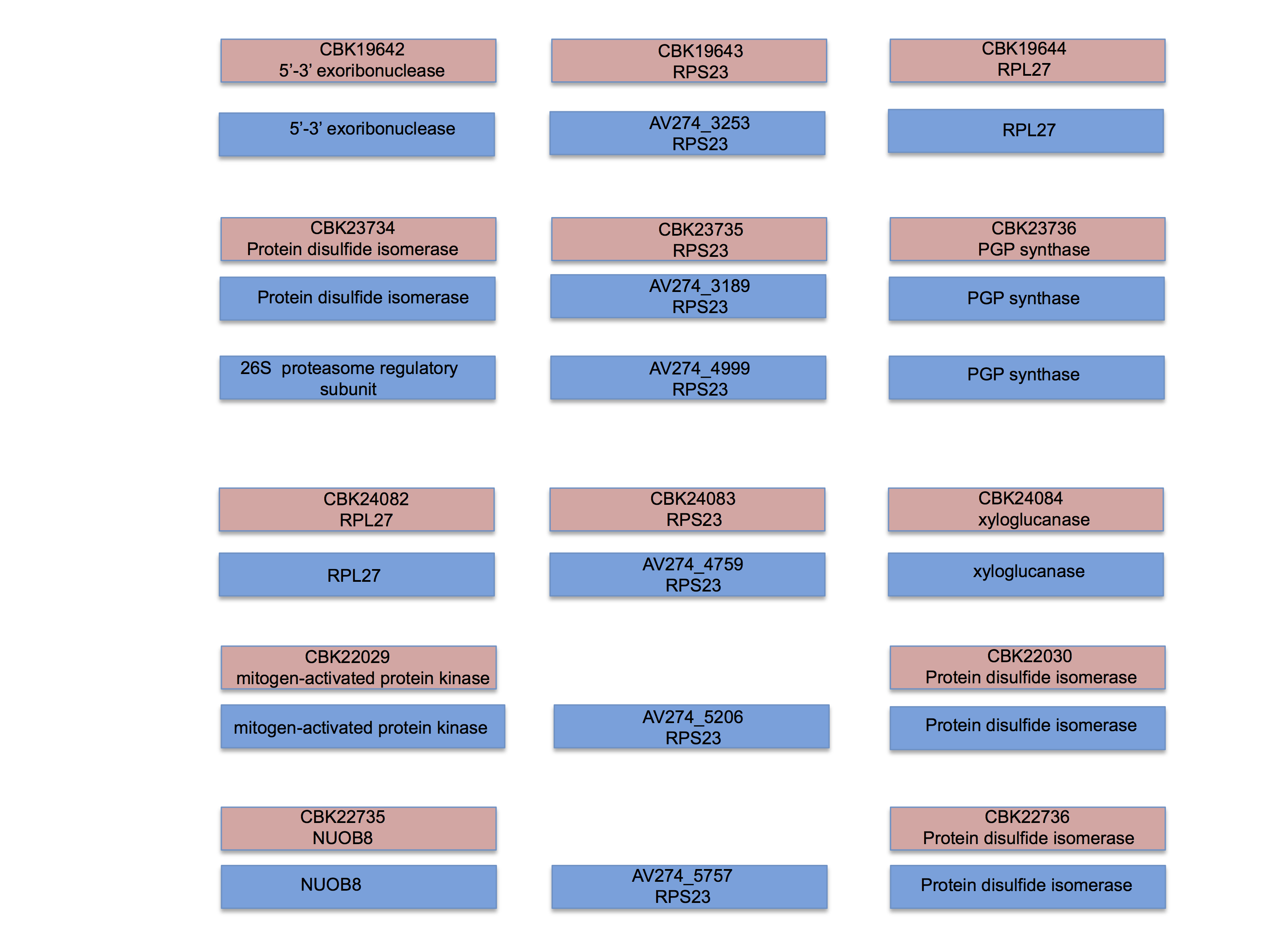

Supplement: S9 Fig — The middle gene corresponds to RPS23. ST1 genes are in blue, ST7 in red. ST1 has 6 copies of RPS23, all of which differ from each other at the nucleotide level. The 3 copies of RPS23 in ST7 are aligned with the corresponding copy in ST1 based on similar flanking genes. Two ST1 copies (AV274_5206 and AV274_5757) do not have a corresponding ST7 copy and, based on the arrangement of flanking genes, represent either insertions in ST1 or deletions in ST7. (DOCX) [file pbio.2003769.s010.docx]
