# Supplementary material for: Extreme genome diversity in the hyper-prevalent parasitic eukaryote Blastocystis
Source: PLoS Biol. 2017 Sep 11;15(9):e2003769. doi: 10.1371/journal.pbio.2003769 (PMC5608401; doi:10.1371/journal.pbio.2003769)
Supplement: S5 Fig — (DOCX) [file pbio.2003769.s006.docx]

**
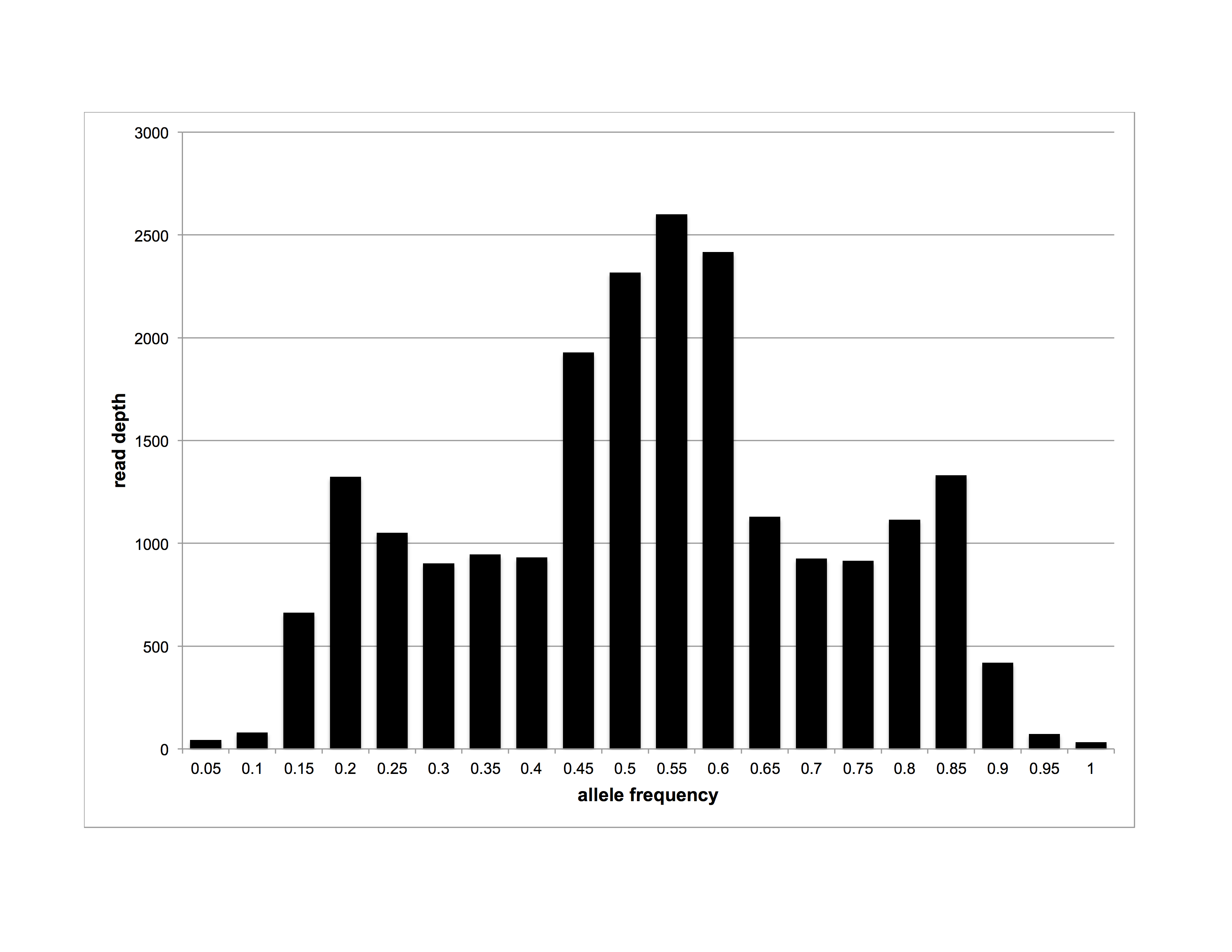
**

**Fig S5. Distribution of allele frequencies of heterozygous sites across *Blastocystis* ST1 genomic scaffolds.**
