# Supplementary material for: Extreme genome diversity in the hyper-prevalent parasitic eukaryote Blastocystis
Source: PLoS Biol. 2017 Sep 11;15(9):e2003769. doi: 10.1371/journal.pbio.2003769 (PMC5608401; doi:10.1371/journal.pbio.2003769)
Supplement: S1 Table — (DOCX) [file pbio.2003769.s012.docx]

**Table S1. Minor spliceosome-specific components in *Blastocystis* ST1 and ST7.**

| Minor spliceosome component | *Blastocystis* ST1 | *Blastocystis* ST7 |
| --- | --- | --- |
| 20K subunit | AV274_5781 | Unanotated gene on contig CABX01000004.1 |
| 25K subunit | AV274_3857 | Unanotated gene on contig CABX01000017.1 |
| 31K subunit | AV274_5379 | CBK19746.2 |
| 35K subunit | AV274_3816 | CBK22085.2 |
| 48K subunit | AV274_6003 | CBK22257.2 |
| 59K subunit | AV274_4636 | Unanotated gene on contig CABX01000028.1 |
| 65K | AV274_1471 | CBK20708.2 |
| U11 | AV274_4207 | Unanotated gene on contig CABX01000016.1 |
| U12 | AV274_4643 | Unanotated gene on contig CABX01000028.1 |
| U4atac | AV274_0166 | Unanotated gene on contig CABX01000005.1 |
| U6atac | scaffold191:20487-20602 (complement), scaffold197:3385-3500 | Unanotated gene on contig CABX01000019.1 |
