# Supplementary material for: Extreme genome diversity in the hyper-prevalent parasitic eukaryote Blastocystis
Source: PLoS Biol. 2017 Sep 11;15(9):e2003769. doi: 10.1371/journal.pbio.2003769 (PMC5608401; doi:10.1371/journal.pbio.2003769)
Supplement: S2 Table — (DOCX) [file pbio.2003769.s013.docx]

**Table S2. Median sequence identity of matching regions of orthologous proteins from pairs of protozoan pathogens**.

| Pair | Median sequence identity |
| --- | --- |
| *Trypanosoma* (*T. cruzi* - *T. brucei*) | 54% |
| *Blastocystis* (ST1-ST4) | 59% |
| *Blastocystis* (ST4-ST7) | 60% |
| *Blastocystis* (ST1-ST7) | 61% |
| *Plasmodium* (*P. falciparum* - *P. knowlesi*) | 61% |
| *Giardia (*WB-GS*)* | 81% |
| *Giardia* (GS-P15) | 81% |
| *Theileria* (*T. parva* - *T. annulata*) | 82% |
| *Giardia* (WB-P15) | 90% |
| *Leishmania* (*L. major* - *L. infantum*) | 93% |
| *Cryptosporidium* (*C. parvum* - *C. hominis*) | 98% |
