# Supplementary material for: Extreme genome diversity in the hyper-prevalent parasitic eukaryote Blastocystis
Source: PLoS Biol. 2017 Sep 11;15(9):e2003769. doi: 10.1371/journal.pbio.2003769 (PMC5608401; doi:10.1371/journal.pbio.2003769)
Supplement: S3 Table — (DOCX) [file pbio.2003769.s014.docx]

**Table S3. α-glucan** **related genes in *Cafeteria* transcriptomes.**

| ST1 gene | product | *Cafeteria* gene | Evalue |
| --- | --- | --- | --- |
| OAO18236 | glycogen synthase | CAMPEP_0196773098  *Cafeteria* sp., Strain Caron Lab Isolate | 4e-145 |
| OAO18236 | glycogen synthase | CAMPEP_0117703034  *Cafeteria roenbergensis*, Strain E4-10 | 3e-107 |
| OAO12628 | 1,4-alpha-glucan-branching enzyme | CAMPEP_0196788180  *Cafeteria* sp., Strain Caron Lab Isolate | 1e-152 |
| OAO12628 | 1,4-alpha-glucan-branching enzyme | CAMPEP_0117728188  *Cafeteria roenbergensis*, Strain E4-10 | 1e-131 |
