# Supplementary material for: Extreme genome diversity in the hyper-prevalent parasitic eukaryote Blastocystis
Source: PLoS Biol. 2017 Sep 11;15(9):e2003769. doi: 10.1371/journal.pbio.2003769 (PMC5608401; doi:10.1371/journal.pbio.2003769)
Supplement: S4 Table — (DOCX) [file pbio.2003769.s015.docx]

**Table S4. Telomerase related genes in *Blastocystis* STs 1, 4 and 7.**

| Gene | ST1 | ST4 | ST7 |
| --- | --- | --- | --- |
| TERT | AV274_0550 | XP_014529063 | XP_012896206 |
| DKC1 | AV274_2139, AV274_6146, AV274_0456 | XP_014525544 | XP_012896944, XP_012895277 |
| Est1 | AV274_4459,AV274_0813 | XP_01529204, XP_014530160 | XP_012894066, XP_012896001 |
