# Supplementary material for: Extreme genome diversity in the hyper-prevalent parasitic eukaryote Blastocystis
Source: PLoS Biol. 2017 Sep 11;15(9):e2003769. doi: 10.1371/journal.pbio.2003769 (PMC5608401; doi:10.1371/journal.pbio.2003769)
Supplement: S5 Table — (DOCX) [file pbio.2003769.s016.docx]

**Table S5. Telomeric repeats (TTAGGG) in *Blastocystis* ST1.**

| scaffold | position | No. of repeats | notes |
| --- | --- | --- | --- |
| scaffold489 | 5' | 39 |  |
| scaffold487 | 3' | 38 |  |
| scaffold472 | 5' | 21 |  |
| scaffold421 | interior | 29 | bounded by large gap |
| scaffold421 | 3' | 28 | 12 TTAGG |
| scaffold415 | 3' | 20 |  |
| scaffold403 | 5' | 30 |  |
| scaffold389 | interior | 25 | 9 TTAGG |
| scaffold385 | 5 | 68 |  |
| scaffold383 | 3' | 39 |  |
| scaffold36 | 3' | 28 |  |
| scaffold282 | interior | 33 |  |
| scaffold282 | interior | 40 |  |
| scaffold250 | 3' | 32 |  |
| scaffold241 | 3' | 35 |  |
| scaffold213 | 3' | 40 |  |
| scaffold144 | 3' | 15 |  |
