# Supplementary material for: Extreme genome diversity in the hyper-prevalent parasitic eukaryote Blastocystis
Source: PLoS Biol. 2017 Sep 11;15(9):e2003769. doi: 10.1371/journal.pbio.2003769 (PMC5608401; doi:10.1371/journal.pbio.2003769)
Supplement: S6 Table — (DOCX) [file pbio.2003769.s017.docx]

**Table S6. Copy numbers of 40S ribosomal proteins in *Blastocystis* ST1 and ST7.**

| 40S ribosomal proteins | ST7 copies | ST1 copies |
| --- | --- | --- |
| RPS1A | 6 | 6 |
| RPS4e | 3 | 2 |
| RPS5e | 2 | 6 |
| RPS6 | 4 | 6 |
| RPS7 | 3 | 3 |
| RPS8 | 7 | 8 |
| RPS 9 | 5 | 4 |
| RPS10 | 1 | 2 |
| RPS11 | 3 | 3 |
| RPS13-2 | 3 | 3 |
| RPS15 | 4 | 3 |
| RPS16 | 4 | 4 |
| RPS17 | 2 | 3 |
| RPS18 | 4 | 5 |
| RPS19e | 2 | 5 |
| RPS20 | 1 | 6 |
| RPS21 | 1 | 3 |
| RPS23 | 3 | 6 |
| RPS24 | 4 | 4 |
| RPS26e (A) | 4 | 5 |
| RPS27 | 1 | 3 |
| RPS28 | 2 | 3 |
| RPS29 | 2 | 3 |
| RPS30 | 3 | 4 |
| RPSSA | 6 | 4 |
