# Supplementary material for: Extreme genome diversity in the hyper-prevalent parasitic eukaryote Blastocystis
Source: PLoS Biol. 2017 Sep 11;15(9):e2003769. doi: 10.1371/journal.pbio.2003769 (PMC5608401; doi:10.1371/journal.pbio.2003769)
Supplement: S7 Table — (DOCX) [file pbio.2003769.s018.docx]

**Table S7. Copy numbers of 60S ribosomal proteins in *Blastocystis* ST1 and ST7.**

|  | ST7 | ST1 |
| --- | --- | --- |
| RPL3 | 4 | 4 |
| RPL4 | 3 | 3 |
| RPL5 | 3 | 4 |
| RPL6 | 3 | 4 |
| RPL7 | 6 | 6 |
| RPL7A | 6 | 2 |
| RPL8 | 8 | 8 |
| RPL9 | 4 | 4 |
| RPL10 | 3 | 7 |
| RPL10A | 3 | 4 |
| RPL11 | 5 | 9 |
| RPL12 | 4 | 5 |
| RPL13 | 5 | 5 |
| RPL13A | 3 | 6 |
| RPL14 | 2 | 2 |
| RPL15 | 3 | 6 |
| RPL17 | 4 | 4 |
| RPL18 | 3 | 7 |
| RPL18A | 3 | 6 |
| RPL19 | 4 | 2 |
| RPL21 | 3 | 5 |
| RPL22 | 3 | 3 |
| RPL23 | 3 | 2 |
| RPL23A | 3 | 2 |
| RPL24 | 4 | 4 |
| RPL26 | 3 | 4 |
| RPL27 | 2 | 4 |
| RPL27A | 3 | 4 |
| RPL28 | 0 | 3 |
| RPL29 | 2 | 2 |
| RPL30 | 2 | 2 |
| RPL31 | 2 | 2 |
| RPL32 | 3 | 4 |
| RPL34 | 2 | 4 |
| RPL35 | 2 | 4 |
| RPL35A | 2 | 2 |
| RPL36 | 3 | 3 |
| RPL36A (L44, L42A) | 3 | 3 |
| RPL37 | 3 | 2 |
| RPL37A | 3 | 3 |
| RPL38 | 3 | 1 |
| RPL39 | 2 | 4 |
| RPL40 | 4 | 4 |
| RPLP0 | 2 | 4 |
| RPLP2 | 6 | 4 |
