# Supplementary material for: Extreme genome diversity in the hyper-prevalent parasitic eukaryote Blastocystis
Source: PLoS Biol. 2017 Sep 11;15(9):e2003769. doi: 10.1371/journal.pbio.2003769 (PMC5608401; doi:10.1371/journal.pbio.2003769)
Supplement: S8 Table — Abbreviations: CBM, carbohydrate-binding modules; CE, carbohydrate esterases; GH, glycosyl hydrolases; GT, glycosyl transferases; PL, polysaccharide lyase. (DOCX) [file pbio.2003769.s019.docx]

**Table S8**. **CAZymes present in *Blastocystis* ST1 and selected stramenopile genomes.** GH= Glycosyl Hydrolases, GT= Glycosyl Transferases, PL= Polysaccharide Lyase, CE= Carbohydrate Esterases, CBM= Carbohydrate-Binding Modules.

| **Species** | **GH** | **GT** | **PL** | **CE** | **CBM** | **GH+GT** |
| --- | --- | --- | --- | --- | --- | --- |
| *Blastocystis* ST1 | 49 | 132 | 1 | 3 | 18 | 181 |
| *Ectocarpus siliculosus* | 64 | 154 | 4 | 4 | 61 | 218 |
| *Thalassiosira pseudonana* | 62 | 92 | 0 | 3 | 9 | 154 |
| *Albugo laibachii* Nc14 | 88 | 68 | 0 | 5 | 10 | 156 |
| *Phytophthora infestans* T30-4 | 283 | 157 | 67 | 23 | 39 | 440 |
