# Supplementary material for: Extreme genome diversity in the hyper-prevalent parasitic eukaryote Blastocystis
Source: PLoS Biol. 2017 Sep 11;15(9):e2003769. doi: 10.1371/journal.pbio.2003769 (PMC5608401; doi:10.1371/journal.pbio.2003769)
Supplement: S9 Table — (DOCX) [file pbio.2003769.s020.docx]

**Table S9. Number of different CAZy families found in *Blastocystis* ST1 and selected stramenopile genomes.**

| **CAZy families** | ***Blastocystis* ST1** | ***Ectocarpus***  ***siliculosus*** | ***Thalassiosira pseudonana*** | ***Albugo laibachii* Nc14** | ***Phytophthora infestans* T30-4** |
| --- | --- | --- | --- | --- | --- |
| **GHs** | 21 | 22 | 21 | 20 | 33 |
| **GTs** | 22 | 38 | 28 | 21 | 25 |
