# Supplementary material for: Extreme genome diversity in the hyper-prevalent parasitic eukaryote Blastocystis
Source: PLoS Biol. 2017 Sep 11;15(9):e2003769. doi: 10.1371/journal.pbio.2003769 (PMC5608401; doi:10.1371/journal.pbio.2003769)
Supplement: S10 Table — (DOCX) [file pbio.2003769.s021.docx]

**Table S10. *Blastocystis* ST1 protein models with matches to a *Chlamydomonas reinhardtii* flagellar data set.**

| *Blastocystis* ST1 protein id | *Blastocystis* ST1 annotation | *C. reinhardtii* protein id | *C. reinhardtii* annotation |
| --- | --- | --- | --- |
| AV274_0254 | ATP-dependent RNA helicase DHX34 | XP_001689776 | DEAD/DEAH box helicase |
| AV274_0282 | hypothetical protein | XP_001690930 | flagellar associated protein |
| AV274_1094 | serine/threonine-protein kinase Nek1-like protein | XP_001700340 | hypothetical protein |
| AV274_1533 | tenascin XB | XP_001691097 | hypothetical protein |
| AV274_2750 | hypothetical protein | XP_001690325 | hypothetical protein |
| AV274_4406 | MORN repeat-containing protein precursor | XP_001699180 | flagellar associated protein |
| AV274_5298 | glycoprotein endo-alpha-1,2-mannosidase-like protein | XP_001691860 | hypothetical protein |
