# Supplementary material for: Extreme genome diversity in the hyper-prevalent parasitic eukaryote Blastocystis
Source: PLoS Biol. 2017 Sep 11;15(9):e2003769. doi: 10.1371/journal.pbio.2003769 (PMC5608401; doi:10.1371/journal.pbio.2003769)
Supplement: Supplementary file 14 [file pbio.2003769.s005.docx]

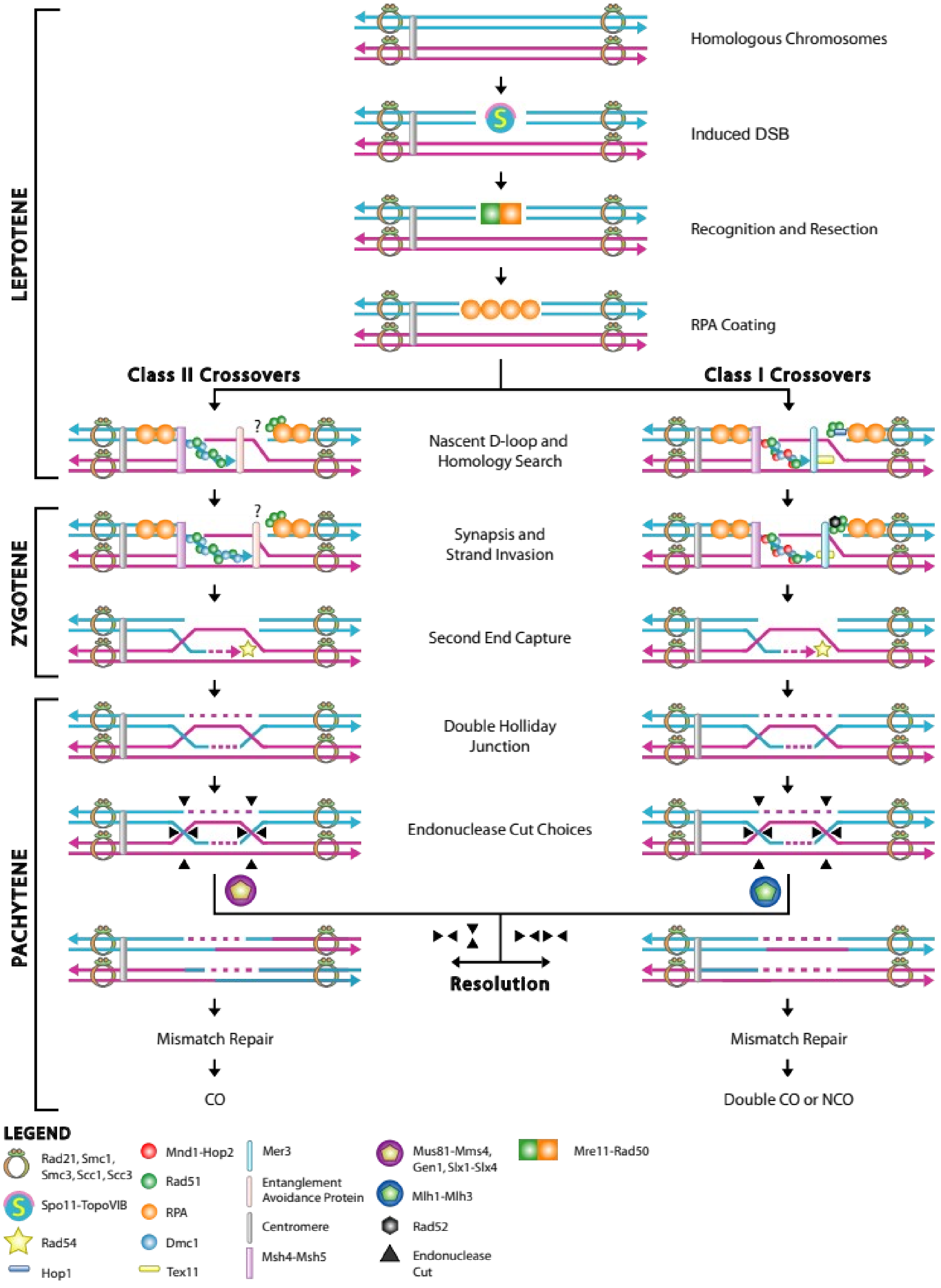
LEPTOTENE
ZYGOTENE
PACHYTENE
Homologous Chromosomes
Induced DSB
Recognition and Resection
RPA Coating
Class II Crossovers
Class I Crossovers
Nascent D-loop and Homology Search
Synapsis and Strand Invasion
Second End Capture
Double Holliday Junction
Endonuclease Cut Choices
Resolution
Mismatch Repair
CO
Mismatch Repair
Double CO or NCO
LEGEND
Rad21, Smc1, Smc3, Scc1, Scc3
Spo11-TopoVIB
Rad54
Hop1
Mnd1-Hop2
Rad51
RPA
Dmc1
Tex11
Mer3
Entanglement Avoidance Protein
Centromere
Msh4-Msh5
Mus81-Mms4, Gen1, Slx1-Slx4
Mlh1-Mlh3
Rad52
Endonuclease Cut
Mre11-Rad50
