# Supplementary material for: Extreme genome diversity in the hyper-prevalent parasitic eukaryote Blastocystis
Source: PLoS Biol. 2017 Sep 11;15(9):e2003769. doi: 10.1371/journal.pbio.2003769 (PMC5608401; doi:10.1371/journal.pbio.2003769)
Supplement: Supplementary file 15 [file pbio.2003769.s011.docx]

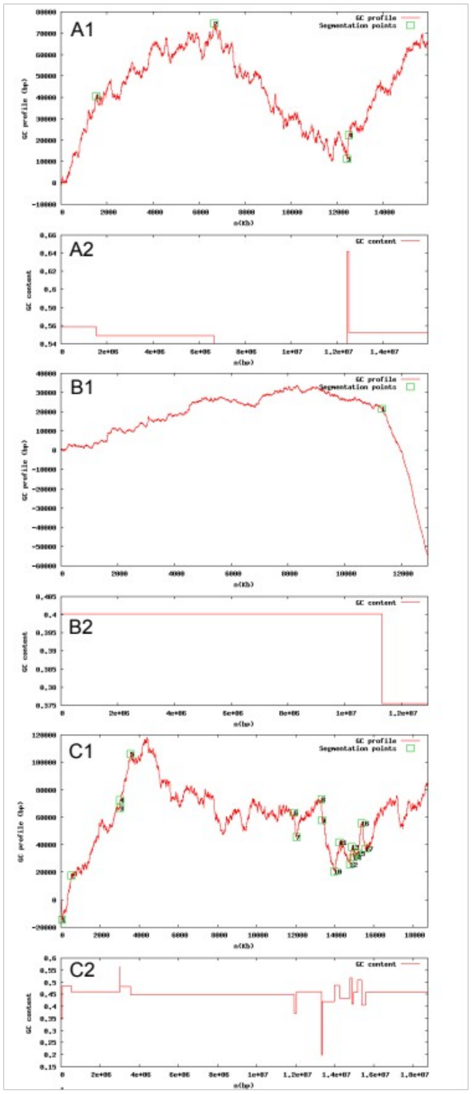
A1
GC profile
Segmentation points
GC profile (bp)
n(kb)
A2
GC content
GC content
n(bp)
B1
GC profile
Segmentation points
GC profile (bp)
n(kb)
B2
GC content
GC content
n(bp)
C1
GC profile
Segmentation points
GC profile (bp)
n(kb)
C2
GC content
GC content
n(bp)
